# Dissecting definitions of disability accrual in relapsing multiple sclerosis—Have we reached standardization yet?

**DOI:** 10.1177/13524585251396283

**Published:** 2025-12-06

**Authors:** Gabriel Bsteh, Stefanie Marti, Helly Hammer, Nik Krajnc, Michael Guger, Franziska Di Pauli, Jörg Kraus, Christian Enzinger, Andrew Chan, Thomas Berger, Harald Hegen, Robert Hoepner

**Affiliations:** Department of Neurology, Medical University of Vienna, Vienna, Austria; Comprehensive Center for Clinical Neurosciences and Mental Health, Medical University of Vienna, Vienna, Austria; Department of Neurology, Inselspital University Hospital Bern, Bern, Switzerland; Department of Neurology, Inselspital University Hospital Bern, Bern, Switzerland; Department of Neurology, Medical University of Vienna, Vienna, Austria; Comprehensive Center for Clinical Neurosciences and Mental Health, Medical University of Vienna, Vienna, Austria; Department of Neurology, Pyhrn-Eisenwurzen Hospital Steyr, Steyr, Austria; Department of Neurology, Medical University of Innsbruck, Innsbruck, Austria; Department of Laboratory Medicine, Paracelsus Medical University and Salzburger Landeskliniken, Salzburg, Austria; Department of Neurology, Medical Faculty, Heinrich-Heine University, Düsseldorf, Germany; Department of Neurology, Medical University of Graz, Graz, Austria; Department of Neurology, Inselspital University Hospital Bern, Bern, Switzerland; Department of Neurology, Medical University of Vienna, Vienna, Austria; Comprehensive Center for Clinical Neurosciences and Mental Health, Medical University of Vienna, Vienna, Austria; Department of Neurology, Medical University of Innsbruck, Innsbruck, Austria; Department of Neurology, Inselspital University Hospital Bern, Bern, Switzerland

**Keywords:** Multiple sclerosis, disability, progression, definition, PIRA, RAW

## Abstract

**Background:**

Distinguishing relapse-associated worsening (RAW) and progression independent of relapse activity (PIRA) has reshaped understanding of disability accumulation in relapsing multiple sclerosis (RMS). The influence of differing definitions of disability accrual on event rates and RAW/PIRA proportions remains uncertain.

**Methods:**

This observational cohort study used Austrian MS Treatment Registry data (2010–2024). A custom algorithm evaluated 1440 definitional variants of disability accrual with varying confirmation duration, baseline modeling, and RAW/PIRA classification, including recently proposed “standardized” criteria.

**Results:**

We included 3273 RMS patients (mean age 37.5 years; 67.8% female) with ⩾24 months follow-up, ⩾3 Expanded Disability Status Scale (EDSS) scores, and ⩾1 EDSS score per year, contributing 3525 follow-up periods. Depending on definition, disability accrual varied between 15.7% and 41.6% of follow-ups. PIRA accounted for 56.1%–86.3% of events across definitions, while up to 8.4% were ambiguously classified, mainly due to post-relapse re-baselining or relapses during the confirmation period. Even under “standardized” criteria, 144 definitional combinations remained, with event rates ranging from 19.1% to 21.7% and PIRA contribution varying widely from 59.8% to 85.8%.

**Conclusion:**

PIRA predominantly drives disability accrual, yet definitional variation substantially influenced event rates and RAW/PIRA proportions. Transparent reporting and further optimization of definitions are critical for improving comparability, interpretation, and clinical relevance in MS research and care.

## Introduction

In relapsing multiple sclerosis (RMS), disability worsening was traditionally attributed to incomplete recovery from relapses, while worsening without relapses—termed “progression”—was considered exclusive to progressive MS (PMS). However, mounting evidence indicates that “progression” can occur even in the earliest stages of RMS, now termed “progression independent of relapse activity” (PIRA), in contrast to “relapse-associated worsening” (RAW).^1–3^

The Expanded Disability Status Scale (EDSS) remains the most widely used disability endpoint in both clinical trials and real-world studies.^4–6^ Definitions of EDSS-based disability accrual vary across studies, for example, in terms of minimal score change required, the use of fixed versus roving baselines, and the requirement for confirmation of worsening. Further complicating matters, no uniform standard exists for classifying disability accrual as PIRA or RAW, with multiple—sometimes conflicting—definitions in use.^7,8^

These differences in defining disability accrual may significantly impact study outcomes, particularly in real-world observational studies that rely on diverse data sources, and can lead to ambiguity in clinical practice.^9^

Recent proposals to harmonize or standardize PIRA—while providing a highly valuable basis—do not cover all definitional aspects relevant to categorizing events as PIRA or RAW and clinical uncertainty due to missing data or the presence of relapses in proximity to confirmation assessments.^9^

In real-world settings, the frequency of disability events that cannot be clearly attributed to either PIRA or RAW remains unknown.

Here, we aimed to quantify the frequency of PIRA and RAW in a large, representative real-world cohort, focusing on how differences in definitions of disability accrual affect their rates, mutual relationship, and the proportion of events for which a clear distinction between PIRA and RAW is not possible.

## Methods

### Data preprocessing

For this retrospective study, we analyzed prospectively collected data from the Austrian MS Treatment Registry (AMSTR) between January 2010 and December 2024. We screened for patients aged ⩾ 18 years with RMS and (1) available clinical follow-up ⩾ 24 months, (2) ⩾1 EDSS assessment per year, and (3) ⩾3 available EDSS scores. If individuals had multiple follow-up periods fulfilling the above criteria, each period was included as a separate follow-up period. Two subgroup analyses were performed: (1) follow-up periods with at least one documented relapse during the follow-up period, and (2) follow-up periods with ⩾4 available EDSS scores and ⩾1 assessment per 6 months.

### Definitions of RAW and PIRA

For the present analysis, we required a minimum increase in EDSS of ⩾1.5/1.0/0.5 if the reference EDSS is 0/1–5.0/⩾5.5.^7^ No minimal distance to the reference was required, and we chose the confirmation condition as scores ⩾ reference + minimal increase. For confirmation times and included values, as well as for baselines, we analyzed several options (Table 1). The event score is the confirmed score, for example, if an increase from 3.0 to 4.5 is confirmed by a score of 4.0, then the event score is 4.0.

**Table 1.** Tested disability accrual and RAW/PIRA definition options. **Allowing relapses during the confirmation interval for PIRA is only allowed if confirmation is required and if only the last value in the confirmation interval is considered. See Supplemental Methods for a detailed description of the tested options**.

Option	Tested parameter choices	n
Event merging	True (max. repetition time = post-relapse RAW window size, no max. dist.) False	2
Undefined events	Re-baselining (only re-baselining assessments can be undefined worsening) Never (undefined worsening events are ignored) All (all assessments can be undefined worsening) End (all re-baselining assessments and all assessments after the last RAW/PIRA)	4
Constraints for undefined	Greater only Equal or greater Unconstrained	3
Baseline	Fixed baseline Next-confirmed roving reference	2
Minimal increase	+ 1.5 for reference 0, + 1.0 for reference < 5.5, + 0.5 else	1
Confirmation	None 12 weeks all confirmed 12 weeks last confirmed 24 weeks all confirmed 24 weeks last confirmed Sustained (no minimal duration) Sustained, minimal post-event follow-up duration of at least 12 weeks Sustained, minimal post-event follow-up duration of at least 24 weeks	8
Confirmation type	Minimum	1
Require confirmation for last visit	True	1
Left hand side confirmation tolerance	0 (i.e. earliest confirmation assessment for confirmation at x days is at day x)	1
Right hand side confirmation tolerance	∞ (i.e. confirmation assessment can be any time after the minimal distance)	1
RAW window size	30 days pre-relapse, 30 days post-relapse 30 days pre-relapse, 90 days post-relapse 90 days pre-relapse, 90 days post-relapse	3
Allow relapses in confirmation interval	True (only for last confirmed) False	2
Minimal distance	No minimal distance requirement	1
Total tested options		**1440**

Abbreviations: PIRA: Progression Independent of Relapse Activity. RAW: Relapse Associated Worsening.

The definition of RAW and PIRA comprises several domains implemented in a custom algorithm (for details, see Supplemental Methods):

1. Disability accrual.
  (a) Baseline selection: fixed versus roving reference, and confirmation of reference including the corresponding confirmation requirements.
  (b) Magnitude requirements: the minimal required magnitude of EDSS increase and whether the minimal required magnitude depends on the reference score.
  (c) Minimal distance requirements: the minimal required distance to the reference or the previous assessment.
  (d) Confirmation requirements:
    - Minimal confirmation time, whether an increase must be sustained over the entire follow-up period, and minimum required post-event follow-up duration.
    - Confirmation condition (confirmation scores ⩾ event score or confirmation scores ⩾ reference + magnitude requirement).
    - Confirmation inclusion (all values within the confirmation interval have to satisfy the confirmation condition versus only the first score satisfying the minimal confirmation distance condition has to satisfy the confirmation condition).

2. Rules for post-event and post-relapse re-baselining.
3. Time window around relapses (“RAW window”) to distinguish RAW from PIRA.
4. Rules for handling events that are not classifiable as either RAW or PIRA.
5. Rules for the interpretation of sequential events.^3,7,9^

#### Event types

**Figure 1.**
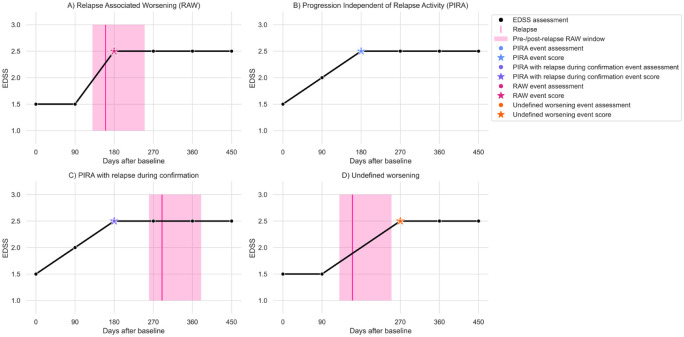
Examples of Relapse Associated Worsening (RAW, Panel A), Progression Independent of Relapse Activity (PIRA, Panel B), PIRA with relapse during confirmation (Panel C), and Undefined Worsening (Panel D). Panel C: the confirmation assessment is within the RAW window of the next relapse. The event at day 180 thus does not fulfill PIRA criteria, but cannot be considered RAW, either. It is thus labeled as PIRA with relapse during confirmation. Panel D: the post-relapse re-baselining assessment is itself an event. It is not possible to classify the event at day 270 as either PIRA or RAW, thus it is labeled as Undefined Worsening. Black dots represent EDSS scores, the pink vertical line indicates a relapse, and the pink shaded area indicates the RAW window, the period in which an event is considered relapse-associated (typically 30 days before until 90 days after a relapse). Minimal required increase + 1.0 EDSS points, confirmation at the next assessment required. Blue, purple, red, and orange dots indicate the assessment at which a disability accrual of the respective type was detected, and blue, purple, red, and orange stars indicate the corresponding event scores.

An event is classified as **RAW** (Figure 1A), if it

1. occurs in proximity of a relapse, for example, within 30 days prior until 90 days after a relapse (within the “RAW window”) and
2. is recorded with respect to a reference adjusted for residual disability from previous relapses (“RAW/PIRA reference”), and
3. is not at a post-relapse re-baselining assessment (first assessment after RAW window).

An event is classified as **PIRA** (Figure 1B), if

1. it occurs outside the RAW window and
2. it is recorded with respect to the RAW/PIRA reference, and
3. it is not a post-relapse re-baselining assessment, and
4. the confirmation assessment(s) are not in a RAW window.

An event is classified as **PIRA with relapse during confirmation** (Figure 1C), if

1. it fulfils the definition of PIRA.
2. confirmation assessment(s) are in proximity of a relapse, that is, in a RAW window.

An event fulfilling neither RAW nor PIRA criteria is classified as **Undefined Worsening** (Figure 1D). In contrast to PIRA with relapse during confirmation, undefined worsening events are an issue of follow-up quality, usually due to missing assessments in proximity of a relapse.

For a detailed description of the definition, implementation details, and available options, see Supplemental Methods.

#### Re-baselining rules

The baseline for RAW and PIRA is reset:

1. After each disability accrual event: the confirmed event score becomes the new baseline (eFigure 1A), and/or
2. After a relapse if residual disability is present (eFigure 1B). This residual disability does not require confirmation. If the post-relapse score is ⩽ the previous reference, the baseline remains unchanged unless a roving reference is used, in which case the lower score serves as a new reference (with optional confirmation). An example follow-up period with post-event and post-relapse re-baselining and all four event types is presented in eFigure 2.

#### Rules for undefined events

Our algorithm covers four options to annotate undefined worsening:

1. Only post-relapse re-baselining assessments can be undefined worsening (“re-baselining only”).
2. Ignore undefined worsening (“never”), that is, scores at post-relapse re-baselining assessments are not checked for worsening; yields the same number of RAW/PIRA events as option (1), but no undefined progression events.
3. All assessments can be undefined progression (“all”; after checking them for RAW/PIRA, so that RAW/PIRA take precedence over undefined).
4. Only post-relapse re-baselining assessments and assessments after the last RAW/PIRA event can be undefined progression (“end”).

#### Sequential events and event merging

Sequential events of the same type (typically PIRA) can be annotated as individual events or merged into a single event with higher ΔEDSS (eFigure 78). The algorithm supports maximal merge distance constraints, and sequential assessments with the same score can be either treated as stabilization (ends the merging) or repetition measurement (merging continues), with configurable maximum repetition intervals.

### Tested options

We tested 1440 combinations of definition aspects, covering commonly used options for baselines, confirmation criteria, and distance to relapses as well as all options for the handling of undefined events.

Our algorithm identifies PIRA events alongside RAW events and the additional categories “PIRA with relapse during confirmation” and “undefined worsening” for ambiguous events. Therefore, our algorithm includes parameters for event detection and classification that are not explicitly mentioned (or not required) for the proposed *harmonized PIRA* definition or the proposed *standardized PIRA* definition.^7,9^ Consequently, some ambiguities remain: within *harmonized* PIRA definition 360 combinations of parameters not specified remain due to handling of undefined events, roving reference confirmation, and event confirmation.^7^ The proposed *standardized* definition of PIRA still allows 144 combinations of parameter values not specified (details shown in Supplemental Materials).^7,9^

### Definition of contribution to total ΔEDSS

The contribution of disability accrual events of a given type to the total increase of EDSS on cohort level (ΔEDSS) was defined as the sum of the event ΔEDSS (confirmed event score minus reference score) over all events of the respective type divided by the sum of all event ΔEDSS over all events irrespective of type.

### Statistical methods and software

All data preprocessing and analysis were done in Python 3.12 with the NumPy and Pandas packages for data transformation, the lifelines package for survival analysis, and the matplotlib and seaborn packages for data visualization. The code for disability accrual annotation was written in Python 3.12 and is publicly available. We used Kaplan-Meier estimates for median time to event, and a permutation test (SciPy package) to test for differences between parameter choices. Sensitivity analyses were conducted for all disease-modifying treatment (DMT) at baseline that were present in ⩾100 follow-up periods using a leave-one-out approach.

### Ethics

The study was approved by the ethics committee of the Medical University Vienna (ethical approval number: 1668/2023). As data sets were obtained in routine practice and exported pseudonymously, the need for written informed consent from study participants was waived by the ethics committee. This study adheres to the reporting guidelines outlined within the “Strengthening the Reporting of Observational Studies in Epidemiology” (STROBE) Statement.

### Code and data availability statement

Our algorithm is publicly available at https://github.com/drstrupf/multiple-sclerosis-disability-progression under the MIT license. Individual definition aspects can be explored in an interactive web application available at https://multiple-sclerosis-disability-progression.streamlit.app/.

Anonymized data supporting the findings of this study are available from the corresponding author upon reasonable request by a qualified researcher and upon approval by the data-clearing committee of the Medical Universities of Vienna.

## Results

### Cohort

We analyzed 3525 follow-up periods from 3273 people with RMS (2207 [67.4%] female). Mean age at baseline was 37.5 years (range 18.0–73.0, IQR 30.0–45.0). Median baseline EDSS was 2.0 (range 0.0–8.5, IQR 1.0–3.0). The number of follow-up periods satisfying the inclusion criteria per person was: one in 3031 (92.6%), two in 232 (7.1%), and three in 10 individuals (0.3%). Median duration of follow-up periods was 4.3 years (range 2.0–16.2, IQR 3.0–6.6), with a median 13 EDSS assessments (range 4–54, IQR 9–20). At least one relapse occurred in 1361 (38.6%) of the follow-up periods. Of these, 687 (50.5%) had one relapse, 318 (23.4%) had two, 166 (12.2%) had three, 90 (6.6%) had four, and 100 (7.3%) had five or more relapses.

### Event rates

**Figure 2.**
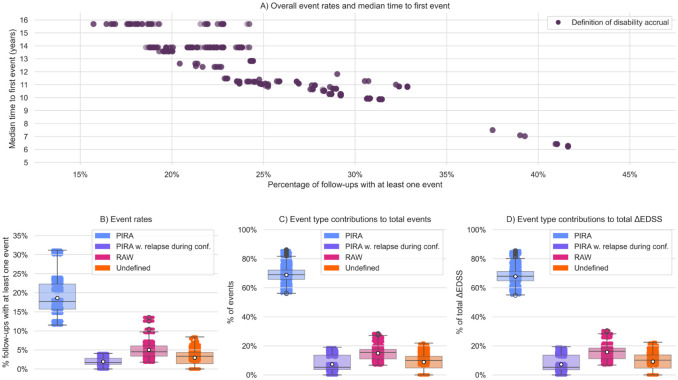
Variability of disability accrual across 1440 definitions. Panel A: Overall event rates (fraction of follow-ups with at least one disability accrual event irrespective of type) versus median time to first event (in years) for 1440 definitions of disability accrual. Each dot represents a definition. Panel B: Event rates (fraction of follow-ups with at least one disability accrual event) according to type of disability accrual. Panel C: Event type contribution (number of events of a given type of disability accrual over total number of events). Panel D: Event type contribution to the total ΔEDSS.

The proportion of follow-up periods with at least one disability accrual event irrespective of type (overall event rate) varied substantially across the 1440 tested definitions, ranging from 15.7% to 41.6% (mean 25.6%). Among follow-up periods with events, on average 74.3% had one, 19.1% two, and 6.6% had three or more events (range 1–7). Median time to first event ranged from 6.2 to 15.7 years across different event definitions (Figure 2A, eTable 1).

The proportion of follow-up periods with at least one PIRA event (PIRA event rate) ranged from 11.5% to 31.2% (mean 18.6%). PIRA events with a relapse during the confirmation period occurred in 0% to 4.1% of follow-ups (mean 2.0%). The RAW event rate ranged from 1.8% to 13.5% (mean 5.0%). Undefined disability worsening events were observed in 0% to 8.4% of follow-ups (mean 3.0%) (Figure 2B, eTable 1).

PIRA was the only event type in 10.4% to 27.8% (mean 16.6%) of follow-ups, while RAW was the only event type in 1.2% to 9.1% (mean 3.4%) (eTable 3). Neither event rates by type nor event type contributions changed significantly when merging events (eTable 4).

In the subgroup of 1361 follow-ups with ⩾ 1 relapse, overall event rates ranged from 17.3% to 59.7% (mean 34.6%). PIRA events were found in 6.5% to 32.6% of follow-ups (mean 16.6%) with RAW events ranging from 4.6% to 35.0% (mean 13.0%) (eTable 21).

### Contribution to total event count and ΔEDSS

Depending on the applied definition, 697 to 2352 disability events of any type were recorded (median: 1133), corresponding to cumulative ΔEDSS of 833.0 to 3032.5 points (median: 1385.25). Of all disability accrual events, 56.1% to 86.3% (mean 68.9%) were classified as PIRA, 0% to 19.1% (mean 7.3%) as PIRA with relapse during confirmation, 6.7% to 28.6% (mean 15.0%) as RAW, and 0% to 21.9% (mean 8.8%) as undefined (Figure 2C, eTable 2).

In follow-up periods with events, 55.1% to 82.9% (mean 65.0%) had PIRA only and 5.9% to 27.4% (mean 13.0%) RAW only (eTable 3). The proportion of total ΔEDSS attributed to PIRA ranged from 54.9% to 85.6% (mean 67.8%), to PIRA with relapse during confirmation from 0.0% to 19.4% (mean 7.3%), to RAW from 6.8% to 30.5% (mean 15.7%), and to undefined events from 0% to 22.5% (mean 9.2%) (Figure 2D, eTable 2).

In the subgroup with ⩾1 relapse, PIRA still accounted for a larger proportion of disability accrual than RAW (20.5% to 70.8% [mean 42.6%] versus 12.3% to 52.6% [mean 27.7%]; eTable 22).

### Impact of individual definition aspects

**Figure 3.**
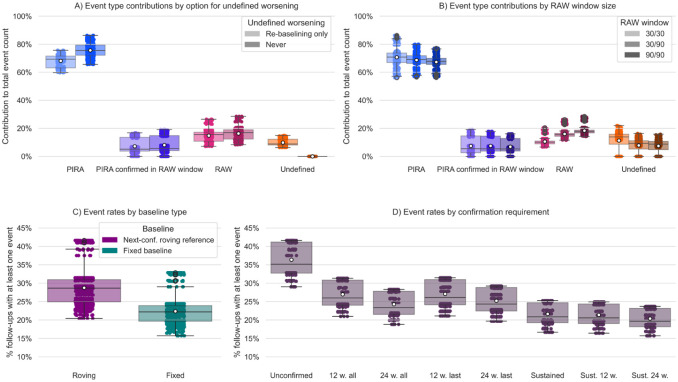
Impact of underreported or underestimated aspects of disability accrual definition. Panel A: Event type contributions (number of events of a given type over total number of events) by undefined worsening option (i.e. when a post-relapse re-baselining assessment qualifies as worsening), “re-baselining only” (i.e. when only re-baselining assessments can be undefined worsening) and “never” options only (i.e. scores at post-relapse re-baselining assessments are not checked for worsening). Panel B: Event type contributions by RAW window size. Panel C: Overall event rates (fraction of follow-ups with at least one progression event irrespective of type) by baseline option (fixed vs roving). Panel D: Overall event rates by confirmation requirement. Abbreviations: PIRA: Progression Independent of Relapse Activity. RAW: Relapse Associated Worsening. Next-conf.: next confirmed, Sust.: sustained, w.: weeks.

Omitting events identified at post-relapse re-baselining assessments significantly increased the relative contributions of PIRA and RAW to the total event count, compared to the default re-baselining only mode (from an average 68.1% to 75.6% for PIRA, and from 14.8% to 16.4% for RAW, *p* < 0.001, respectively) (Figure 3A, eTable 7).

Expanding the RAW window from 30 days pre- and post-relapse (30/30) to 30/90 or 90/90 reduced the average PIRA contribution from 70.7% to 68.7% and 67.3% (*p* < 0.001, respectively), while increasing the average RAW contribution from 10.6% to 16.0% and 18.3% (*p* < 0.001, respectively) (Figure 3B, eTable 10).

Using a roving reference confirmed at the next assessment instead of a fixed baseline increased average overall event rates from 22.4% to 28.8% (*p* < 0.001) (Figure 3C, eTable 11).

Under different confirmation requirements mean overall event rates ranged from 20.4% for sustained over ⩾ 24 weeks to 36.4% for unconfirmed disability accrual (Figure 3D).

### Sensitivity analyses

Sensitivity analysis for DMT at baseline yielded slight variations in the mean event type contributions (PIRA: 66.7% to 70.0%, RAW: 14.2% to 16.9%). The deltas between minimal and maximal PIRA and RAW contributions ranged from 27.9% to 31.9% (mean 30.2%) and from 20.2% to 22.6% (mean 21.9%), respectively (eTable 20, eFigure 16).

### Assessing “harmonized” and “standardized” definitions of PIRA

**Figure 4.**
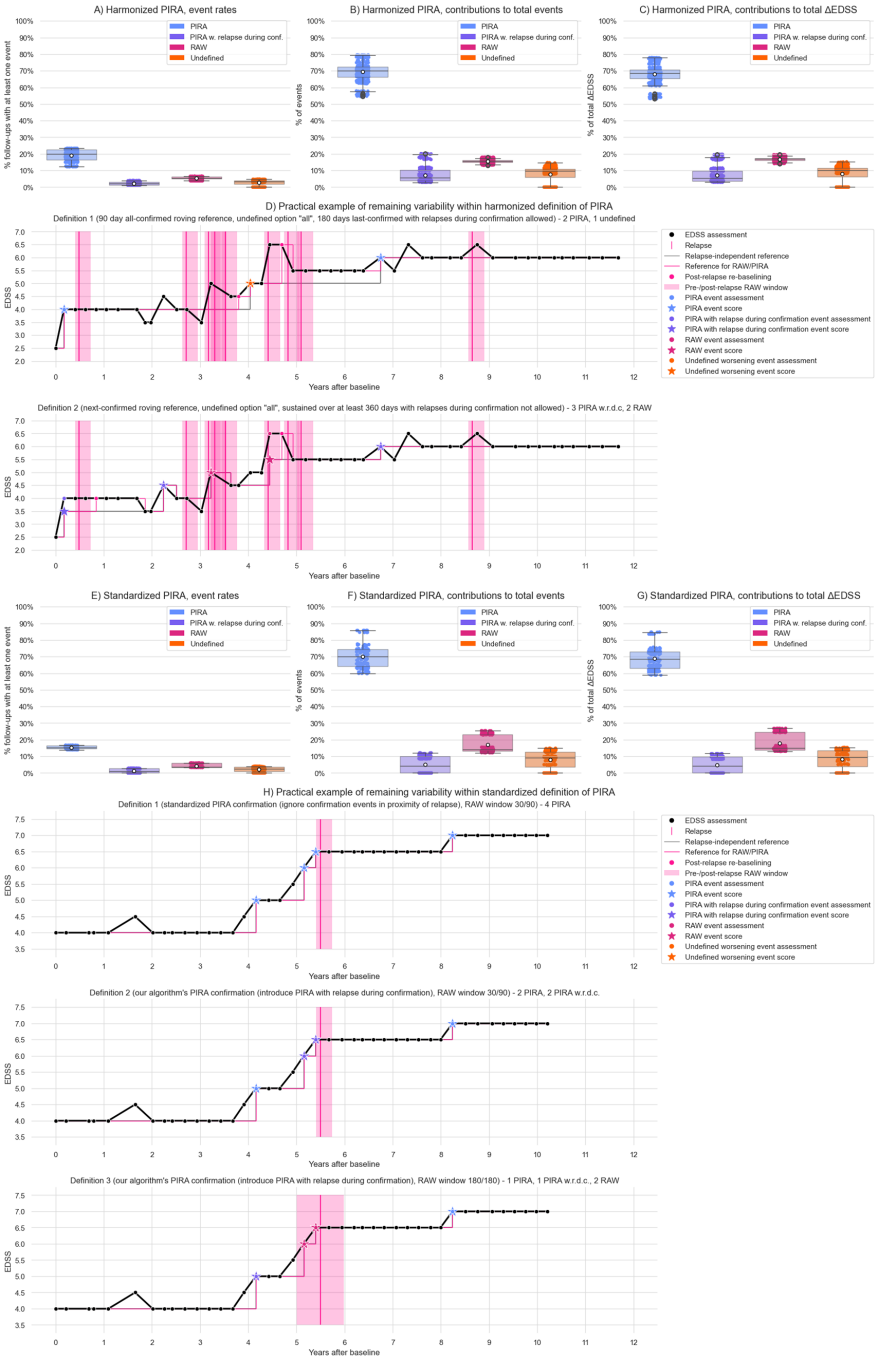
Remaining variability within proposed harmonized and standardized definitions. Panel A–C: Variability of event rates (fraction of follow-ups with at least one progression event of a given type, A), event type contributions (number of events of a given type over total number of events, B), and contributions to the total ΔEDSS (C) between the 360 options remaining within the harmonized PIRA definition.^7^ Panel D: Practical Example of remaining variability within the harmonized definition of PIRA: Definition 1 (90 days all-confirmed roving reference, 180 days last-confirmed with relapses between event and confirmation allowed for PIRA) yields 2 PIRA and 1 undefined event versus Definition 2 (Next-confirmed roving reference, sustained over at least 360 days, relapses during confirmation of PIRA not allowed) yields 3 PIRA with relapse during confirmation events and 2 RAW events. Panel E–G: Variability of event rates (E), event type contributions (F), and contributions to total ΔEDSS (G) between the 144 options remaining within the standardized PIRA definition.^9^ Panel H: Practical Example of remaining variability within the standardized definition of PIRA: Definition 1 (Standardized PIRA confirmation implementation, that is, ignore confirmation events in proximity of relapse, RAW window 30 days pre- and 90 days post-relapse) yields 4 PIRA events versus Definition 2 (Introduction of PIRA with relapse during confirmation, RAW window 30 days pre- and 90 days post-relapse) yields 2 PIRA events and 2 PIRA with relapse during confirmation events versus Definition 3 (PIRA with relapse during confirmation, RAW window 180 days pre- and 180 days post-relapse) yields 1 PIRA event, 1 PIRA with relapse during confirmation event and 2 RAW events. Abbreviations: PIRA: Progression Independent of Relapse Activity. RAW: Relapse Associated Worsening. w.r.d.c.: with relapse during confirmation.

Across the 360 combinations remaining within the harmonized PIRA definition, overall event rates varied between 18.4% and 31.2% (mean 26.0%) (eTable 27). PIRA rates ranged from 12.3% to 23.4% (mean 19.1%), with PIRA contribution to total event rate ranging from 54.5% to 79.5% (mean 69.4%). ΔEDSS ranged from 53.2% to 77.9% (mean 68.1%) (Figure 4A–C, eTable 28).

Consistently, excluding events occurring at post-relapse re-baselining assessments (as recommended by the harmonized PIRA definition) significantly increased the contributions of PIRA and RAW to the total event count compared to the default re-baselining-only approach (from an average of 68.7% to 75.2% for PIRA, and from 15.5% to 16.9% for RAW; *p* < 0.001). (eTable 31). An example of a follow-up that has 2 PIRA and 1 undefined event under one definition compatible with harmonized PIRA and 3 PIRA with relapse during confirmation and 2 RAW events under another is shown in Figure 4D.

Across the 144 parameter combinations remaining within the standardized PIRA, overall event rates ranged from 19.1% to 21.7% (mean 20.9%) (eTable 39). PIRA rates ranged from 13.9% to 16.7% (mean 15.3%), while RAW rates were 3.3% to 5.9% (mean 4.3%) (Figure 4E, eTable 40). PIRA contribution to total event rate was 59.8% to 85.8% (mean 70.1%) with RAW ranging from 12.1% to 25.4% (mean 17.0%) (Figure 4F, eTable 40). Of total ΔEDSS, attribution to PIRA ranged from 58.9% to 84.7% (mean 68.9%) and to RAW from 13.0% to 26.9% (mean 18.0%) (Figure 4G, eTable 40).

The PIRA contribution was significantly higher when applying the confirmation method of the standardized PIRA definition (discarding confirmation scores in proximity to relapses) compared to our approach (75.1% vs 65.2%; *p* < 0.001) (eTable 41).

Excluding events at post-relapse re-baselining assessments (as recommended by the standardized PIRA definition) significantly increased PIRA contribution to total event count compared to the default re-baselining-only approach (76.2% vs 69.9%, *p* < 0.001, eTable 44). Differences in event rates and event type contributions between the two implementations of roving reference confirmation were minor and not statistically significant (eTable 47). An example of a follow-up that has four PIRA events under one definition compatible with standardized PIRA and 1 PIRA, 1 PIRA with relapse during confirmation, and two RAW events under another is shown in Figure 4H.

A detailed description of each option—including those remaining within the harmonized and the standardized PIRA definitions—and their impact on event rates and event type contributions is provided in the Supplemental Materials.

## Discussion

Variability in definitions of disability accrual and the classification of PIRA versus RAW poses substantial challenges for clinical MS research, especially in real-world observational studies that often draw on heterogeneous data sources with inconsistent follow-up and incomplete records.

In this study, we aimed to quantify the frequency of PIRA and RAW events in a large, representative real-world cohort, assessing how different definitional approaches influence event rates, interrelationship, and the proportion of unclassifiable events, and evaluated recent efforts to harmonize and standardize PIRA definitions.

Across the 1440 definitional permutations we examined, overall disability accrual event rates varied widely (15.7%–41.6%) with different contributions of PIRA (56.1%–86.3% of events; 54.9%–85.6% of EDSS increase) and RAW (6.7%–28.6% of events; 6.8%–30.5% of EDSS increase). Ambiguously classified events—arising from either missing post-relapse re-baselining or occurrence of a relapse during the confirmation period—were present in 0.0% to 8.4% and 0.0% to 4.1% of all follow-ups, respectively, depending on the definition. Even when applying the recently proposed “standardized” PIRA definition, 144 distinct combinations remained, yielding event rates of 19.1%–21.7% and PIRA proportions of 59.8%–85.8% of events (58.9%–84.7% of EDSS increase).

Our findings confirm that definitional choices not only modulate the overall frequency of disability accrual events—consistent with prior reports—but also critically influence attribution to relapse-dependent versus relapse-independent mechanisms.^7–10^ PIRA consistently represented the majority of disability accrual events (at least 55% of events and ΔEDSS) across all definitions.

However, recent evidence indicates that PIRA and RAW in their current definitions do not reliably distinguish between disability worsening due to focal inflammatory disease activity and neurodegeneration.^10–12^ In line with that, an average of 5% of all events in our cohort were classified as PIRA with relapse during confirmation, reflecting the overlapping inflammatory and neurodegenerative pathology in MS.^13,14^ The co-occurrence of PIRA and relapses as well as the considerable gray in their distinguishment outlined in our study supports a paradigm shift away from a binary relapse-driven versus progressive model toward an integrated framework of disability evolution.^15^ Thus, the definition of PIRA needs further refinement incorporating imaging, for example, progression independent of relapse and MRI activity (PIRMA) or “true” PIRA, or potentially even body fluid biomarkers to reflect underlying pathophysiology.^10–12,16^ While such more expansive definitions are hardly feasible in real-world studies, the exclusion of PIRA when occurring close to RAW completely disregards the possibility that RAW and PIRA may coexist as intertwined processes.

Methodologically, our study highlights five key parameters that drive classification variability: accounting for undefined events; RAW window size; choice of fixed versus roving baselines; confirmation timing and conditions; and handling of relapses during the confirmation window. Each parameter significantly altered event rates or mechanistic attribution of disability accrual events emphasizing the need for explicit reporting of all definitional decisions in future studies, particularly those evaluating DMT effects.

The clinical implications of these definitional inconsistencies are substantial. In practice, different approaches to classifying disability accrual may alter how neurologists judge whether a patient is experiencing disease progression, with direct consequences for monitoring strategies, treatment escalation, and counseling. A patient classified as showing PIRA under one definition might be considered stable or fluctuating under another, potentially delaying therapeutic adjustments. Similarly, variation in how events are defined can complicate the interpretation of real-world evidence, particularly when comparing outcomes across centers, registries, or trials. Harmonization of definitions is therefore not only a methodological priority but also essential for ensuring consistency in patient care and treatment decisions.

Despite recent harmonization/standardization efforts, substantial ambiguity remains. Within the harmonized PIRA framework alone, 360 parameter combinations produced event rates from 18% to 31% with PIRA proportions between 55% and 79%. Even under the more rigorous “standardized” PIRA criteria, a 9% relative difference in event rate remained, with PIRA’s share of events spanning 60%–86%. Critical elements—confirmation intervals, confirmation of roving baselines, relapse handling during confirmation, “undefined” event categorization, and RAW window size—remain variably specified, allowing either under- or overestimation of progression burden. Accordingly, further refinement of a comprehensive disability accrual classification framework is imperative.

To address real-world complexities, we introduced two supplementary categories: PIRA with relapse during confirmation and undefined worsening. These were present in on average 2.0% and 3.0% of all follow-up periods and accounted for an average 7.3% and 8.8% of the total ΔEDSS, reflecting cases otherwise misclassified or omitted. Our algorithm’s ability to identify these events in real-world data from a multi-center registry underscores both the robustness of the data set as well as the necessity for transparent, consistently applied event definitions.

Recognizing that definitional choices directly affect/impact clinical and therapeutic assessment, researchers must balance sensitivity against specificity according to study aims. To facilitate this, we have made our algorithm openly accessible under the MIT license (https://github.com/drstrupf/multiple-sclerosis-disability-progression), enabling replication, adaptation, and standardized application across the research community. Moreover, our study provides the means for sample-size and power calculations for all 1440 definitional scenarios, streamlining study design despite heterogeneity in progression criteria.

Our study’s strengths include its systematic methodological approach and the use of high-quality data drawn from multiple specialized MS centers across Austria. Nonetheless, several limitations must be acknowledged. First, our retrospective analysis is based on registry data with potential documentation bias, though, the AMSTR’s standardized data collection encompassing rigorous quality-control procedures and EDSS scoring performed by certified raters mitigated this. Second, the deliberate use of real-world data—characterized by heterogeneous sources and follow-up schedules—was intended to reflect the practical challenges of applying varying definitions of disability accrual in observational cohorts and clinical practice. Third, disability quantification relied exclusively on the EDSS, an instrument less sensitive to subtle disability changes than other measures such as the timed 25-foot walk test, 9-hole peg test, and symbol digit modalities test, and disproportionately weighted toward ambulatory function, potentially overlooking PIRA in non-ambulatory domains.^17–19^ However, definitions incorporating alternative or additional measures would face similar—and potentially even greater—methodological issues in classifying disability accrual.^20^ Finally, although improved relapse control under modern DMTs can theoretically inflate estimates of PIRA risk, our sensitivity analyses did not reveal a significant effect of treatment on the variability of event type contributions between definitions.^21^

In conclusion, our study demonstrates that definitional choices critically impact disability accrual in RMS. While PIRA consistently dominates, its contribution varies substantially across definitions—even when applying recently proposed harmonized or standardizing definitions. Transparent reporting and further optimizing definitions of PIRA and RAW will be essential for improving the interpretability and comparability of future observational studies and clinical trials, as well as for guiding clinical decision-making in the management of MS.

## Supplemental Material

sj-docx-1-msj-10.1177_13524585251396283 – Supplemental material for Dissecting definitions of disability accrual in relapsing multiple sclerosis—Have we reached standardization yet? Supplemental material, sj-docx-1-msj-10.1177_13524585251396283 for Dissecting definitions of disability accrual in relapsing multiple sclerosis—Have we reached standardization yet? by Gabriel Bsteh, Stefanie Marti, Helly Hammer, Nik Krajnc, Michael Guger, Franziska Di Pauli, Jörg Kraus, Christian Enzinger, Andrew Chan, Thomas Berger, Harald Hegen and Robert Hoepner in Multiple Sclerosis Journal
