# Supplementary material for: Dissecting definitions of disability accrual in relapsing multiple sclerosis—Have we reached standardization yet?
Source: Mult Scler. 2025 Dec 6;32(2):179–91. doi: 10.1177/13524585251396283 (PMC12916871; doi:10.1177/13524585251396283)
Supplement: sj-docx-1-msj-10.1177_13524585251396283 – Supplemental material for Dissecting definitions of disability accrual in relapsing multiple sclerosis—Have we reached standardization yet? [file sj-docx-1-msj-10.1177_13524585251396283.docx]

**Dissecting definitions of disability accrual in relapsing multiple sclerosis – have we identified true progression yet?**

Gabriel Bsteh, Stefanie Marti, Helly Hammer, Nik Krajnc, Michael Guger, Franziska Di Pauli, Jörg Kraus, Christian Enzinger, Andrew Chan, Thomas Berger, Harald Hegen, and Robert Hoepner

**Supplemental Materials and Methods**

Table of contents

[Supplemental figures 4](#_Toc204673551)

[Re-baselining rules 4](#_Toc204673552)

[All four event types in one follow-up 4](#_Toc204673553)

[Supplemental results 5](#_Toc204673554)

[Event rate and event type contribution ranges 5](#_Toc204673555)

[Event rates, event type distributions, and contribution to disability accrual depending on definition 5](#_Toc204673556)

[PIRA and RAW as single contributors to disability accrual 5](#_Toc204673557)

[Single event vs. merged event mode 5](#_Toc204673558)

[Effect of event merging on event rates and type distributions 5](#_Toc204673559)

[Frequency of event merging 6](#_Toc204673560)

[Effect of the options for undefined worsening 7](#_Toc204673561)

[Effect on overall event rate 7](#_Toc204673562)

[Contribution of PIRA and RAW by handling of undefined events 7](#_Toc204673563)

[Thresholds 9](#_Toc204673564)

[Effect of the RAW window size 10](#_Toc204673565)

[PIRA and RAW by RAW window size 10](#_Toc204673566)

[Effect of baseline choice 11](#_Toc204673567)

[Event rates by baseline type 11](#_Toc204673568)

[Effect of confirmation condition 11](#_Toc204673569)

[Event rates by confirmation condition 11](#_Toc204673570)

[Event type distribution by confirmation condition 13](#_Toc204673571)

[Sensitivity analyses 14](#_Toc204673572)

[Supplemental subgroup analyses 16](#_Toc204673573)

[Follow-ups with at least one relapse only 16](#_Toc204673574)

[Cohort 16](#_Toc204673575)

[Event rate and event type contribution 16](#_Toc204673576)

[Impact of individual definition aspects 17](#_Toc204673577)

[Follow-ups with more conservative inclusion criteria 19](#_Toc204673578)

[Cohort 19](#_Toc204673579)

[Event rate and event type contribution 19](#_Toc204673580)

[Impact of individual definition aspects 21](#_Toc204673581)

[Analysis of the harmonized PIRA definition 22](#_Toc204673582)

[Methods 22](#_Toc204673583)

[Differences between our algorithm and the harmonized definition 22](#_Toc204673584)

[Results 23](#_Toc204673585)

[Event rate and event type contribution ranges 23](#_Toc204673586)

[Effect of the options for undefined worsening 24](#_Toc204673587)

[Effect of roving reference confirmation options 26](#_Toc204673588)

[Effect of confirmation options 27](#_Toc204673589)

[Relapses in confirmation interval 28](#_Toc204673590)

[Example of a follow-up with a large variation in event type attribution 29](#_Toc204673591)

[Analysis of the standardized PIRA definition 30](#_Toc204673592)

[Methods 30](#_Toc204673593)

[Roving reference confirmation 30](#_Toc204673594)

[Relapses prior to PIRA candidate events 31](#_Toc204673595)

[Relapses in the confirmation interval of PIRA events 32](#_Toc204673596)

[Tested options 34](#_Toc204673597)

[Results 35](#_Toc204673598)

[Event rate and event type contribution ranges 35](#_Toc204673599)

[Effect of the options for confirmation 35](#_Toc204673600)

[Effects of the RAW window size 36](#_Toc204673601)

[Effect of the options for undefined worsening 38](#_Toc204673602)

[Effect of baseline choice 40](#_Toc204673603)

[Example of a follow-up with a large variation in event type attribution 41](#_Toc204673604)

[Supplemental methods 42](#_Toc204673605)

[General remarks 42](#_Toc204673606)

[Notation 42](#_Toc204673607)

[Relapse-independent definition aspects 42](#_Toc204673608)

[Minimal required increase 42](#_Toc204673609)

[Event confirmation 43](#_Toc204673610)

[Confirmed score 43](#_Toc204673611)

[Confirmation condition 43](#_Toc204673612)

[Confirmation interval 43](#_Toc204673613)

[Confirmation distance types 44](#_Toc204673614)

[Confirmation distance tolerances and constraints 44](#_Toc204673615)

[Special options for sustained 45](#_Toc204673616)

[Optional exemption from confirmation for the last assessment 45](#_Toc204673617)

[Minimal distance 46](#_Toc204673618)

[Minimal distance to previous assessment 46](#_Toc204673619)

[Minimal distance to the reference assessment 46](#_Toc204673620)

[Minimal distance and confirmation 47](#_Toc204673621)

[Minimal distance to roving reference 47](#_Toc204673622)

[Baselines 48](#_Toc204673623)

[Baseline types 48](#_Toc204673624)

[Confirmation options for the roving reference 49](#_Toc204673625)

[Post-event re-baselining 50](#_Toc204673626)

[Post-event re-baselining and roving reference 50](#_Toc204673627)

[Post-event re-baselining, roving reference, and minimal distance to reference 50](#_Toc204673628)

[Event merging 51](#_Toc204673629)

[Maximal distance 51](#_Toc204673630)

[Maximal repetition distance 51](#_Toc204673631)

[Short-term improvements 52](#_Toc204673632)

[Short-term improvements with all vs. last confirmation 53](#_Toc204673633)

[Relapse-related definition aspects 54](#_Toc204673634)

[Event types 54](#_Toc204673635)

[RAW and PIRA 54](#_Toc204673636)

[PIRA with relapse during confirmation and Undefined Worsening 54](#_Toc204673637)

[RAW/PIRA reference and relapse-independent reference 54](#_Toc204673638)

[Relapses and event confirmation 55](#_Toc204673639)

[Relapses, event types, and event merging 56](#_Toc204673640)

[Post-relapse re-baselining 57](#_Toc204673641)

[General rules 57](#_Toc204673642)

[Post-relapse re-baselining and events 57](#_Toc204673643)

[Post-relapse re-baselining and minimal distance 57](#_Toc204673644)

[Multiple relapses 58](#_Toc204673645)

[Post-relapse re-baselining and ambiguous events 58](#_Toc204673646)

[Undefined worsening 59](#_Toc204673647)

[Options 59](#_Toc204673648)

[Which option should I use for my endpoint? 60](#_Toc204673649)

[Counterintuitive events with the “all” option 61](#_Toc204673650)

[Thresholds for undefined events 62](#_Toc204673651)

[Example of all four event types in one follow-up 64](#_Toc204673652)

[References 65](#_Toc204673653)

# Supplemental figures

## Re-baselining rules


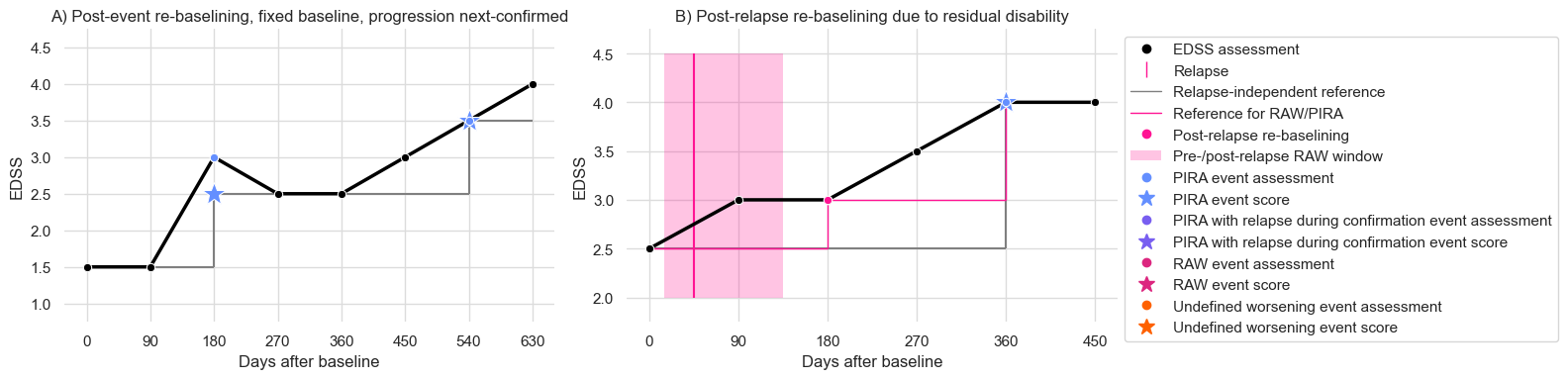


**eFigure 1**: Re-baselining rules. **A)** Post-event re-baselining. Progression must be confirmed at the next assessment in this example, thus the confirmed event score that sets the new reference is 2.0. **B)** Post-relapse re-baselining. Residual disability after the relapse, thus the reference is set to 3.0. Minimal required increase + 1.0, confirmation at the next assessment required.

## All four event types in one follow-up


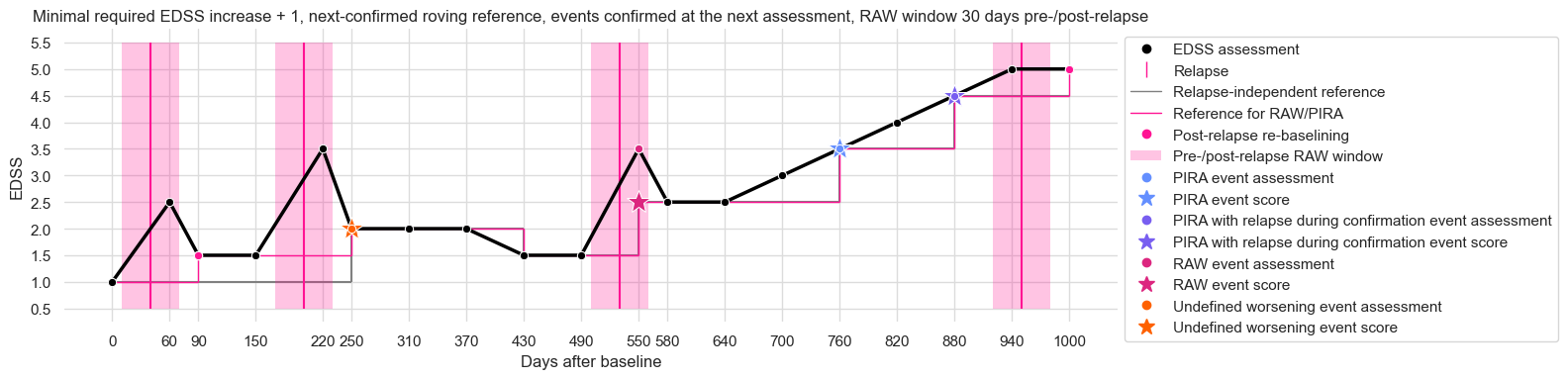


**eFigure 2**: Example of multiple events, confirmation at the next assessment required, next-confirmed roving reference, RAW window 30 days pre- and post-relapse, min. increase + 1.0. The worsening at day 60 after the first relapse is not confirmed, thus it is not classified as RAW. The residual disability measured at the post-relapse re-baselining assessment at day 90 is used as new reference, and the post-relapse re-baselining assessment at day 250 after the second relapse is a progression with respect to the initial reference but neither RAW nor PIRA, thus it is classified as undefined worsening. The roving reference is reset at day 430 (confirmed at day 490), thus the worsening at day 550 is a confirmed (at day 580) RAW event with event score 2.5. The increase at day 760 confirmed at day 820 is PIRA with respect to this new post-event reference with an event score of 3.5. The confirmation assessment for the increase at day 880 is in the RAW window of the subsequent relapse, thus the increase is classified as PIRA with relapse during confirmation.

# Supplemental results

Analysis of 3,525 follow-ups of patients with RMS for 1,440 definition of disability accrual and the classification of events into PIRA, PIRA with relapse during confirmation, RAW, and undefined worsening.

## Event rate and event type contribution ranges

### Event rates, event type distributions, and contribution to disability accrual depending on definition

The proportions of follow-ups with at least one disability accrual event irrespective of type and by type are shown in eTable 1.

|  | Event rate (%) | | | Median time to first event (years) | | |
| --- | --- | --- | --- | --- | --- | --- |
|  | Min | Mean | Max | Min | Mean | Max |
| Disability accrual (overall) | **15.7** | **25.6** | **41.6** | **6.2** | **12.1** | **15.7** |
| PIRA | 11.5 | 18.6 | 31.2 | 9.9 | 14.0 | 15.7 |
| PIRA with relapse during confirmation | 0.0 | 2.0 | 4.1 | - | - | - |
| RAW | 1.8 | 5.0 | 13.5 | - | - | - |
| Undefined | 0.0 | 3.0 | 8.4 | - | - | - |

**eTable 1**: Range of event rates (fraction of follow-ups with at least one progression event) and median time to first event (Kaplan-Meier) overall and by progression type for 1,440 definitions of disability accrual. Within the same cohort, the proportion of follow-ups with at least one event can be as low as 15.7% and as high as 41.6%, depending only on the chosen definition.

The contributions of each event type to the total number of disability accrual events and to the total EDSS increase are shown in eTable 2.

|  | Contribution to total events (%) | | | Contribution to ΔEDSS (%) | | |
| --- | --- | --- | --- | --- | --- | --- |
|  | Min | Mean | Max | Min | Mean | Max |
| PIRA | 56.1 | 68.9 | 86.3 | 54.9 | 67.8 | 85.6 |
| PIRA with relapse during confirmation | 0.0 | 7.3 | 19.1 | 0.0 | 7.3 | 19.4 |
| RAW | 6.7 | 15.0 | 28.6 | 6.8 | 15.7 | 30.5 |
| Undefined | 0.0 | 8.8 | 21.9 | 0.0 | 9.2 | 22.5 |

**eTable 2**: Range of contribution of each event type to the total number of events and to the total EDSS score increase depending on definition of disability accrual. Within the same cohort, the contribution of PIRA to total events can be as low as 56.1% and as high as 86.3%, depending only on the chosen definition.

### PIRA and RAW as single contributors to disability accrual

PIRA was the only event type in 10.4% to 27.8% (mean 16.6%) of all follow-ups, and RAW was the only disability accrual type in 1.2% to 9.1% (mean 3.4%) of all follow-ups. Of the follow-ups with events, 55.1% to 82.9% (mean 64.9%) had PIRA only, 5.9% to 27.4% (mean 13.0%) had RAW only, and 0% to 17.8% (mean 7.2%) only had undefined worsening (eTable 3).

|  | Proportion of follow-ups (%) | | | Of follow-ups with events (%) | | |
| --- | --- | --- | --- | --- | --- | --- |
|  | Min | Mean | Max | Min | Mean | Max |
| PIRA only | 10.4 | 16.6 | 27.8 | 55.1 | 65.0 | 82.9 |
| PIRA with relapse during confirmation only | 0.0 | 1.1 | 3.1 | 0.0 | 4.8 | 15.4 |
| RAW only | 1.2 | 3.4 | 9.1 | 5.9 | 13.0 | 27.4 |
| Undefined only | 0.0 | 1.8 | 4.3 | 0.0 | 7.2 | 17.8 |

**eTable 3**: Fraction of follow-ups where a given event type is the single contributor to the total disability accrual (left) and fraction of follow-ups with events where a given event type is the single contributor to the total disability accrual (right). For example, of all 3,525 follow-ups, up to 27.8% (depending on the definition) have PIRA but no worsening of any other type. Of all follow-ups with events (up to 41.6% of all follow-ups, depending on the definition), up to 82.9% only experience PIRA.

## Single event vs. merged event mode

### Effect of event merging on event rates and type distributions

The overall event rate (fraction of follow-ups with at least one event) was not affected by merging events. Overall event counts were smaller when merging events, with a mean of 1,251 events in single event mode and a mean of 1,194 events in merged mode (p < 0.001), all other definition aspects equal (720 comparisons). Changes to event rates or event contributions by event type due to merging subsequent events into one event were very small and not statistically significant (eTable 4). The rates for each event type except PIRA varied slightly since merging events could lead to higher event scores that then mask subsequent events (see Supplemental Methods).

|  | Mean event rate (%) | | | Mean event contribution (%) | | |
| --- | --- | --- | --- | --- | --- | --- |
|  | Single | Merged | p | Single | Merged | p |
| PIRA | 18.6 | 18.6 | - | 69.1 | 68.6 | 0.121 |
| PIRA with relapse during conf. | 2.0 | 1.9 | 0.906 | 7.3 | 7.2 | 0.671 |
| RAW | 5.0 | 5.0 | 0.960 | 14.8 | 15.2 | 0.084 |
| Undefined | 3.0 | 2.9 | 0.571 | 8.7 | 9.0 | 0.443 |

**eTable 4**: Event rates (left) and event type contributions (right) for single event mode vs. merged event mode, all other definition aspects equal (720 comparisons). A “-” indicates no difference.

### Frequency of event merging

The maximum number of EDSS assessments merged into one continuous event was 6 for PIRA, 3 for PIRA with relapse during confirmation, and 3 for RAW. Undefined events were never merged (by design). Even with event merging, on average 90.9% (min. 89.8%, max. 92.3%) of the PIRA events were single events, while only 9.1% were composed of more than one event. For RAW, on average 96.4% (min. 94.5%, max. 98.9%) were single events (eTable 5, eFigure 3).

|  | Mean composition of merged events (%) | | | | | |
| --- | --- | --- | --- | --- | --- | --- |
|  | One | Two | Three | Four | Five | Six |
| PIRA | 90.73 | 8.31 | 0.92 | 0.01 | 0.02 | 0.01 |
| PIRA with relapse during conf. | 93.11 | 6.22 | 0.67 | 0.00 | 0.00 | 0.00 |
| RAW | 96.35 | 3.08 | 0.57 | 0.00 | 0.00 | 0.00 |
| Undefined | 100.0 | 0.00 | 0.00 | 0.00 | 0.00 | 0.00 |

**eTable 5**: Mean composition of merged events (number of EDSS assessment), by event type, 720 definitions.


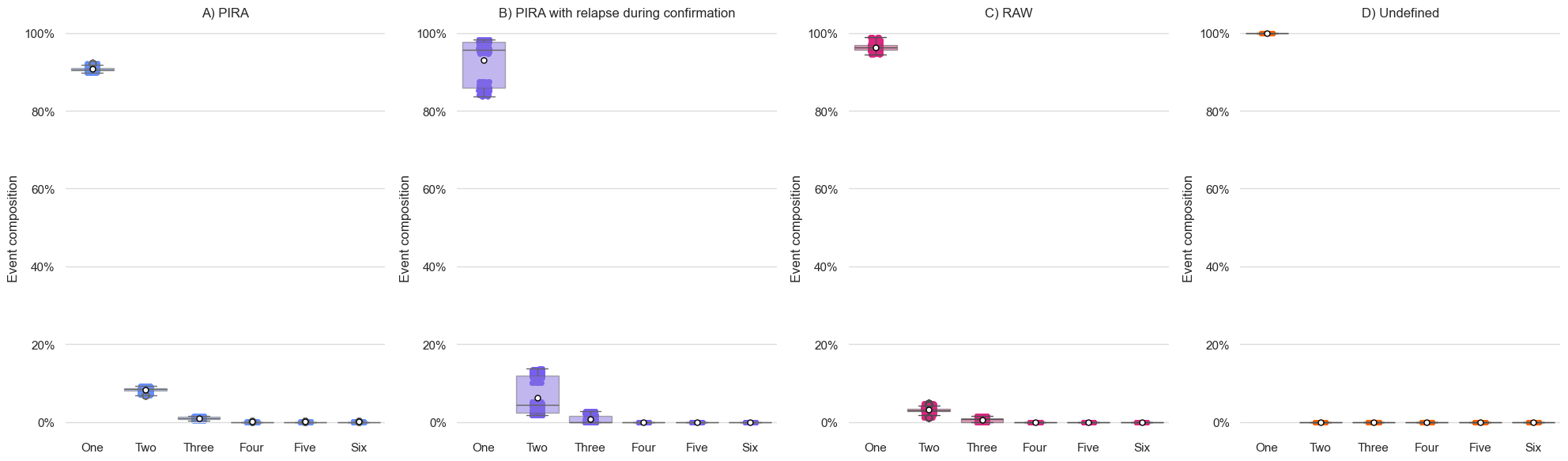


**eFigure 3**: Composition of merged events, by event type, 720 definitions. **A)** PIRA contribution. **B)** PIRA with relapse during confirmation contribution. **C)** RAW contribution. **D)** Undefined contribution.

## Effect of the options for undefined worsening

### Effect on overall event rate

Ignoring events at post-relapse re-baselining assessments significantly decreased the **overall** event rate (follow-ups with at least one event irrespective of type) from 25.8% with the “re-baselining only” option to 23.8% with the “never” option (p < 0.001), and increased the time to first event (irrespective of type) from 12.0 years with the “re-baselining only” option to 12.6 years with the “never” option (p = 0.001). The “end” and “all” options (that have equal overall event rates by design) increased the event rate from 25.8% with the “re-baselining only” option to 26.4%, but this increase was not statistically significant (p = 0.127). Event rates **by type** do not change significantly, except for undefined worsening, which was ignored with the “never” option (eTable 6).

|  | Mean event rate (%) | | | | p-value vs. RB only | | |
| --- | --- | --- | --- | --- | --- | --- | --- |
|  | RB only | Never | End | All | Never | End | All |
| Disability accrual (overall) | **25.8** | **23.8** | **26.4** | **24.4** | **<0.001** | **0.127** | **0.127** |
| PIRA | 18.6 | 18.6 | 18.6 | 18.5 | - | - | 0.774 |
| PIRA with relapse during conf. | 2.0 | 2.0 | 2.0 | 1.9 | - | - | 0.727 |
| RAW | 5.0 | 5.0 | 5.0 | 5.0 | - | - | 0.785 |
| Undefined | 3.3 | 0.0 | 3.3 | 4.5 | <0.001 | <0.001 | <0.001 |

**eTable 6**: Mean event rates (proportion of follow-ups with at least one event) for the four options for undefined worsening, and p-values (permutation test) for the comparison of the “never”, “all”, and “end” options to the default “re-baselining only” option (360 comparisons each). **Abbreviations**: RB only: re-baselining only. A “-” indicates no difference.

### Contribution of PIRA and RAW by handling of undefined events

Ignoring events at post-relapse re-baselining assessments significantly increased PIRA and RAW contributions to the total event count compared to the default re-baselining only mode where post-relapse re-baselining assessments can be disability accrual events (eFigure 4, eTable 7).


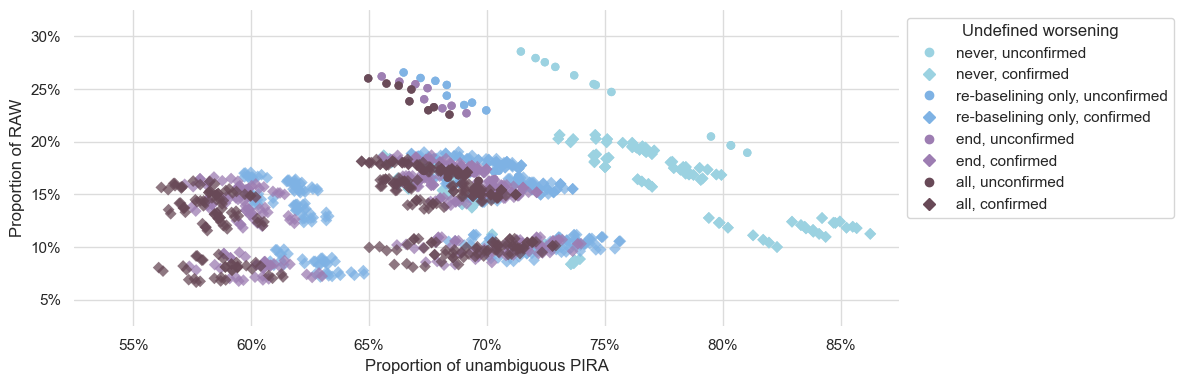


**eFigure 4**: Contribution of PIRA (x-axis) and RAW (y-axis) to the total number of events, 1,440 definitions. The options for undefined worsening are color coded, and definitions where any type of confirmation is required are shown as diamonds while definitions with no confirmation requirements are shown as circles. Definitions with “never” for undefined worsening and no confirmation requirement only have events of the types “PIRA” or “RAW”.

|  | Mean event contribution (%) | | | | p-value vs. RB only | | |
| --- | --- | --- | --- | --- | --- | --- | --- |
|  | RB only | Never | End | All | Never | End | All |
| PIRA | 68.1 | 75.6 | 66.4 | 65.4 | <0.001 | <0.001 | <0.001 |
| PIRA with relapse during conf. | 7.2 | 8.1 | 7.0 | 6.9 | 0.047 | 0.581 | 0.321 |
| RAW | 14.8 | 16.4 | 14.5 | 14.2 | <0.001 | 0.267 | 0.052 |
| Undefined | 9.8 | 0.0 | 12.1 | 13.5 | <0.001 | <0.001 | <0.001 |

**eTable 7**: Event type contributions by undefined worsening annotation mode, 360 definitions per mode. **Abbreviations**: RB only: re-baselining only.

The mean contribution of **PIRA** to the total event count increased from 68.1% to 75.6% (p < 0.001) when ignoring all undefined events instead of allowing undefined events at re-baselining assessments (all other definition aspects equal, i.e. 360 comparisons) (eTable 7, eFigure 5A).

The mean contribution of **RAW** to the total event count increased from 14.8% to 16.4% (p < 0.001) when ignoring all undefined events instead of allowing undefined events at re-baselining assessments (all other definition aspects equal, i.e. 360 comparisons) (eTable 7, eFigure 5C).


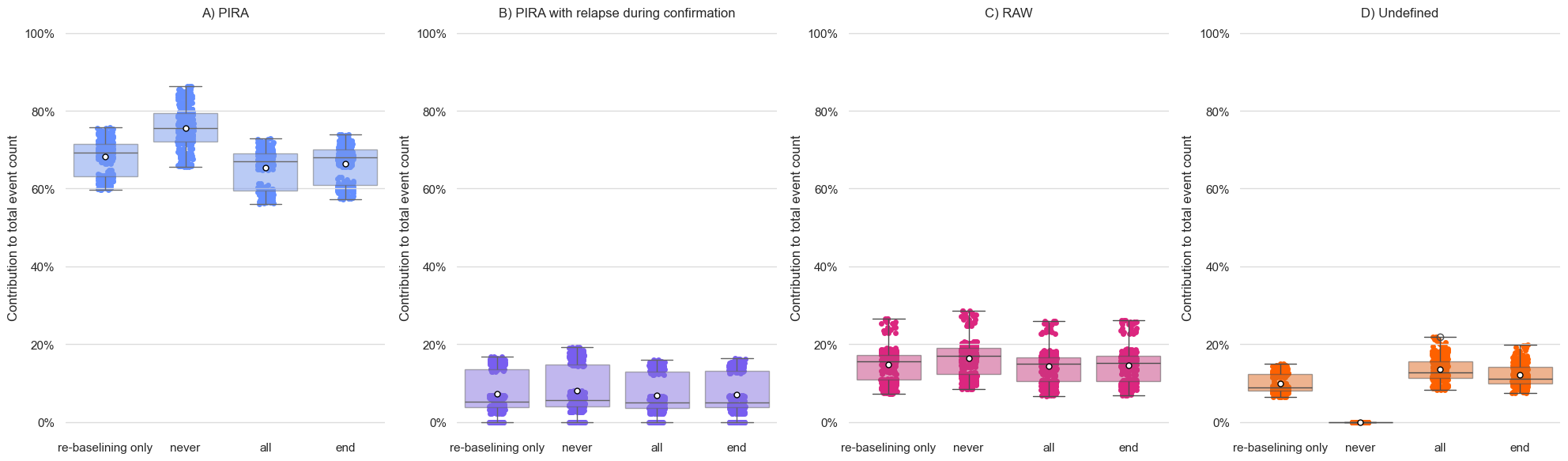


**eFigure 5**: Contributions of each event type to the overall event count by option for undefined worsening, 360 definitions each. **A)** PIRA contribution. **B)** PIRA with relapse during confirmation contribution. **C)** RAW contribution. **D)** Undefined contribution.

The subgroups visible in eFigure 5 B), C), and D) were due to confirmation conditions and RAW window sizes, see eFigures 6, 7, 8.


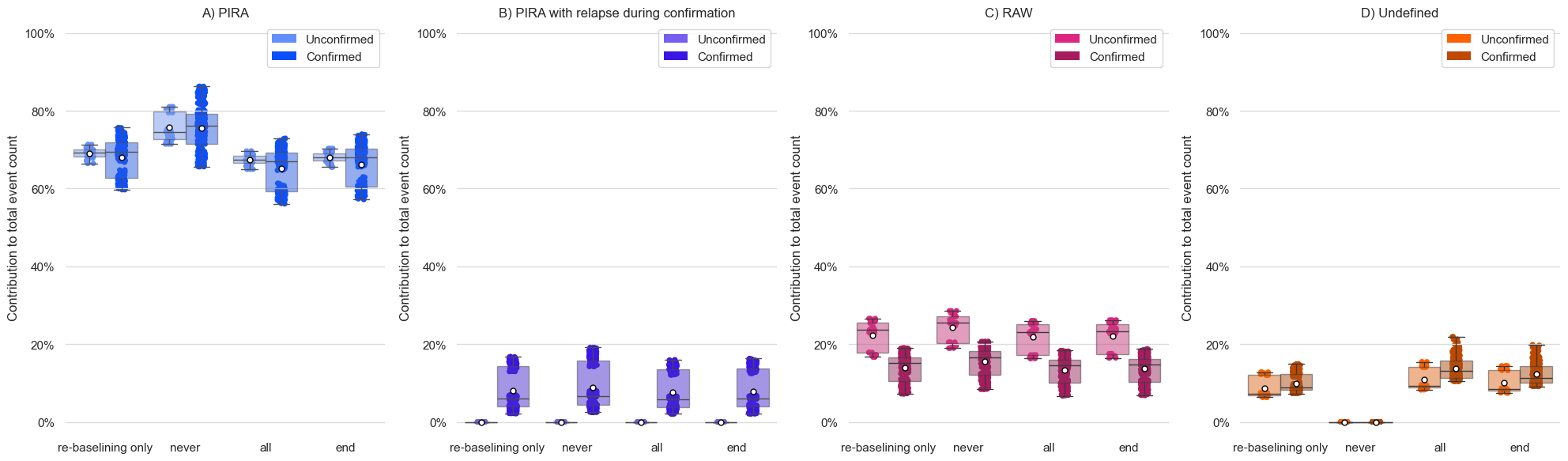


**eFigure 6**: Contributions of each event type to the overall event count, by absence (36 definitions each) or presence (324 definitions each) of a confirmation requirement. **A)** PIRA contribution. **B)** PIRA with relapse during confirmation contribution. **C)** RAW contribution. **D)** Undefined contribution.


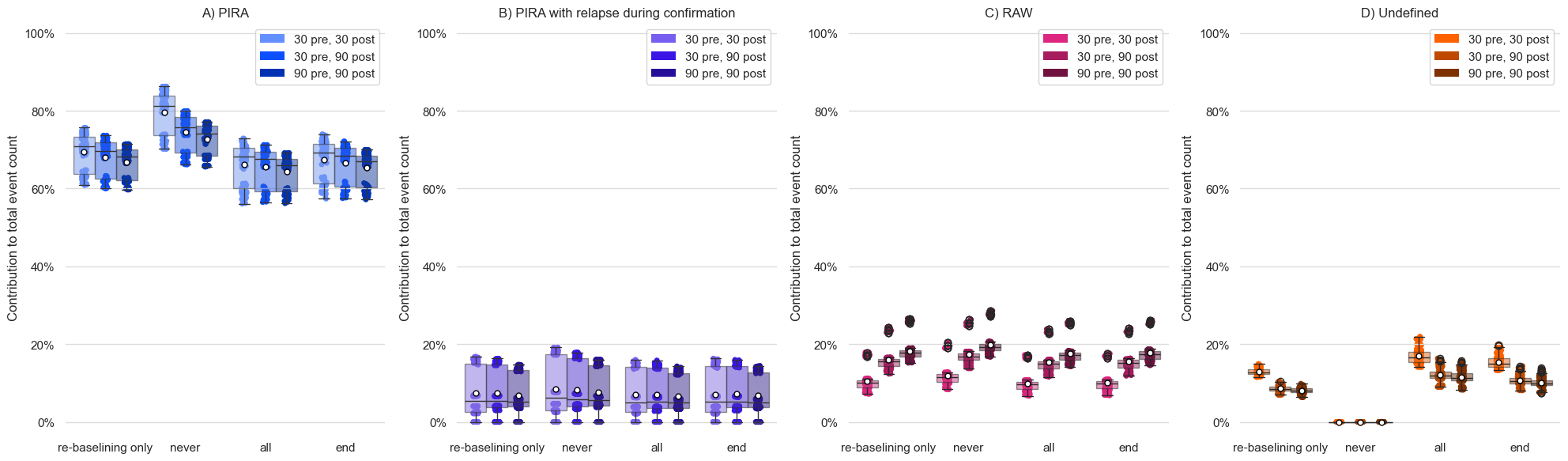


**eFigure 7**: Contributions of each event type to the overall event count, by RAW window size in days pre- and post-relapse (120 definitions per box). **A)** PIRA contribution. **B)** PIRA with relapse during confirmation contribution. **C)** RAW contribution. **D)** Undefined contribution.


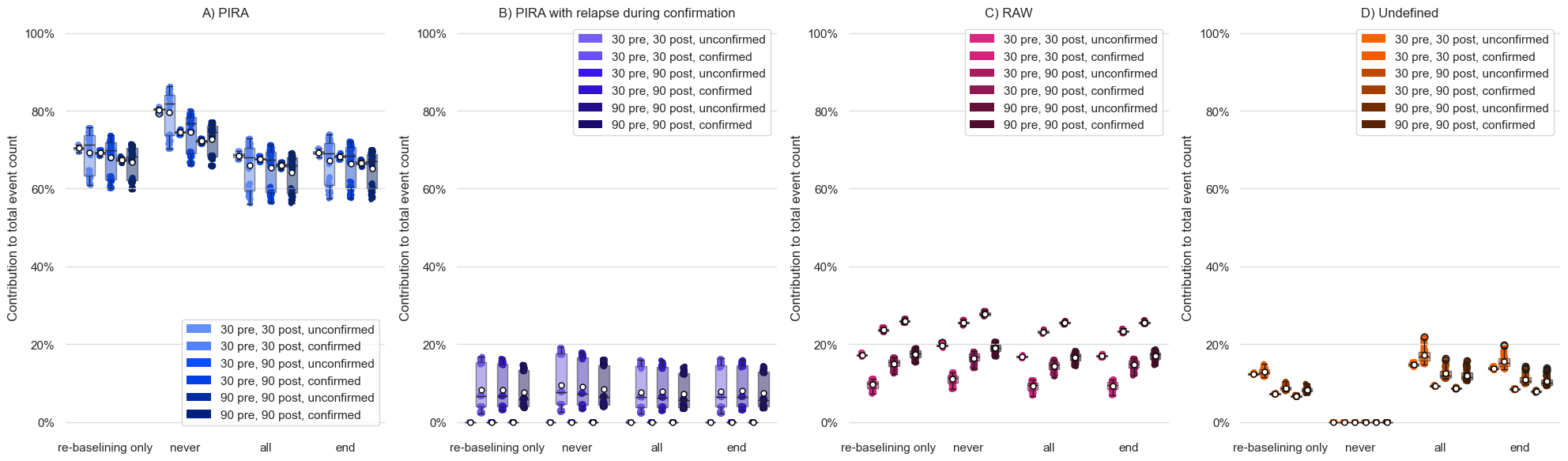


**eFigure 8**: Contributions of each event type to the overall event count, by RAW window size in days pre- and post-relapse and confirmation requirement. Boxes with undefined worsening contain 12 definitions; boxes with confirmation requirement contain 108 definitions. **A)** PIRA contribution. **B)** PIRA with relapse during confirmation contribution. **C)** RAW contribution. **D)** Undefined contribution.

### Thresholds

Requiring a potential undefined event score to have a greater or equal or a greater score than the current RAW/PIRA baseline did not have a large impact on event rates or event type contributions. The mean overall event rates are 26.2%, 26.2%, and 26.1% for the “any”, “equal or greater”, and “greater only” options, respectively (the “never” option for undefined worsening excluded because only undefined events are affected by this threshold choice). The proportion of follow-ups with at least one undefined event decreased from 4.0% with the “any” option to 3.8% with the “greater only” option (p = 0.007, 360 comparisons, “never” for undefined excluded), while the rates for the other event types remained unchanged (eTable 8).

|  | Mean event rate (%) | | | p-value vs. “any” | |
| --- | --- | --- | --- | --- | --- |
|  | Any | Equal or greater | Greater only | Equal or greater | Greater only |
| PIRA | 18.6 | 18.6 | 18.6 | - | 0.996 |
| PIRA with relapse during conf. | 2.0 | 2.0 | 2.0 | - | - |
| RAW | 5.0 | 5.0 | 5.0 | - | - |
| Undefined | 4.0 | 4.0 | 3.8 | 0.673 | 0.007 |

**eTable 8**: Event rates by undefined worsening threshold mode, 360 definitions per mode. A “-” indicates no difference.

Changes to the event type distribution were not statistically significant except for undefined worsening (eTable 9).

|  | Mean event contribution (%) | | | p-value vs. “any” | |
| --- | --- | --- | --- | --- | --- |
|  | Any | Equal or greater | Greater only | Equal or greater | Greater only |
| PIRA | 66.4 | 66.5 | 67.0 | 0.689 | 0.063 |
| PIRA with relapse during conf. | 7.0 | 7.0 | 7.1 | 0.938 | 0.766 |
| RAW | 14.5 | 14.5 | 14.6 | 0.945 | 0.689 |
| Undefined | 12.2 | 12.0 | 11.3 | 0.409 | <0.001 |

**eTable 9**: Event type contributions, 360 definitions per mode. A “-” indicates no difference.

## Effect of the RAW window size

### PIRA and RAW by RAW window size

Contributions of RAW and PIRA to the total number of disability accrual events ranged from less than 7% and 57%, respectively, to more than 20% and 86%, respectively, depending on the definition (eFigure 9).


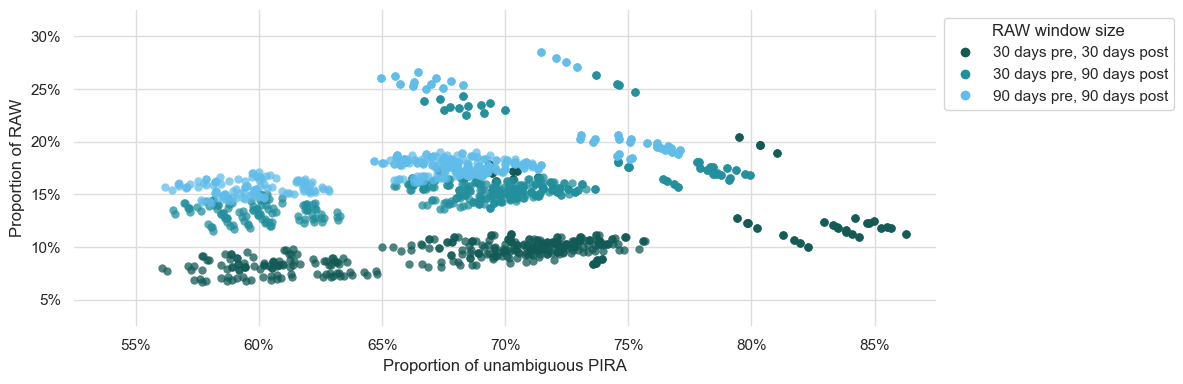


**eFigure 9**: Contribution of PIRA (x-axis, unambiguous only, i.e. no PIRA confirmed in RAW window) and RAW (y-axis) to the total number of events, 1,440 definitions. The RAW window sizes are color-coded. Note that this plot shows the same data as eFigure 4, but with different color-coding to highlight RAW window sizes instead of undefined worsening options.

The contribution of **PIRA** to the total event count ranged from 56.1% to 86.3% (mean 70.7%) for a RAW window of 30 days pre- and post-relapse, from 56.5% to 79.9% (mean 68.7%) for a RAW window of 30 days pre- and 90 days post-relapse, and from 56.2% to 77.1% (mean 67.3%) for a RAW window of 90 days pre- and post-relapse (eFigure 10A).

The contribution of **RAW** to the total event count ranged from 6.7% to 20.5% (mean 10.6%) for a RAW window of 30 days pre- and post-relapse, from 11.6% to 26.3% (mean 16.0%) for a RAW window of 30 days pre- and 90 days post-relapse, and from 14.2% to 28.6% (mean 18.3%) for a RAW window of 90 days pre- and post-relapse (eFigure 10C).


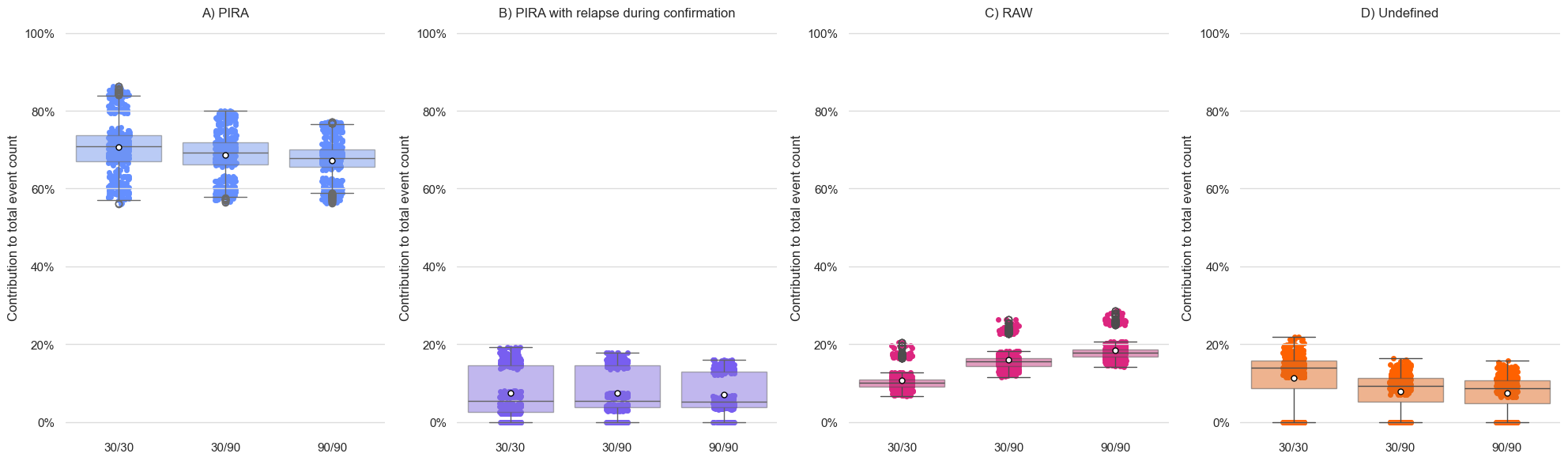


**eFigure 10**: Contributions of each event type to the overall event count by option for the RAW window size, 480 definitions each. **A)** PIRA contribution. **B)** PIRA with relapse during confirmation contribution. **C)** RAW contribution. **D)** Undefined contribution.

|  | Mean event contribution (%) | | | p-value when compared to 30/30 | |
| --- | --- | --- | --- | --- | --- |
|  | 30/30 | 30/90 | 90/90 | 30/90 | 90/90 |
| PIRA | 70.7 | 68.7 | 67.3 | <0.001 | <0.002 |
| PIRA with relapse during conf. | 7.5 | 7.5 | 6.9 | 0.971 | 0.131 |
| RAW | 10.6 | 16.0 | 18.3 | <0.001 | <0.001 |
| Undefined | 11.3 | 7.8 | 7.4 | <0.001 | <0.001 |

**eTable 10**: Event type contributions by RAW window size, 480 definitions per window size. P-values are shown for the comparison with the 30/30 window. 480 comparisons (all other definition aspects equal), permutation test.

The average contribution of **PIRA** to the total event count increased from 67.3% to 70.7% (p < 0.001) when decreasing the RAW window from 90/90 to 30/30 (all other definition aspects the same, i.e. 480 comparisons) (eTable 10).

The average contribution of **RAW** to the total event count decreased from 18.3% to 10.6% (p < 0.001) when decreasing the RAW window from 90/90 to 30/30 (all other definition aspects the same, i.e. 480 comparisons) (eTable 10).

## Effect of baseline choice

### Event rates by baseline type

The average fraction of follow-ups with at least one disability accrual **event of any type** increased from 22.4% to 28.8% (p < 0.001) when using a next-confirmed roving reference instead of the fixed study baseline (all other definition aspects equal, i.e. 720 comparisons). The average fraction of follow-ups with at least one **PIRA** event increased from 16.2% to 21.1% (p < 0.001) when using a next-confirmed roving reference instead of the fixed study baseline (all other definition aspects equal, i.e. 720 comparisons). The average fraction of follow-ups with at least one **RAW** event increased from 4.3% to 5.8% (p < 0.001) when using a next-confirmed roving reference instead of the fixed study baseline (all other definition aspects equal, i.e. 720 comparisons). There were no relevant differences in the event type contributions (eTable 11, eFigure 11).

|  | Mean event rate (%) | | | Mean event contribution (%) | | |
| --- | --- | --- | --- | --- | --- | --- |
|  | Fixed | NC roving | p-value | Fixed | NC roving | p-value |
| Disability accrual (overall) | **22.4** | **28.8** | **<0.001** | **-** | **-** | **-** |
| PIRA | 16.2 | 21.1 | <0.001 | 68.8 | 69.0 | 0.466 |
| PIRA with relapse during conf. | 1.7 | 2.2 | <0.001 | 7.3 | 7.3 | 0.904 |
| RAW | 4.3 | 5.8 | <0.001 | 14.8 | 15.1 | 0.186 |
| Undefined | 2.6 | 3.3 | <0.001 | 9.1 | 8.6 | 0.050 |

**eTable 11**: Mean event rates (proportion of follow-ups with at least one event) and event type contributions for fixed baseline and next-confirmed roving reference, 720 comparisons (all other definition aspects equal), permutation test. Abbreviations: NC: next-confirmed.


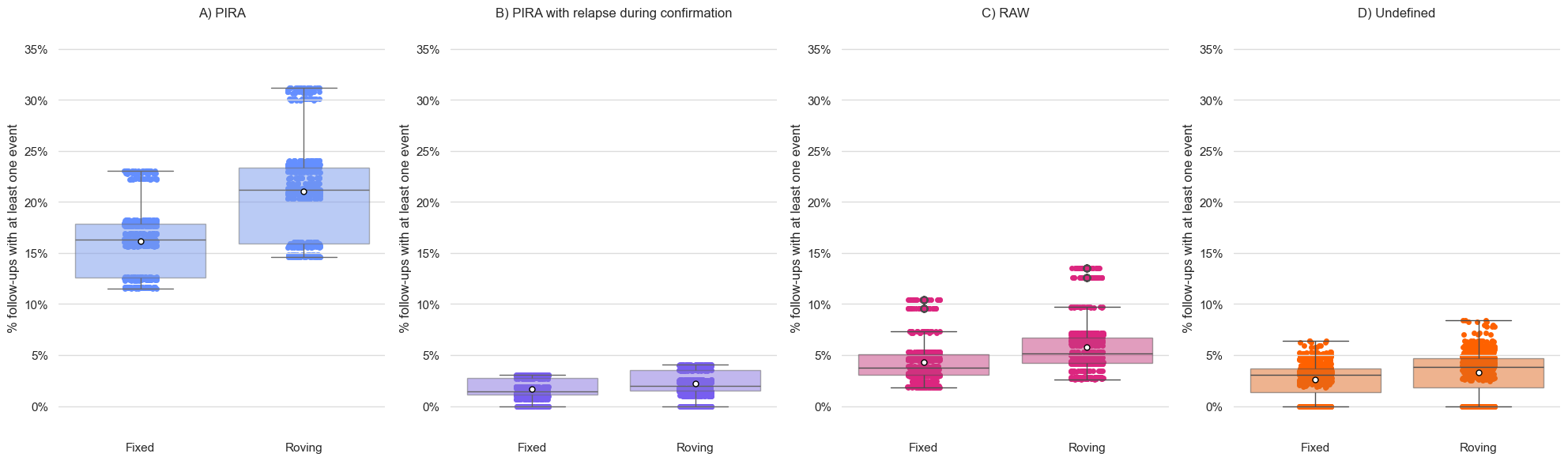


**eFigure 11**: Event rates by option for the baseline choice, 720 definitions each. **A)** PIRA. **B)** PIRA with relapse during confirmation. **C)** RAW. **D)** Undefined.

## Effect of confirmation condition

### Event rates by confirmation condition

Mean **overall event rates** ranged from 20.4% for sustained over a minimum of 24 weeks to 36.4% for unconfirmed disability accrual. The mean overall event rate for 24-weeks confirmation including all values within the confirmation interval is 24.3%. eTable 12 and eFigure 12 show overall event rates by confirmation requirement and baseline choice.

|  | Mean event rate (%) | | | | | | | |
| --- | --- | --- | --- | --- | --- | --- | --- | --- |
|  | Unconf. | 12 w. all | 24 w. all | 12 w. last | 24 w. last | Sustained | Sust. 12 w | Sust. 24 w |
| Overall | **36.4** | **27.0** | **24.3** | **27.1** | **25.2** | **21.6** | **21.4** | **20.4** |
| Fixed baseline | 32.0 | 23.5 | 21.1 | 23.6 | 22.1 | 19.0 | 18.8 | 17.9 |
| NC roving | 40.7 | 30.4 | 27.4 | 30.6 | 28.4 | 24.3 | 24.0 | 22.8 |

**eTable 12**: Mean event rates for the 8 analyzed confirmation options, overall (both baselines) and by baseline choice. **Abbreviations**: NC: Next confirmed, Sust: sustained, Unconf: unconfirmed, w: weeks.


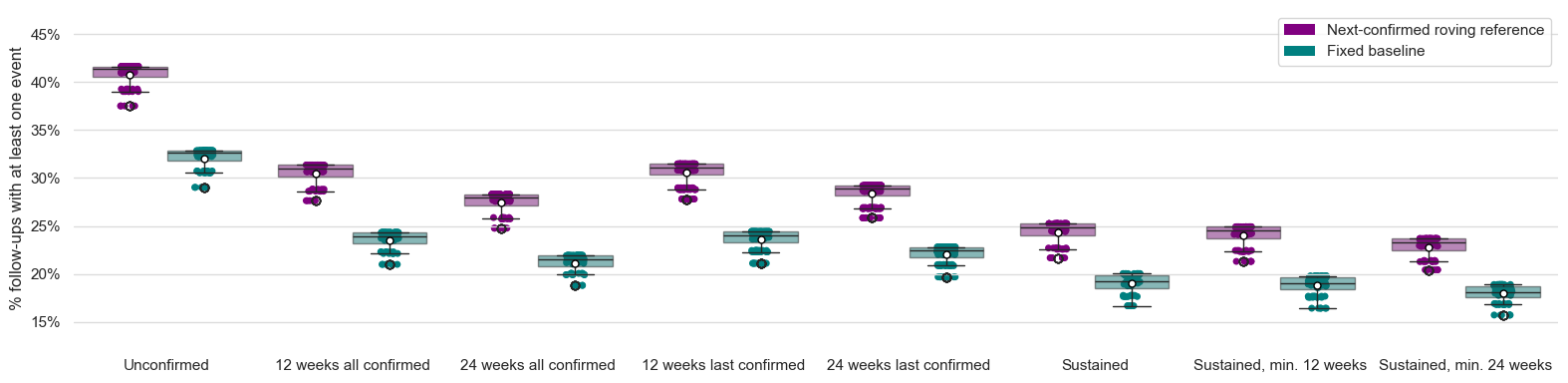


**eFigure 12**: Overall event rates for the 8 analyzed confirmation options, by baseline choice.

Mean **PIRA** rates ranged from 13.2% for sustained over a minimum of 24 weeks to 26.7% for unconfirmed disability accrual (eFigure 13A). Rates by baseline type are listed in eTable 13 and shown in eFigure 14A. Mean **PIRA confirmed in RAW** window rates ranged from 0% for unconfirmed disability accrual to 3.4% for sustained over a minimum of 24 weeks (eFigure 13B). Rates by baseline type are listed in eTable 14 and shown in eFigure 14B. Mean **RAW** rates ranged from 3.3% for sustained over a minimum of 12 weeks to 10.5% for unconfirmed disability accrual (eFigure 13C). Rates by baseline type are listed in eTable 15 and shown in eFigure 14C. Mean **undefined event** rates ranged from 2.6% for sustained over a minimum of 24 weeks to 3.8% for unconfirmed disability accrual (eFigure 13D). Rates by baseline type are listed in eTable 16 and shown in eFigure 14D.

|  | Mean PIRA rate (%) | | | | | | | |
| --- | --- | --- | --- | --- | --- | --- | --- | --- |
|  | Unconf. | 12 w. all | 24 w. all | 12 w. last | 24 w. last | Sustained | Sust. 12 w | Sust. 24 w |
| Fixed baseline | 22.7 | 17.8 | 15.8 | 17.9 | 16.4 | 12.6 | 12.4 | 11.6 |
| NC roving | 30.7 | 23.3 | 20.6 | 23.4 | 21.4 | 15.9 | 15.7 | 22.7 |

**eTable 13**: Mean PIRA rates for the 8 analyzed confirmation options, by baseline choice. **Abbreviations**: NC: Next confirmed, Sust: sustained, Unconf: unconfirmed, w: weeks.

|  | Mean PIRA with relapse during confirmation rate (%) | | | | | | | |
| --- | --- | --- | --- | --- | --- | --- | --- | --- |
|  | Unconf. | 12 w. all | 24 w. all | 12 w. last | 24 w. last | Sustained | Sust. 12 w | Sust. 24 w |
| Fixed baseline | 0.0 | 1.2 | 1.7 | 1.1 | 1.4 | 2.9 | 2.9 | 3.0 |
| NC roving | 0.0 | 1.7 | 2.4 | 1.5 | 2.0 | 3.8 | 3.8 | 3.9 |

**eTable 14**: Mean PIRA with relapse during confirmation rates for the 8 analyzed confirmation options, by baseline choice. **Abbreviations**: NC: Next-confirmed, Sust: sustained, Unconf: unconfirmed, w: weeks.

|  | Mean RAW rate (%) | | | | | | | |
| --- | --- | --- | --- | --- | --- | --- | --- | --- |
|  | Unconf. | 12 w. all | 24 w. all | 12 w. last | 24 w. last | Sustained | Sust. 12 w | Sust. 24 w |
| Fixed baseline | 9.1 | 4.2 | 3.6 | 4.4 | 4.2 | 2.9 | 2.8 | 3.0 |
| NC roving | 11.9 | 5.9 | 4.9 | 6.0 | 5.6 | 4.0 | 3.9 | 3.9 |

**eTable 15**: Mean RAW rates for the 8 analyzed confirmation options, by baseline choice. **Abbreviations**: NC: Next confirmed, Sust: sustained, Unconf: unconfirmed, w: weeks.

|  | Mean undefined event rate (%) | | | | | | | |
| --- | --- | --- | --- | --- | --- | --- | --- | --- |
|  | Unconf. | 12 w. all | 24 w. all | 12 w. last | 24 w. last | Sustained | Sust. 12 w | Sust. 24 w |
| Fixed baseline | 3.2 | 2.7 | 2.5 | 2.7 | 2.5 | 2.5 | 2.5 | 2.3 |
| NC roving | 4.3 | 3.4 | 3.2 | 3.4 | 3.2 | 3.0 | 3.0 | 2.8 |

**eTable 16**: Mean undefined event rates for the 8 analyzed confirmation options, by baseline choice. **Abbreviations**: NC: next-confirmed, Sust: sustained, Unconf: unconfirmed, w: weeks.


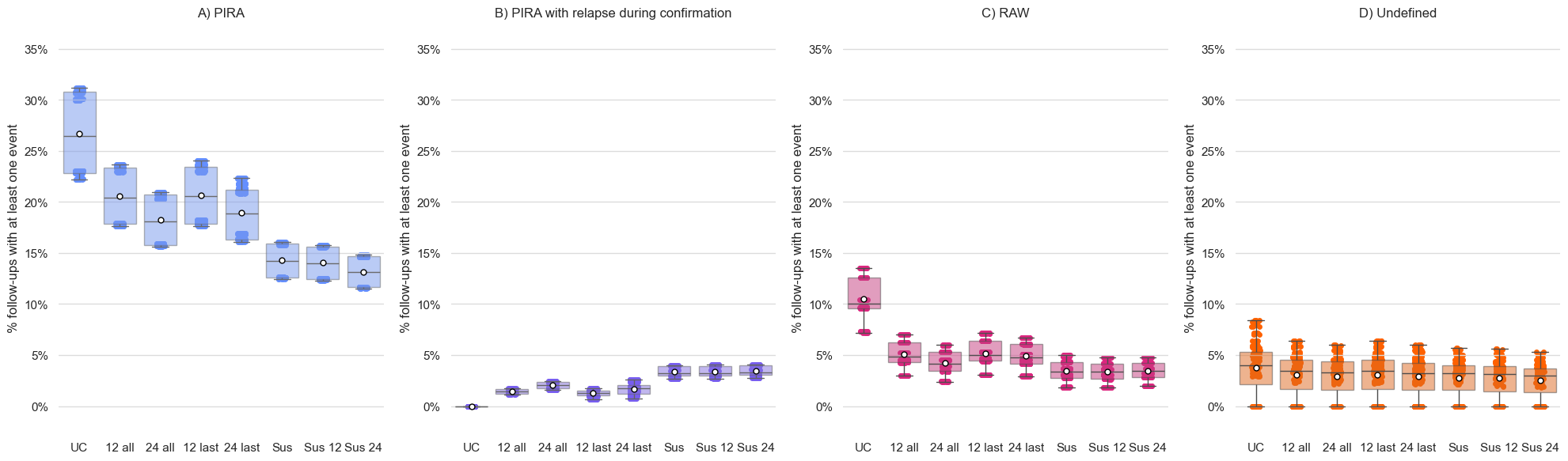


**eFigure 13**: Event rates by confirmation option. **A)** PIRA. **B)** PIRA confirmed in RAW window. **C)** RAW. **D)** Undefined. **Abbreviations**: UC: unconfirmed, 12 all: 12 weeks all-confirmed, 24 all: 24 weeks all-confirmed, 12 last: 12 weeks last confirmed, 24 last: 24 weeks last confirmed, Sus: sustained, Sus 12: sustained over at least 12 weeks, Sus 24: sustained over at least 24 weeks.


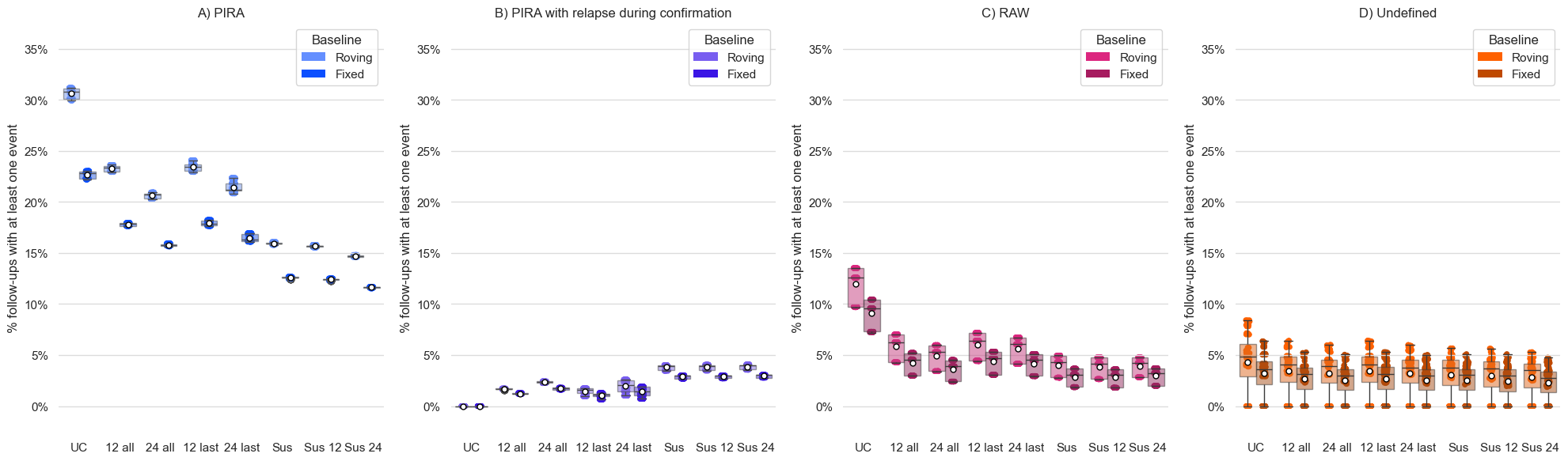


**eFigure 14**: Event rates by confirmation option, by baseline type choice. **A)** PIRA. **B)** PIRA with relapse during confirmation. **C)** RAW. **D)** Undefined. **Abbreviations**: UC: unconfirmed, 12 all: 12 weeks all-confirmed, 24 all: 24 weeks all-confirmed, 12 last: 12 weeks last confirmed, 24 last: 24 weeks last confirmed, Sus: sustained, Sus 12: sustained over at least 12 weeks, Sus 24: sustained over at least 24 weeks.

### Event type distribution by confirmation condition

The mean contribution of **PIRA** to the total event count ranged from 61.1% for sustained over a minimum of 24 weeks to 73.21% for 12 weeks last confirmed. The mean contribution of **PIRA with relapse during confirmation** to the total event count ranged from 0.0% for unconfirmed disability accrual to 15.7% for sustained over a minimum of 24 weeks. The mean contribution of **RAW** to the total event count ranged from 12.4% for sustained over a minimum of 12 weeks to 22.6% for unconfirmed, and the mean contribution of **undefined worsening** to the total event count ranges from 7.4% for unconfirmed disability accrual to 9.9% for sustained (no minimal confirmation distance) (eTable 17, eFigure 15).

|  | Mean event type contribution (%) | | | | | | | |
| --- | --- | --- | --- | --- | --- | --- | --- | --- |
|  | Unconf. | 12 w. all | 24 w. all | 12 w. last | 24 w. last | Sustained | Sust. 12 w | Sust. 24 w |
| PIRA | 70.0 | 73.0 | 70.7 | 73.2 | 70.9 | 63.1 | 62.9 | 61.1 |
| PIRA rel. conf. | 0.0 | 4.1 | 6.5 | 3.5 | 5.1 | 14.5 | 14.9 | 15.7 |
| RAW | 22.6 | 14.5 | 13.8 | 14.9 | 15.4 | 12.5 | 12.4 | 13.5 |
| Undefined | 7.4 | 8.5 | 9.0 | 8.4 | 8.6 | 9.9 | 9.8 | 9.7 |

**eTable 17**: Mean event type contributions for the 8 analyzed confirmation options. **Abbreviations**: NC: Next confirmed, Sust: sustained, Unconf: unconfirmed, w: weeks.


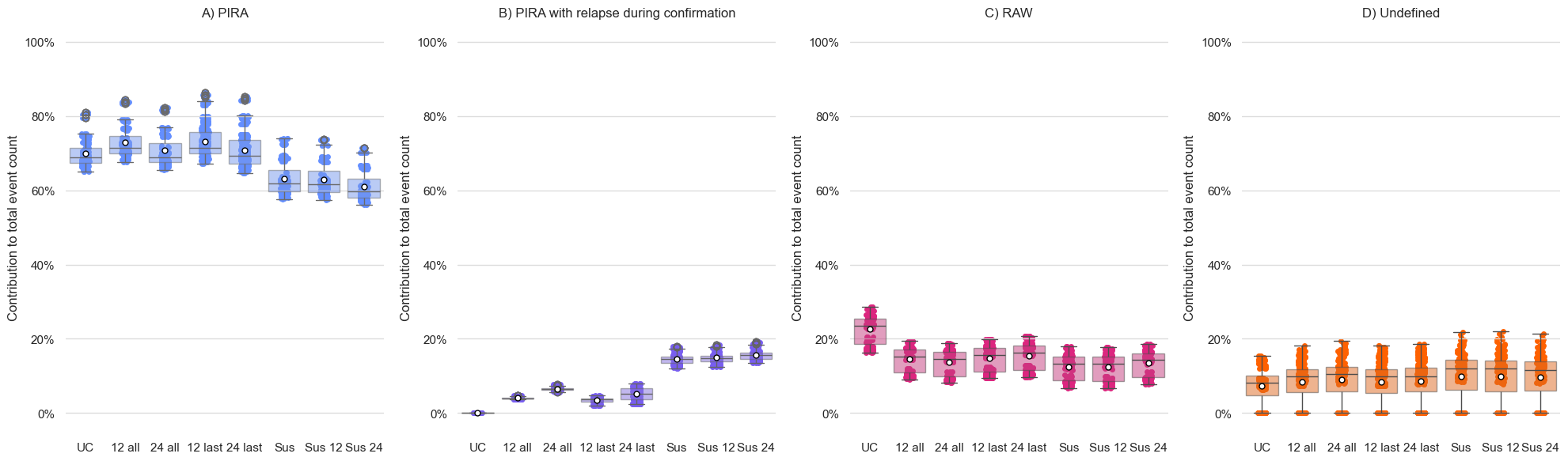


**eFigure 15**: Event type distributions by confirmation option. **A)** PIRA. **B)** PIRA with relapse during confirmation. **C)** RAW. **D)** Undefined. **Abbreviations**: UC: unconfirmed, 12 all: 12 weeks all-confirmed, 24 all: 24 weeks all-confirmed, 12 last: 12 weeks last confirmed, 24 last: 24 weeks last confirmed, Sus: sustained, Sus 12: sustained over at least 12 weeks, Sus 24: sustained over at least 24 weeks.

# Sensitivity analyses

All but two follow-ups were treated at baseline with one of 11 DMT, where 1228 (34.8%) were treated with Natalizumab, 1065 (30.2%) with Fingolimod, and 1230 (34.9%) with other DMT. Median follow-up durations ranged from 2.1 years (Ozanimod) to 6.6 years (Ofatumumab, 1 follow-up only), and median baseline EDSS ranged from 1.0 (Daclizumab, Ozanimod) to 6.5 (Siponimod) (eTable 18).

| DMT at baseline | Number of follow-ups | Median duration* (years) | Median baseline age (years) | Median baseline EDSS** | % female patients | Start of first follow-up | Start of last follow-up |
| --- | --- | --- | --- | --- | --- | --- | --- |
| Alemtuzumab | 41 | 4.8 | 33.0 | 2.5 | 68.3 | 2014-01-23 | 2020-01-13 |
| Cladribine | 52 | 2.7 | 35.5 | 1.5 | 82.7 | 2018-06-13 | 2021-02-26 |
| Daclizumab | 1 | 5.2 | 35.0 | 1.0 | 100.0 | 2017-07-14 | 2017-07-14 |
| Dimethyl fumarate | 775 | 3.6 | 36.0 | 1.5 | 66.8 | 2012-10-08 | 2021-05-31 |
| Fingolimod | 1065 | 4.6 | 39.0 | 2.0 | 67.3 | 2010-10-28 | 2021-05-10 |
| Natalizumab | 1228 | 5.4 | 34.0 | 2.5 | 70.8 | 2006-08-10 | 2021-05-18 |
| No DMT | 2 | 4.1 | 43.0 | 3.25 | 50.0 | 2007-12-17 | 2008-02-26 |
| Ocrelizumab | 68 | 2.9 | 36.5 | 2.5 | 54.4 | 2018-02-27 | 2021-05-20 |
| Ofatumumab | 1 | 6.6 | 30.0 | 2.5 | 100.0 | 2016-06-22 | 2016-06-22 |
| Ozanimod | 3 | 2.1 | 32.0 | 1.0 | 66.7 | 2021-03-11 | 2021-04-23 |
| Siponimod | 3 | 2.2 | 48.0 | 6.5 | 33.3 | 2020-08-25 | 2021-06-10 |
| Teriflunomide | 286 | 3.7 | 43.0 | 1.5 | 60.5 | 2013-04-29 | 2021-06-02 |

**eTable 18**: Follow-ups by DMT at baseline. * Duration of the follow-up, not the baseline DMT. ** Interpolated.

The number of follow-ups, the median duration of follow-ups, and the number of events irrespective of type per group are shown in eTable 19.

|  |  |  | Total events irrespective of type | | |
| --- | --- | --- | --- | --- | --- |
| DMT group | Follow-ups | Median duration (years) | Min | Median | Max |
| All follow-ups | **3525** | **4.3** | **697** | **1133** | **2352** |
| HE-DMT | 1339 | 5.1 | 309 | 510 | 1084 |
| ME-DMT | 1123 | 4.5 | 240 | 387 | 769 |
| LE-DMT | 1061 | 3.6 | 147 | 239 | 496 |
| No HE-DMT | 2186 | 4.0 | 388 | 628 | 1268 |
| No ME-DMT | 2402 | 4.3 | 457 | 748 | 1583 |
| No LE-DMT | 2464 | 4.8 | 550 | 896 | 1856 |
| No Dimethyl Fumarate | 2750 | 4.7 | 607 | 987 | 2026 |
| No Fingolimod | 2460 | 4.2 | 463 | 755 | 1603 |
| No Natalizumab | 2297 | 3.9 | 407 | 661 | 1333 |
| No Teriflunomide | 3239 | 4.4 | 640 | 1048 | 2182 |

**eTable 19**: Median duration of follow-up in years and total event counts (irrespective of type) for HE-, ME-, and LE-DMT only, and for leave-one-DMT/DMT group-out subgroups.

Under a leave-one-DMT-out approach, the deltas between minimal and maximal PIRA and RAW contributions ranged from 27.9 to 31.9 (30.2 for all follow-ups) and from 20.2 to 22.6 (21.9 for all follow-ups), respectively (eTable 20, eFigure 16).

|  |  | PIRA contribution (%) | | | RAW contribution (%) | | |
| --- | --- | --- | --- | --- | --- | --- | --- |
| DMT group | Follow-ups | Min | Mean | Max | Min | Mean | Max |
| All follow-ups | **3525** | **56.1** | **68.9** | **86.3** | **6.7** | **15.0** | **28.6** |
| HE-DMT | 1339 | 55.7 | 71.5 | 89.6 | 4.9 | 12.9 | 25.7 |
| ME-DMT | 1123 | 56.9 | 68.6 | 86.0 | 7.4 | 15.4 | 29.0 |
| LE-DMT | 1061 | 52.1 | 63.7 | 81.4 | 8.6 | 18.8 | 33.9 |
| No HE-DMT | 2186 | 55.7 | 66.8 | 83.8 | 8.0 | 16.7 | 30.7 |
| No ME-DMT | 2402 | 54.6 | 69.0 | 86.8 | 6.1 | 14.8 | 28.6 |
| No LE-DMT | 2464 | 57.0 | 70.3 | 87.7 | 6.1 | 14.0 | 26.9 |
| No Dimethyl Fumarate | 2750 | 57.0 | 70.0 | 87.5 | 6.4 | 14.2 | 26.6 |
| No Fingolimod | 2460 | 54.7 | 68.9 | 86.6 | 6.4 | 14.9 | 29.0 |
| No Natalizumab | 2297 | 55.4 | 66.7 | 83.3 | 8.2 | 16.9 | 30.6 |
| No Teriflunomide | 3239 | 56.0 | 69.0 | 86.3 | 6.5 | 14.9 | 28.9 |

**eTable 20**: PIRA and RAW contributions to the total event counts for HE-, ME-, and LE-DMT only, and for leave-one-DMT/DMT group-out subgroups.


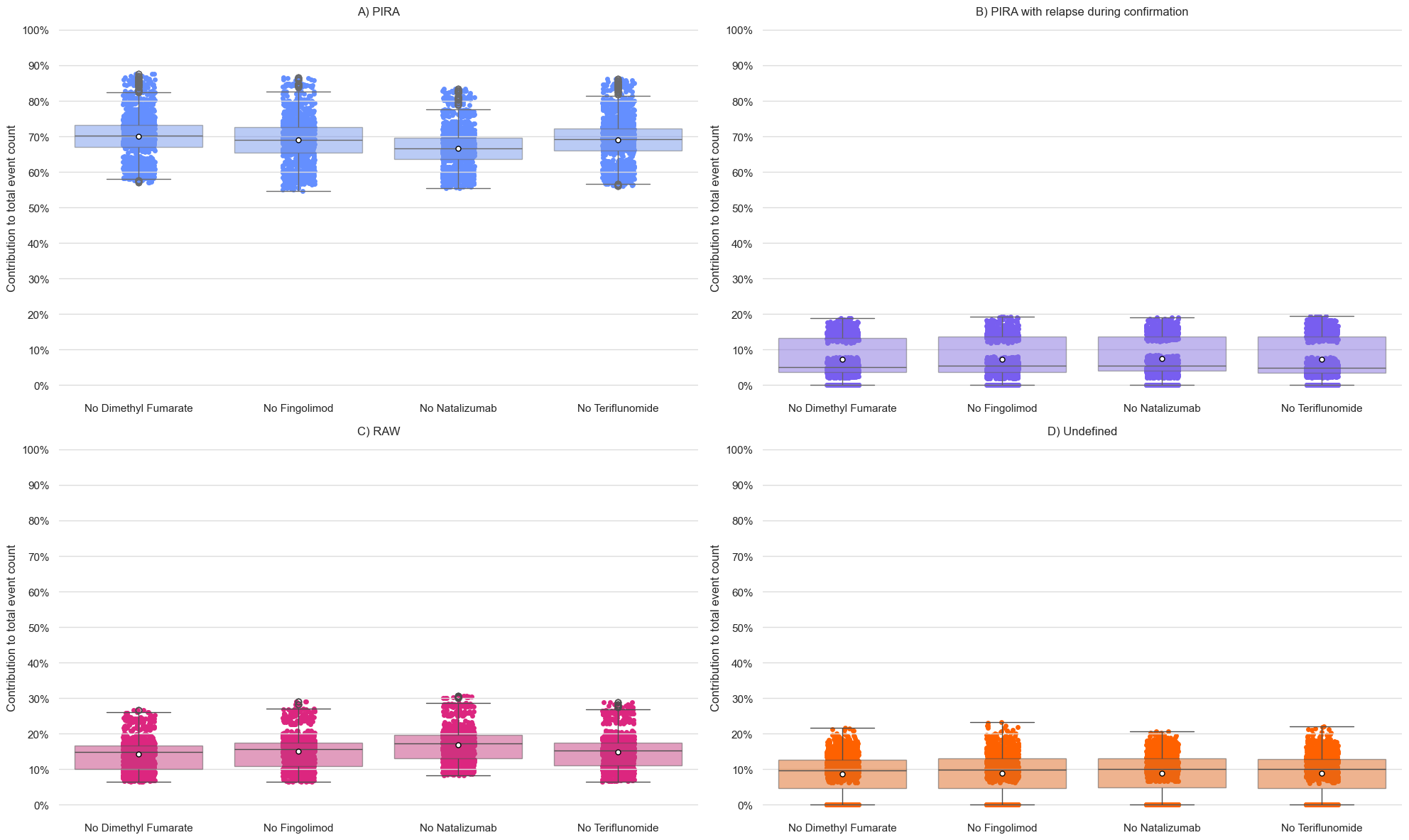


**eFigure 16**: Event type contributions under leave-one-DMT-out. **A)** PIRA contribution. **B)** PIRA with relapse during confirmation contribution. **C)** RAW contribution. **D)** Undefined worsening contribution.


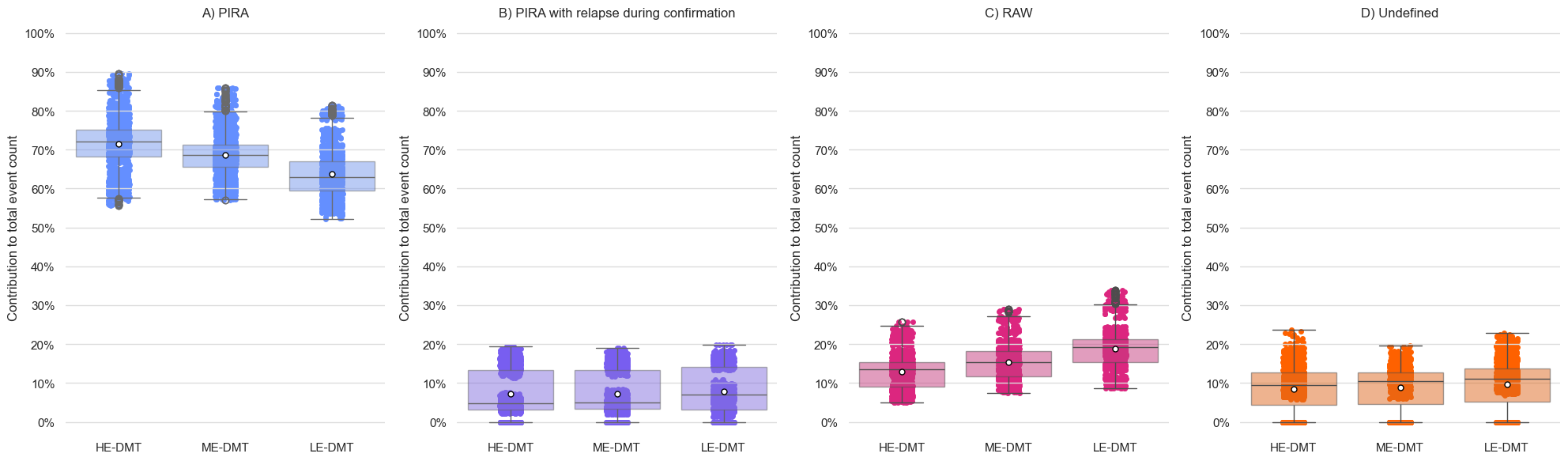


**eFigure 17**: Event type contributions by DMT efficacy class. **A)** PIRA contribution. **B)** PIRA with relapse during confirmation contribution. **C)** RAW contribution. **D)** Undefined worsening contribution.


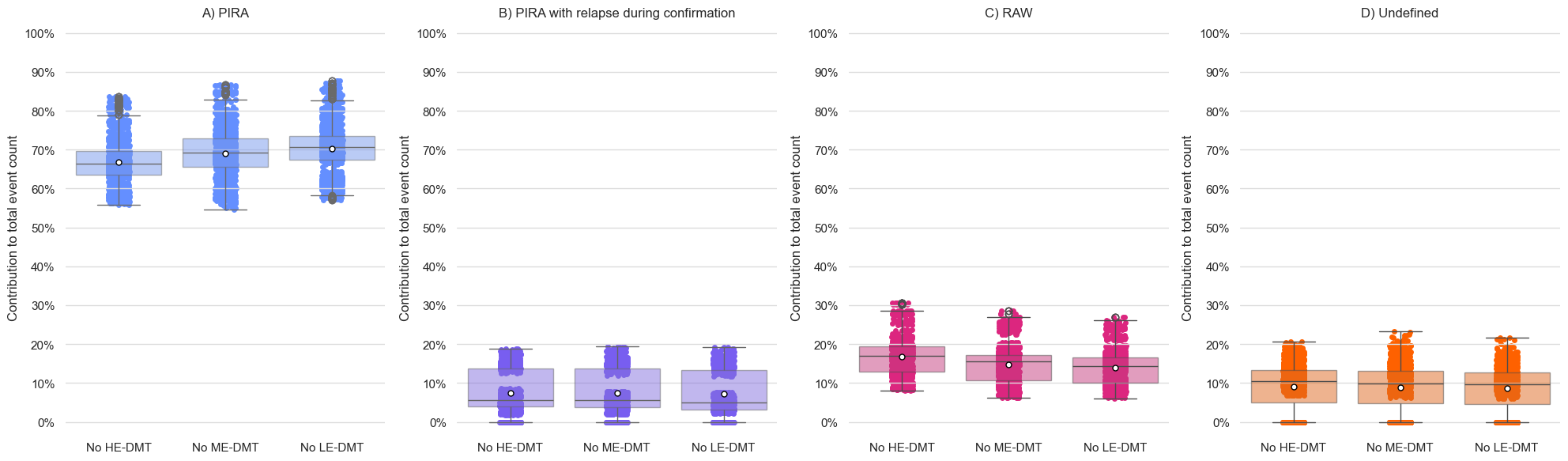


**eFigure 18**: Event type contributions under leave-one-DMT group-out. **A)** PIRA contribution. **B)** PIRA with relapse during confirmation contribution. **C)** RAW contribution. **D)** Undefined worsening contribution.

# Supplemental subgroup analyses

## Follow-ups with at least one relapse only

### Cohort

For the subgroup analysis of follow-ups with at least one relapse during the follow-up period, we analyzed 1,361 follow-ups of 1,317 patients with RMS (898 (68.2%) female, 419 (31.8%) male). 1,274 (96.7%) patients had one follow-up, 42 (3.2%) had two follow-ups, 1 (0.1%) had three follow-ups.

Of the 1,361 follow-ups, 929 (68.3%) were from female patients. The mean age at baseline was 36.6 years (IQR 29.0-44.0 years, min. 18.0 years, max. 67.0 years), the median EDSS at baseline was 2.0 (IQR 1.5-3.5, min. 0.0, max. 7.5), and the median duration of follow-up was 5.1 years (IQR 3.3 -7.4, min. 2.0, max. 16.2).

### Event rate and event type contribution

**Overall event rates** (fraction of follow-ups with at least one disability accrual event) ranged from 17.3% to 59.7% with a mean event rate of 34.6% (eFigure 19). The **overall median time to event** (time to first event irrespective of type) ranged from 4.0 years to 15.7 years (mean 9.6 years).


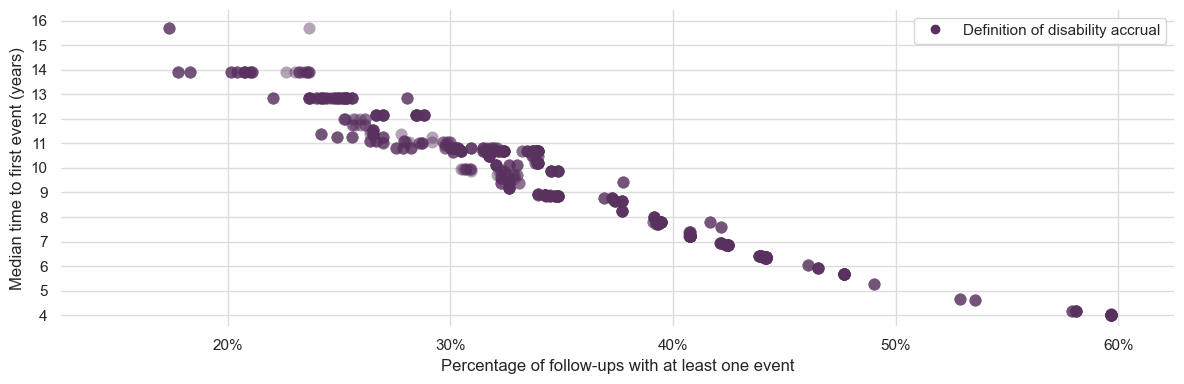


**eFigure 19**: Overall event rates (fraction of follow-ups with at least one event irrespective of type) vs. median time to first event for 1,440 definitions of RAW and PIRA. Each dot represents a definition.

**PIRA** event rates (fraction of follow-ups with at least one PIRA event) ranged from 6.5% to 32.6% with a mean event rate of 16.6%, **PIRA with relapse during confirmation** event rates (fraction of follow-ups with at least one PIRA confirmed in RAW window event) ranged from 0% to 10.6% (mean event rate 5.0%), and **RAW** event rates (fraction of follow-ups with at least one RAW event) ranged from 4.6% to 35.0% with a mean event rate of 13.0%. **Undefined events** were present in 0% to 21.8% of the follow-ups (mean 7.6%) (eFigure 20, eTable 21).

|  | Event rate (%) | | | Median time to first event (years) | | |
| --- | --- | --- | --- | --- | --- | --- |
|  | Min | Mean | Max | Min | Mean | Max |
| Disability accrual (overall) | **17.3** | **34.6** | **59.7** | **4.0** | **9.6** | **15.7** |
| PIRA | 6.5 | 16.6 | 32.6 | 9.0 | 13.6 | 15.7 |
| PIRA with relapse during conf. | 0.0 | 5.0 | 10.6 | - | - | - |
| RAW | 4.6 | 13.0 | 35.0 | - | - | - |
| Undefined | 0.0 | 7.6 | 21.8 | - | - | - |

**eTable 21**: Event rate (fraction of follow-ups with at least one event) and median time to first event (Kaplan-Meier) ranges, overall and by event type for 1,440 definitions of RAW/PIRA.


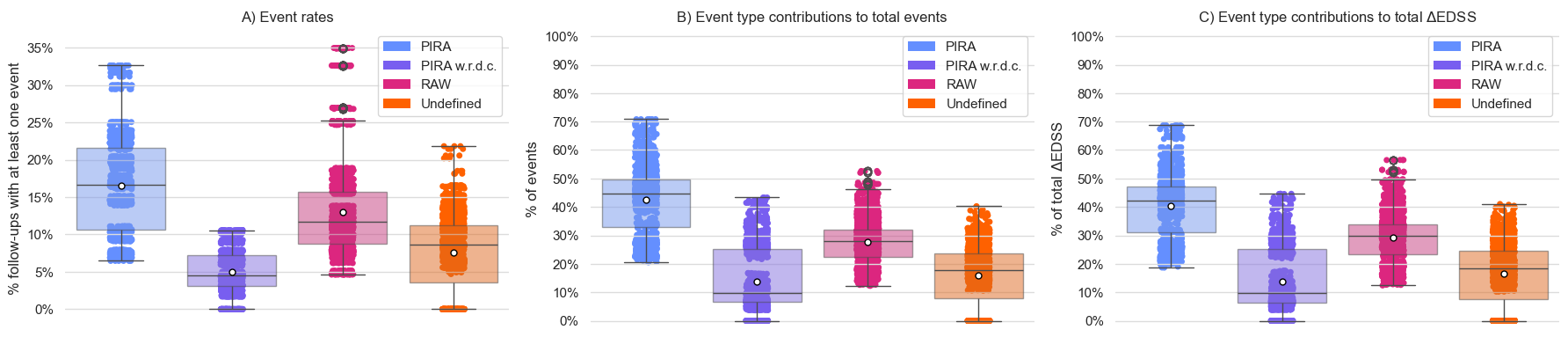


**eFigure 20**: Event rates and contributions by event type for 1,440 definitions of RAW and PIRA. **A)** Event rates (fraction of follow-ups with at least one event of a given type). **B)** Event type contribution (number of events of a given type over total number of events). **C)** Event type contribution to the total ΔEDSS. **Abbreviations**: PIRA w.r.d.c.: PIRA with relapse during confirmation.

**Overall**, between 303 and 1,404 (median 618) events of any type and total EDSS score increase from 362.5 to 1,818.5 (median 757.0) points were observed. Of all disability accrual events, between 20.5% and 70.8% (mean 41.6%) were **PIRA**, 12.3% to 52.6% (mean 27.7%) were **RAW**, and 0% to 40.2% (mean 15.9%) were **undefined**. Of the total EDSS score increase, 18.7% to 68.7% (mean 40.3%) was due to **PIRA**, 12.4% to 56.5% (mean 29.3%) was due to **RAW**, and 0% to 41.0% (mean 16.5%) was due to **undefined** worsening.

|  | Contribution to total events (%) | | | Contribution to total ΔEDSS (%) | | |
| --- | --- | --- | --- | --- | --- | --- |
|  | Min | Mean | Max | Min | Mean | Max |
| PIRA | 20.5 | 42.6 | 70.8 | 18.7 | 40.3 | 68.7 |
| PIRA with relapse during conf. | 0.0 | 13.8 | 43.3 | 0.0 | 13.9 | 44.6 |
| RAW | 12.3 | 27.7 | 52.6 | 12.4 | 29.3 | 56.5 |
| Undefined | 0.0 | 15.9 | 40.2 | 0.0 | 16.5 | 41.0 |

**eTable 22**: Contribution of each event type to the total number of events and to the total EDSS score increase.

**PIRA** was the only event type in 3.6% to 23.8% (mean 11.4%) of all follow-ups, and **RAW** was the only event type in 3.0% to 23.6% (mean 8.8%) of all follow-ups. Of the follow-ups with events, 14.1% to 63.2% (mean 32.7%) had **PIRA** only, 11.4% to 51.6% (mean 25.1%) had **RAW** only, and 0% to 34.3% (mean 13.5%) only had undefined worsening.

|  | Fraction of follow-ups (%) | | | Of follow-ups with events (%) | | |
| --- | --- | --- | --- | --- | --- | --- |
|  | Min | Mean | Max | Min | Mean | Max |
| PIRA only | 3.6 | 11.4 | 23.8 | 14.1 | 32.7 | 63.2 |
| PIRA w. relapse during conf. only | 0.0 | 2.8 | 8.2 | 0.0 | 9.5 | 35.2 |
| RAW only | 3.0 | 8.8 | 23.6 | 11.4 | 25.1 | 51.6 |
| Undefined only | 0.0 | 4.8 | 11.1 | 0.0 | 13.5 | 34.3 |

**eTable 23**: Fraction of follow-ups where a given event type is the single contributor to disability accrual (left) and fraction of follow-ups with events where a given event type is the single contributor to disability accrual (right). For example, of all 1,361 follow-ups, up to 23.8% (depending on the definition) have PIRA but no disability accrual of any other type. Of all follow-ups with disability accrual (up to 59.7% of all follow-ups, depending on the definition), up to 63.2% only experience PIRA.

### Impact of individual definition aspects


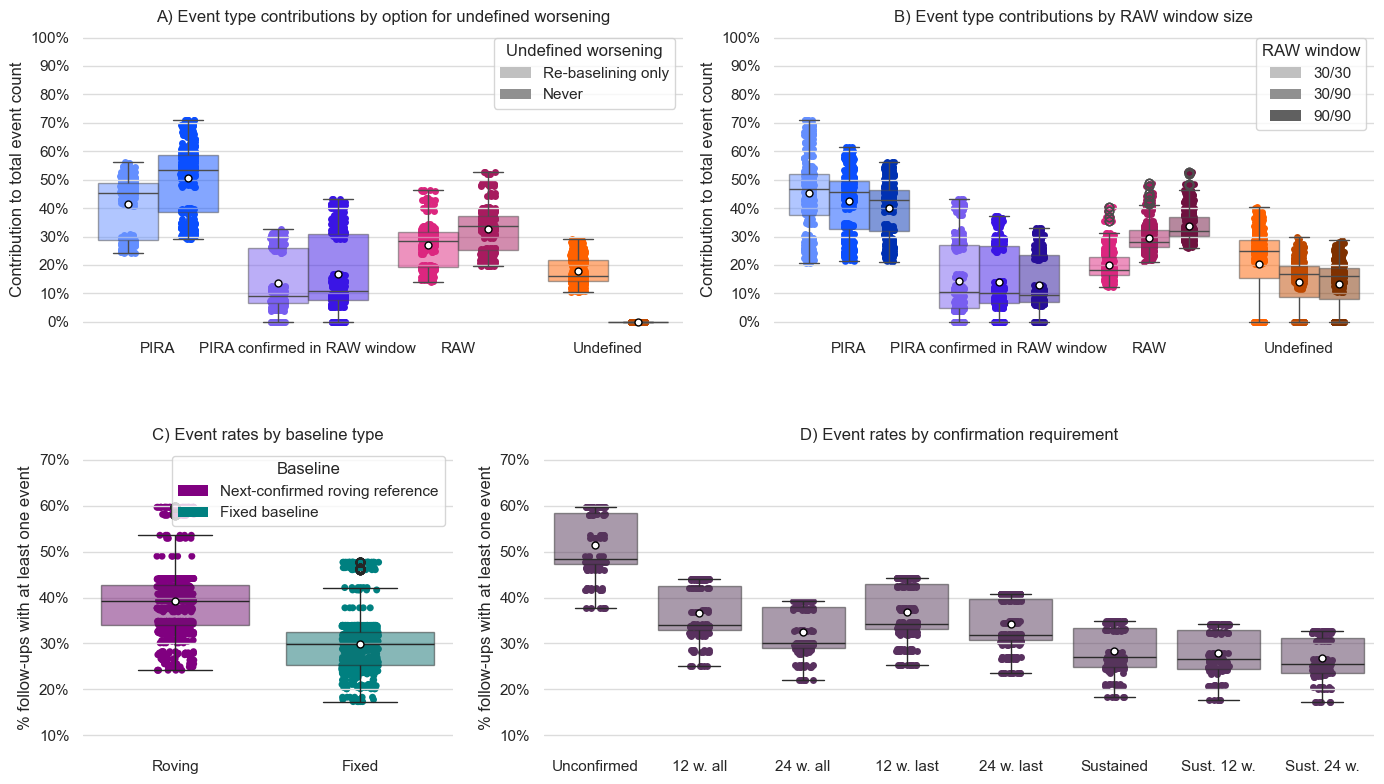


**eFigure 21**: Impact of individual aspects of disability accrual definition. **A)** Event type contributions by undefined worsening option, “re-baselining only” and “never” options only. **B)** Event type contributions by RAW window size. **C)** Overall event rates (irrespective of type) by baseline option. **D)** Overall event rates (irrespective of type) by confirmation requirement. **Abbreviations**: Sust.: sustained, w.: weeks.

Omitting events identified at post-relapse re-baselining assessments significantly increases the relative contributions of PIRA and RAW to the total event count, compared to the default re-baselining only mode (from an average 41.5% to 50.7% for PIRA, and from 27.0% to 32.6% for RAW, p < 0.001 for both comparisons) (eFigure 21A).

Expanding the RAW window from 30 days pre- and post-relapse (30/30) to 30/90 or 90/90 reduced the average PIRA contribution from 45.2% to 42.4% and 40.1%, respectively (p < 0.001), while increasing the average RAW contribution from 19.9% to 29.5% and 33.7%, respectively (p < 0.001) (eFigure 21B).

Using a roving reference confirmed at the next assessment instead of a fixed baseline increased the average overall event rate from 30.0% to 39.1% (p < 0.001) (eFigure 21C).

Under different confirmation requirements the mean overall event rate ranged from 26.7% for sustained over ≥ 24 weeks to 51.4% for unconfirmed disability accrual (eFigure 21D).

## Follow-ups with more conservative inclusion criteria

### Cohort

For the subgroup analysis of follow-ups with at least 4 EDSS scores over at least 2 years with at least one EDSS score every six months, we analyzed 2,098 follow-ups of 1,837 patients with RMS (1,245 (67.8%) female, 592 (32.2%) male). 1,590 (86.6%) patients had one follow-up, 233 (12.7%) had two follow-ups, 14 (0.8%) had three follow-ups.

Of the 2,098 follow-ups, 1,429 (68.1%) of follow-ups are from female patients, 669 (31.9%) from male patients. The mean age at baseline was 37.9 years (min. 18.0 years, max. 73.0 years, IQR 30.0-45.0 years), the median EDSS at baseline was 2.0 (IQR 1.0- 3.5, min. 0.0, max. 8.5), and the median duration of follow-up was 3.3 years (IQR 2.5-4.9, min. 2.0, max. 13.5). 1,002 (47.8%) follow-ups have no documented relapse; 715 (34.1%) follow-ups have at least one documented relapse during the follow-up period, and 381 (18.1%) follow-ups have at least one documented relapse outside the analyzed period.

Of the 715 follow-ups with at least one relapse during the follow-up period, 384 (53.7%) had one relapse, 185 (25.9%) had two relapses, 75 (10.5%) had three relapses, 32 (4.5%) had four relapses, and 39 (5.5%) had more than four relapses (the max. observed number of relapses was 17).

### Event rate and event type contribution

**Overall event rates** (fraction of follow-ups with at least one event) ranged from 13.3% to 37.1% with a mean event rate of 22.0%. The **overall median time to event** (time to first event irrespective of type, Kaplan-Meier, for 888 definitions with a sufficiently high event rate) ranged from 5.7 years to 12.0 years (mean 10.8 years) (eFigure 22, eTable 24).


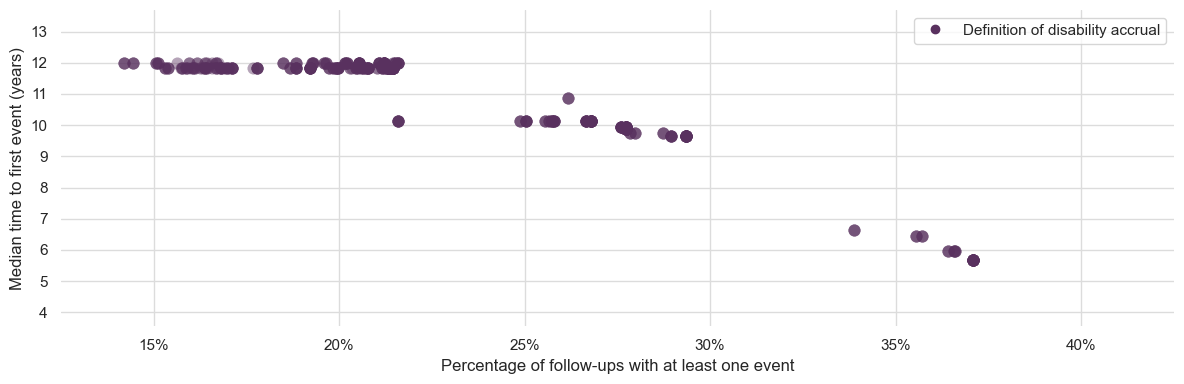


**eFigure 22**: Overall event rates (fraction of follow-ups with at least one event irrespective of type) vs. median time to first event for 888 definitions of RAW and PIRA. Each dot represents a definition.

**PIRA** event rates ranged from 9.2% to 28.4% with a mean event rate of 16.1%, **PIRA with relapse during confirmation** event rates (fraction of follow-ups with at least one PIRA with relapse during confirmation event) ranged from 0% to 3.8% (mean event rate 1.8%), and **RAW** event rates (fraction of follow-ups with at least one RAW event) ranged from 1.5% to 12.6% with a mean event rate of 4.4%. **Undefined events** were present in 0% to 6.9% of the follow-ups (mean 2.1%) (eTable 24, eFigure 23A).


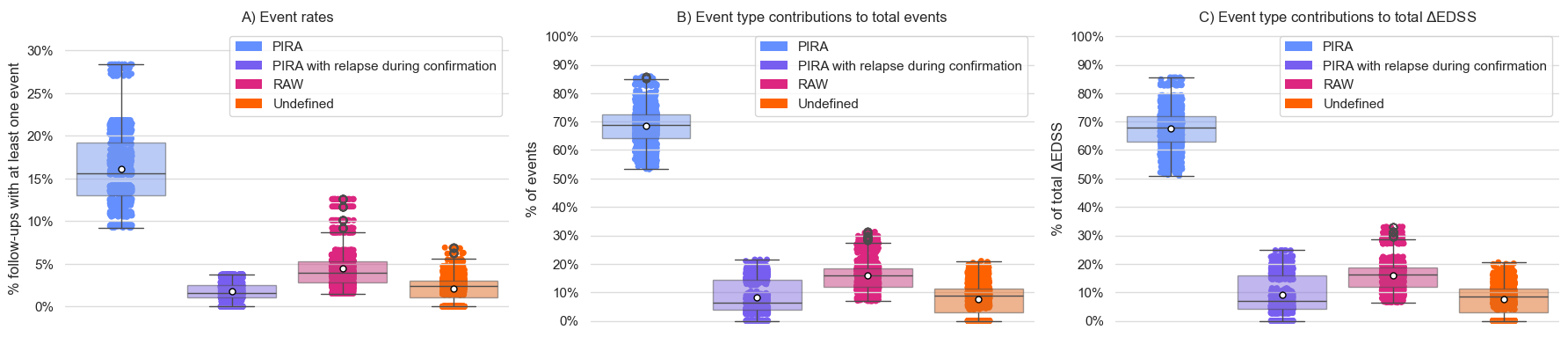


**eFigure 23**: Event rates and contributions by event type for 1,440 definitions of RAW and PIRA. **A)** Event rates (fraction of follow-ups with at least one event of a given type). **B)** Event type contribution (number of events of a given type over total number of events). **C)** Event type contribution to the total ΔEDSS.

Median time to **PIRA** event ranged from 7.9 years to 12.0 years (mean 11.0 years, 306 definitions with a sufficiently high event rate). Median time to **PIRA with relapse during confirmation**, **RAW** or **undefined** event could not be determined due to too low event rates.

|  | Event rate (%) | | | Median time to first event (years)* | | |
| --- | --- | --- | --- | --- | --- | --- |
|  | Min | Mean | Max | Min | Mean | Max |
| Disability accrual (overall) | **13.3** | **22.0** | **37.1** | **5.7** | **10.8** | **12.0** |
| PIRA | 9.2 | 16.1 | 28.4 | 7.9 | 11.0 | 12.0 |
| PIRA with relapse during conf. | 0.0 | 1.8 | 3.8 | - | - | - |
| RAW | 1.5 | 4.4 | 12.6 | - | - | - |
| Undefined | 0.0 | 2.1 | 6.9 | - | - | - |

**eTable 24**: Event rate ranges, overall and by event type for 1,440 definitions of RAW/PIRA, and median time to first event (Kaplan-Meier) ranges for 888 definitions (overall) and 306 definitions (PIRA). * Not all definitions yield a sufficient number of events to determine the median time to first event. Overall: 888 definitions; PIRA: 306 definitions.

**Overall**, between 331 and 1,223 (median 558) events of any type and total EDSS score increases from 386.5 to 1,533.0 (median 663.5) points were observed. Of all disability accrual events, between 53.2% and 85.9% (mean 68.5%) were **PIRA**, and 6.9% to 31.2% (mean 15.8%) were **RAW**. Of the total EDSS score increase, 50.9% to 85.5% (mean 67.5%) was due to **PIRA**, and 6.4% to 33.0% (mean 16.0%) was due to **RAW**.

|  | Contribution to total events (%) | | | Contribution to total ΔEDSS (%) | | |
| --- | --- | --- | --- | --- | --- | --- |
|  | Min | Mean | Max | Min | Mean | Max |
| PIRA | 53.2 | 68.5 | 85.9 | 50.9 | 67.5 | 85.5 |
| PIRA with relapse during conf. | 0.0 | 8.1 | 21.5 | 0.0 | 9.0 | 24.7 |
| RAW | 6.9 | 15.8 | 31.2 | 6.4 | 16.0 | 33.0 |
| Undefined | 0.0 | 7.6 | 21.0 | 0.0 | 7.5 | 20.5 |

**eTable 25**: Contribution of each event type to the total number of events and to the total EDSS score increase.

**PIRA** was the only event type in 8.5% to 25.2% (mean 14.5%) of all follow-ups, and of the follow-ups with events, 53.8% to 83.3% (mean 65.8%) had **PIRA** only.

|  | Fraction of follow-ups (%) | | | Fraction of follow-ups with events (%) | | |
| --- | --- | --- | --- | --- | --- | --- |
|  | Min | Mean | Max | Min | Mean | Max |
| PIRA only | 8.5 | 14.5 | 25.2 | 53.8 | 65.8 | 83.3 |
| PIRA w. relapse during conf. only | 0.0 | 1.0 | 2.8 | 0.0 | 5.0 | 16.5 |
| RAW only | 0.9 | 2.9 | 8.4 | 5.1 | 13.0 | 29.6 |
| Undefined only | 0.0 | 1.3 | 3.5 | 0.0 | 6.1 | 17.4 |

**eTable 26**: Fraction of follow-ups where a given event type is the single contributor to disability accrual (left) and fraction of follow-ups with events where a given event type is the single contributor to disability accrual (right).

### Impact of individual definition aspects

Omitting events identified at post-relapse re-baselining assessments significantly increases the relative contributions of PIRA and RAW to the total event count, compared to the default re-baselining only mode (from an average 68.4% to 74.2% for PIRA, and from 15.8% to 17.0% for RAW, p < 0.001 for both comparisons) (eFigure 24A).

Expanding the RAW window from 30 days pre- and post-relapse (30/30) to 30/90 or 90/90 reduced the average PIRA contribution from 70.4% to 68.4% and 66.6%, respectively (p < 0.001), while increasing the average RAW contribution from 11.2% to 16.7% and 19.5%, respectively (p < 0.001) (eFigure 24B).

Using a roving reference confirmed at the next assessment instead of a fixed baseline increased the average overall event rate from 19.2% to 24.7% (p < 0.001) (eFigure 24C).

Under different confirmation requirements the mean overall event rate ranged from 17.0% for sustained over ≥ 24 weeks to 32.6% for unconfirmed disability accrual (eFigure 24D).


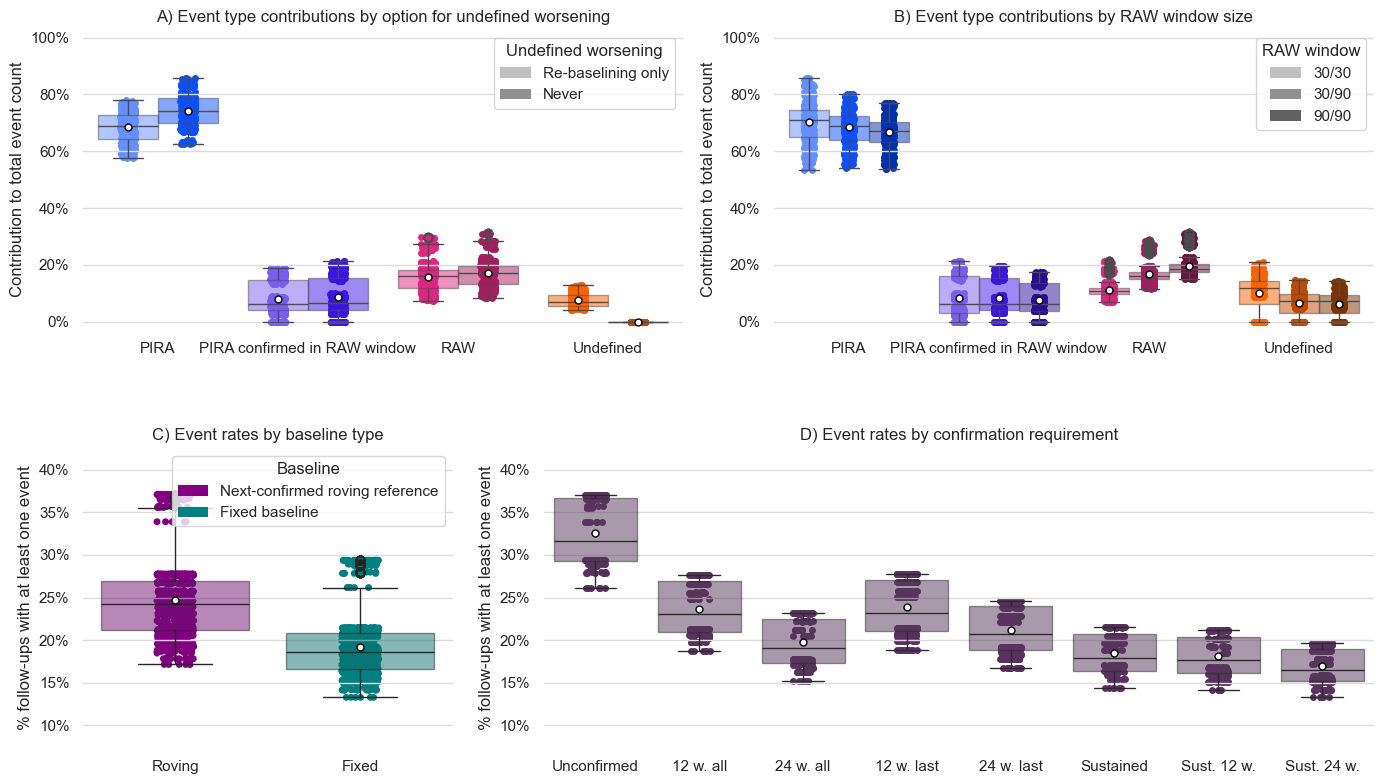


**eFigure 24**: Impact of individual aspects of disability accrual definition. **A)** Event type contributions by undefined worsening option, “re-baselining only” and “never” options only. **B)** Event type contributions by RAW window size. **C)** Overall event rates (irrespective of type) by baseline option. **D)** Overall event rates (irrespective of type) by confirmation requirement. **Abbreviations**: Sust.: sustained, w.: weeks.

# Analysis of the harmonized PIRA definition

## Methods

Our algorithm identifies PIRA events alongside RAW events and the additional categories “PIRA with relapse during confirmation” and “undefined worsening” for ambiguous events. Therefore, there are parameters covering disability accrual event detection and classification that are not explicitly mentioned (or not required) for the proposed harmonized PIRA definition (see main manuscript) ^1^. eTable 27 describes the 360 parameter choices that reproduce the harmonized PIRA definition as closely as possible.

| Option | Parameter choice for reproducing Müller et al. | N tested |
| --- | --- | --- |
| Event merging | Not mentioned/discussed. We assume that there is no merging, thus chose **False**. | 1 |
| Undefined events | Not mentioned/discussed. We propose using **re-baselining only**, but tested all four options. | 4 |
| Constraints for undefined | Not mentioned/discussed. We propose using **unconstrained**, but tested all three options. | 3 |
| Baseline | **Roving reference**. The text recommends **confirmation** at the next assessment, Supplementary Figure 3 requires confirmation at > 3 months without specifying whether the confirmation requirement applies to all values within the confirmation interval (“all”) or only the first value that satisfies the confirmation distance requirement. We tested **next-confirmed**, **3 months** (≥ 3 * 30 = 90 days) **all** confirmed, and **3 months last** confirmed. The new reference is the maximum of all confirmation scores. | 3 |
| Minimal increase | + 1.5 for reference 0, + 1.0 for reference < 5.5, + 0.5 else | 1 |
| Confirmation | The text recommends confirmation **over at least 3 months**, preferably 6 or 12, Supplementary Figure 3 requires > 3 months, without specifying whether “all” or “last”, although parts b), c) of Supplementary Figure 3 might imply “all”. Sustained must be ≥ 12 months after the event. For sustained, according to Supplementary Figure 3 c), d), all values are included. We tested **3 months** (90 days), **6 months** (180 days), and **12 months** (360 days) **“all” and “last”** confirmed, and **sustained for at least 12 months** (360 days) with “all” by default. | 7 |
| Confirmation type | **Minimum** as specified in the text and in Supplementary Figure 3. | 1 |
| Require confirmation for last visit | Not mentioned/discussed, we tested **True**. | 1 |
| Left hand side confirmation tolerance | Not mentioned/discussed, we tested **0** (i.e. earliest confirmation assessment for confirmation at x days is at day x). | 1 |
| Right hand side confirmation tolerance | Not mentioned/discussed, we tested **∞** (i.e. confirmation assessment can be at any time after the minimal confirmation distance). | 1 |
| RAW window size | **30 days pre**-relapse, **90 days post**-relapse. | 1 |
| Allow relapses in confirmation interval | PIRA confirmation assessment must not lie within a RAW window. This requirement is always enforced by our algorithm. Whether relapses between event and confirmation are allowed is not specified in ^1^, however a stricter version is recommended where relapses between the reference assessment and the last confirmation assessment + 30 days are not allowed. Our algorithm does not allow excluding relapses between reference and event directly, but this requirement is implicitly satisfied: if a relapse happens between a reference and event, it **either** leaves the score unchanged (post-relapse re-baselining assessment score ≤ previous reference), so a contribution of the relapse to later events can be excluded, **or** it leads to residual disability and thus a new reference after the relapse, thus the relapse is not between reference and event anymore. As for relapses between event and confirmation, “all” confirmation does by default not allow them, while “last” can optionally allow them. Since confirmation inclusion (“all” or “last”) is not specified, we tested **“all” with relapses not allowed**, **“last” with relapses allowed**, and **“last” with relapses not allowed**. | 2^*^ |
| Minimal distance | Not mentioned/discussed, we tested **no minimal distance requirement**. | 1 |
| Total tested options |  | **360** |

**eTable 27**: Tested PIRA/RAW definition options. * Allowing relapses during the confirmation interval for PIRA is only allowed if confirmation is required and if only the last value in the confirmation interval is considered.

### Differences between our algorithm and the harmonized definition

Our implementation of a confirmed roving reference differs from the implementation of the harmonized PIRA definition ^1^. In our algorithm the score that sets the new reference is the first score that marks an improvement, not its confirmation score. In Supplementary Figure 3 in the original publication, assessments 3, 7, and 15 are highlighted as new reference since they confirm the improvements at steps 2, 6, and 14. In our algorithm, assessments 2, 6, and 14 would be considered the roving baseline. This does not lead to a different value for the reference, but affects the reference’s timestamp and consequently the minimal distance to reference condition (not discussed in the harmonized PIRA definition). It is further not specified how exactly confirmation of the roving reference works (condition, confirmed reference score).

In addition, our implementation of a confirmed event score differs from the harmonized PIRA definition. In our algorithm the event score is the minimum of the event candidate’s score and all its confirmation scores. In Figure 3 in the original publication, the assessment at step 5 is a confirmed event with an event score 2.5 even though the confirmation scores at visits 6 to 8 are lower (2.0). In our algorithm, the confirmed event score would be 2.0, and the reference would be reset at step 5 (post-event re-baselining).

The harmonized definition also does not provide criteria for RAW and consequently also does not cover events that are not unambiguously classifiable as either RAW or PIRA.

## Results

See main manuscript for cohort details. Note that our cohort satisfies the recommendation of the harmonized PIRA definition that intervals between visits should not exceed 12 months.

### Event rate and event type contribution ranges

**Overall event rates** ranged from 18.4% to 31.2% with a mean event rate of 26.0%. Of the follow-ups with events, on average 71.5% had only one event, 20.3% had two, and 8.2% had three or more events. The highest number of events for one follow-up was 6.


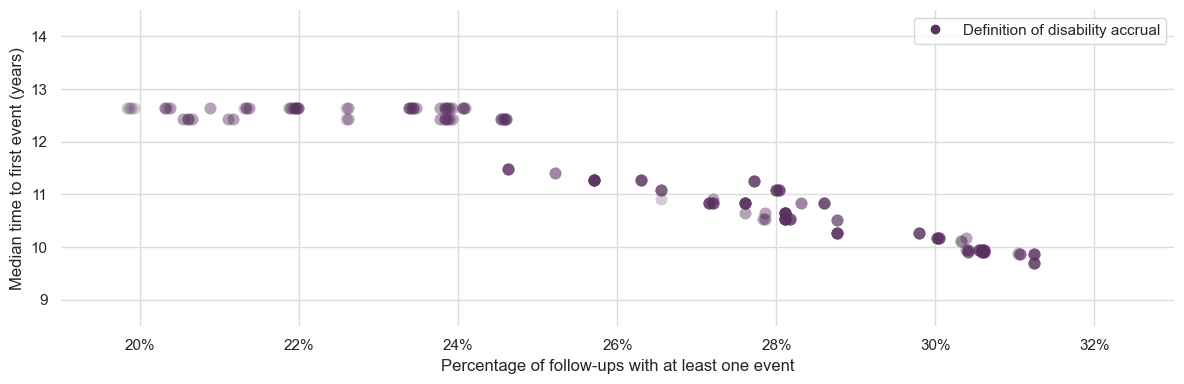


**eFigure 25**: Overall event rates (fraction of follow-ups with at least one event irrespective of type) vs. median time to first event (in years) for 204 definitions of RAW and PIRA (156 definitions are not shown since the median time to first event could not be determined). Each dot represents a definition.

**PIRA** event rates ranged from 12.3% to 23.4% with a mean event rate of 19.1%, and **RAW** event rates ranged from 3.8% to 6.5% with a mean event rate of 5.4% (eFigure 26A, eTable 28).

|  | Event rate (%) | | | Contrib. to events (%) | | | Contrib. to ΔEDSS (%) | | |
| --- | --- | --- | --- | --- | --- | --- | --- | --- | --- |
|  | Min | Mean | Max | Min | Mean | Max | Min | Mean | Max |
| Disability accrual (overall) | **18.4** | **26.0** | **31.2** | **-** | **-** | **-** | **-** | **-** | **-** |
| PIRA | 12.3 | 19.1 | 23.4 | 54.5 | 69.4 | 79.5 | 53.2 | 68.1 | 77.9 |
| PIRA with relapse during conf. | 1.1 | 2.2 | 3.9 | 2.9 | 7.3 | 20.2 | 2.9 | 7.0 | 19.7 |
| RAW | 3.8 | 5.4 | 6.5 | 13.1 | 15.6 | 18.0 | 14.1 | 16.8 | 19.5 |
| Undefined | 0.0 | 2.8 | 4.8 | 0.0 | 7.7 | 14.7 | 0.0 | 8.1 | 15.2 |

**eTable 28**: Event rate, event type contribution to the total number of events and event type contribution to the total ΔEDSS, overall and by event type for 360 definitions of EDSS disability accrual. **Abbreviations**: Contrib.: contribution.

**Overall**, between 880 and 1,584 (median 1,320.5) events of any type and total EDSS score increases from 1,037.0 to 1,935.5 (median 1,614.0) points were observed. Of all events, between 54.5% and 79.5% (mean 69.4%) were **PIRA**, and 13.1% to 18.0% (mean 15.6%) were **RAW** (eFigure 26B, eTable 28). Of the total EDSS score increase, 53.2% to 77.9% (mean 68.1%) was due to **PIRA**, and 14.1% to 19.5% (mean 16.8%) was due to **RAW** (eFigure 26C, eTable 28).


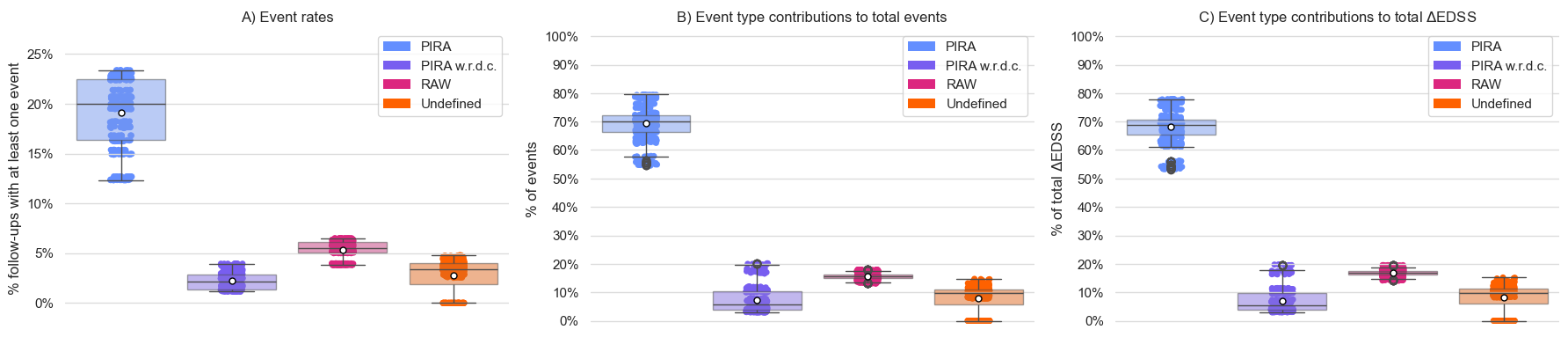


**eFigure 26**: Event rates and contributions by event type for 360 definitions of disability accrual. **A)** Event rates (fraction of follow-ups with at least one event of a given type). **B)** Event type contribution (number of events of a given type over total number of events). **C)** Event type contribution to the total ΔEDSS.

**PIRA** was the only event type in 11.0% to 21.2% (mean 16.8%) of all follow-ups, and **RAW** was the only event type in 2.3% to 4.5% (mean 3.5%) of all follow-ups. Of the follow-ups with events, 53.1% to 74.1% (mean 64.3%) had **PIRA** only, 11.0% to 16.2% (mean 13.4%) had **RAW** only, and 0% to 11.4% (mean 6.4%) only had undefined worsening (eTable 29).

|  | Fraction of follow-ups (%) | | | Of follow-ups with events (%) | | |
| --- | --- | --- | --- | --- | --- | --- |
|  | Min | Mean | Max | Min | Mean | Max |
| PIRA only | 11.0 | 16.8 | 21.2 | 53.1 | 64.3 | 74.1 |
| PIRA with relapse during confirmation only | 0.5 | 1.0 | 2.8 | 1.5 | 4.3 | 14.8 |
| RAW only | 2.3 | 3.5 | 4.5 | 11.0 | 13.4 | 16.2 |
| Undefined only | 0.0 | 1.7 | 2.9 | 0.0 | 6.4 | 11.4 |

**eTable 29**: Fraction of follow-ups where a given event type is the single contributor to disability accrual (left) and fraction of follow-ups with events where a given event type is the single contributor to disability accrual (right).

### Effect of the options for undefined worsening

Ignoring events at post-relapse re-baselining assessments significantly decreased the mean **overall** event rate (follow-ups with at least one event irrespective of type) from 26.2% with the “re-baselining only” option to 24.3% with the “never” option (p < 0.001). The “end” and “all” options (that have equal overall event rates by design) increase the mean event rate from 26.2% with the “re-baselining only” option to 26.7%, but this increase is not statistically significant (p = 0.285). Event rates **by type** do not change significantly, except for undefined worsening, which is ignored with the “never” option (eTable 30).

|  | Mean event rate (%) | | | | p-value vs. RB only | | |
| --- | --- | --- | --- | --- | --- | --- | --- |
|  | RB only | Never | End | All | Never | End | All |
| Disability accrual (overall) | **26.2** | **24.3** | **26.7** | **26.7** | **<0.001** | **0.285** | **0.285** |
| PIRA | 19.1 | 19.1 | 19.1 | 19.0 | - | - | 0.877 |
| PIRA confirmed in RAW window | 2.2 | 2.2 | 2.2 | 2.2 | - | - | 0.609 |
| RAW | 5.4 | 5.4 | 5.4 | 5.3 | - | - | 0.758 |
| Undefined | 3.1 | 0.0 | 3.8 | 4.3 | <0.001 | <0.001 | <0.001 |

**eTable 30**: Mean event rates for the four options for undefined worsening, and p-values (permutation test) for the comparison of the “never”, “all”, and “end” options to the default “re-baselining only” option (90 comparisons each). **Abbreviations**: RB only: re-baselining only. A “-” indicates no difference.


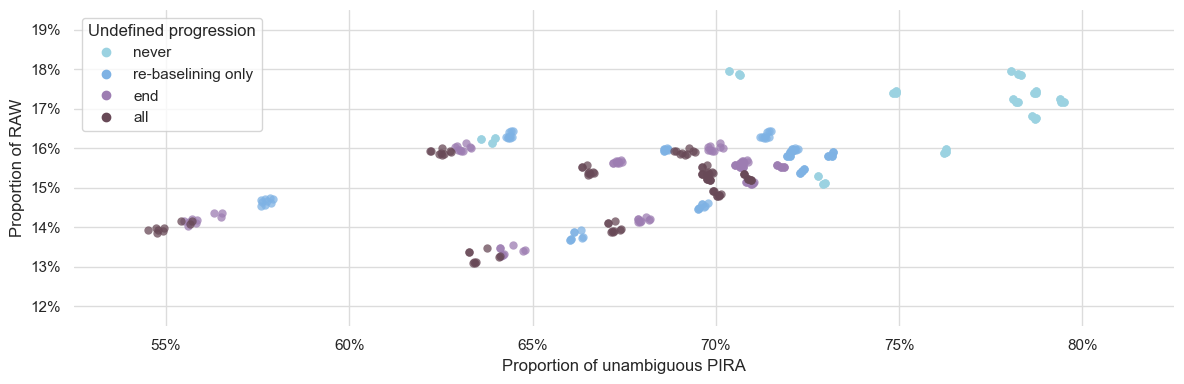


**eFigure 27**: Contribution of PIRA (x-axis) and RAW (y-axis) to the total number of events, 360 definitions. The options for undefined worsening are color coded.

Ignoring events at post-relapse re-baselining assessments significantly increased PIRA (from 68.7% to 75.2%, p < 0.001) and RAW (from 15.5% to 16.9%, p < 0.001) contributions to the total event count compared to the default re-baselining only mode, and using the “all” option significantly reduces PIRA and RAW contributions while increasing the contribution of undefined worsening (eTable 31, eFigure 27, eFigure 28).


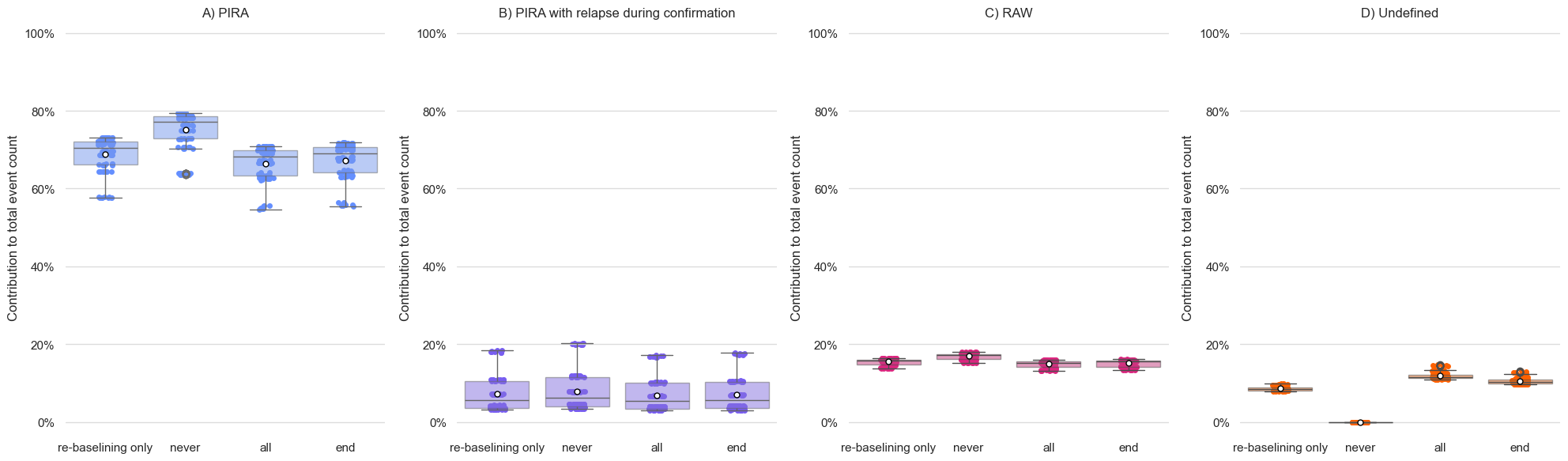


**eFigure 28**: Contributions of each event type to the overall event count by option for undefined worsening, 360 definitions each. **A)** PIRA contribution. **B)** PIRA with relapse during confirmation. **C)** RAW contribution. **D)** Undefined contribution.

|  | Mean event contribution (%) | | | | p-value when compared to RB only | | |
| --- | --- | --- | --- | --- | --- | --- | --- |
|  | RB only | Never | End | All | Never | End | All |
| PIRA | 68.7 | 75.2 | 67.3 | 66.4 | <0.001 | 0.034 | 0.001 |
| PIRA with relapse during conf. | 7.2 | 7.9 | 7.1 | 6.8 | 0.323 | 0.802 | 0.529 |
| RAW | 15.5 | 16.9 | 15.1 | 14.9 | <0.001 | 0.011 | <0.001 |
| Undefined | 8.6 | 0.0 | 10.5 | 11.8 | <0.001 | <0.001 | <0.001 |

**eTable 31**: Event type contributions by undefined worsening annotation mode, 90 definitions per mode.

Requiring a potential undefined event score to have a greater or equal or a greater score than the current RAW/PIRA baseline does not have a large impact on event rates or event type contributions. The mean overall event rates are 26.6%, 26.5%, and 26.5% for the “any”, “equal or greater”, and “greater only” options, respectively (90 definitions each; the “never” option for undefined worsening excluded because only undefined events are affected by this threshold choice). The proportion of follow-ups with at least one undefined event decreased from 3.7% with the “any” option to 3.6% with the “greater only” option (p = 0.266, 90 comparisons, “never” for undefined excluded), while the rates for the other event types remain unchanged (eTable 32). Changes to the event type contributions are small and not statistically significant (eTable 33).

|  | Mean event rate (%) | | | p-value vs. “any” | |
| --- | --- | --- | --- | --- | --- |
|  | Any | Equal or greater | Greater only | Equal or greater | Greater only |
| PIRA | 19.1 | 19.1 | 19.1 | - | - |
| PIRA with relapse during conf. | 2.2 | 2.2 | 2.2 | - | - |
| RAW | 5.4 | 5.4 | 5.4 | - | - |
| Undefined | 3.7 | 3.7 | 3.6 | 0.880 | 0.266 |

**eTable 32**: Event rates by undefined worsening threshold mode, 90 definitions per mode. A “-” indicates no difference.

|  | Mean event type contributions (%) | | | p-value vs. “any” | |
| --- | --- | --- | --- | --- | --- |
|  | Any | Equal or greater | Greater only | Equal or greater | Greater only |
| PIRA | 67.4 | 67.4 | 67.6 | 0.976 | 0.769 |
| PIRA with relapse during conf. | 7.0 | 7.0 | 7.1 | 0.985 | 0.950 |
| RAW | 15.2 | 15.2 | 15.2 | 0.956 | 0.706 |
| Undefined | 10.4 | 10.4 | 10.1 | 0.839 | 0.197 |

**eTable 33**: Event type contributions by undefined worsening threshold mode, 90 definitions per mode.

### Effect of roving reference confirmation options

Requiring 90 days “last” confirmation for the roving baseline decreased the mean overall event rate from 26.4% to 25.8% (p = 0.194), and requiring 90 days “all” confirmation reduced the mean overall event rate to 25.8% (p = 0.177 vs. next-confirmed). When requiring 90 days confirmation, the overall event rate is 25.8% for both the “all” and the “last” requirement (“all” vs. “last” p = 0.968). Changes to mean event rates by type are also not statistically significant (eTable 34, eFigure 29).

|  | Mean event rate (%) | | | p vs. next-confirmed | |
| --- | --- | --- | --- | --- | --- |
|  | Next-conf. | 90 days last | 90 days all | 90 days last | 90 days all |
| Disability accrual (overall) | **26.4** | **25.8** | **25.8** | **0.194** | **0.177** |
| PIRA | 19.5 | 18.9 | 18.9 | 0.218 | 0.198 |
| PIRA with relapse during conf. | 2.3 | 2.2 | 2.2 | 0.542 | 0.416 |
| RAW | 5.5 | 5.3 | 5.3 | 0.224 | 0.190 |
| Undefined | 2.8 | 2.7 | 2.7 | 0.772 | 0.748 |

**eTable 34**: Mean event rates by roving reference confirmation mode, 120 definitions per mode.


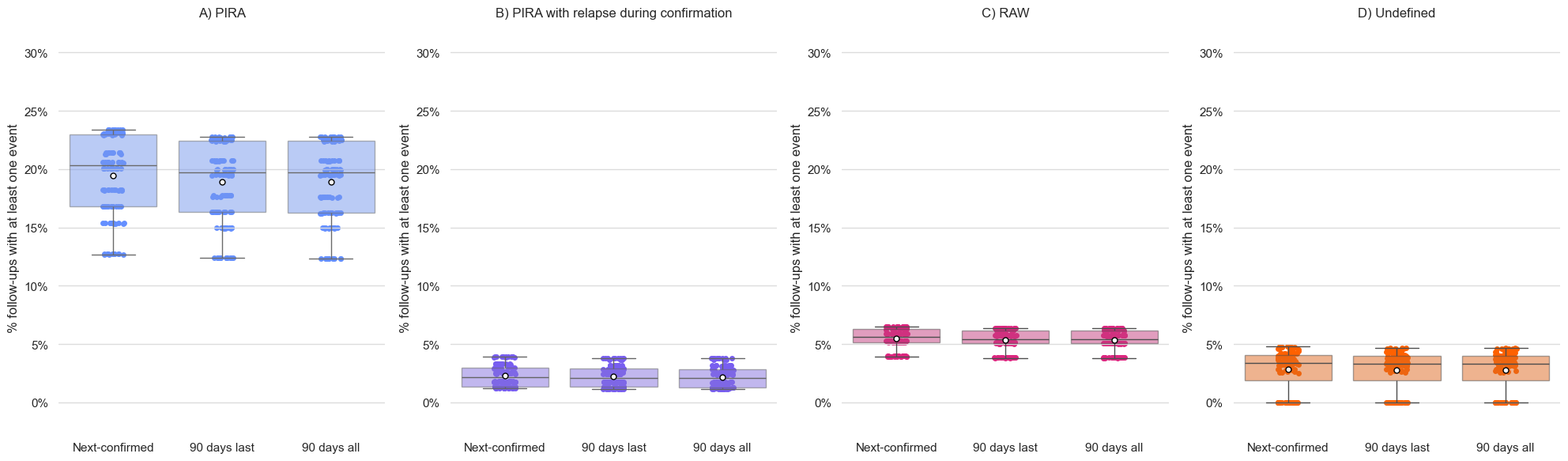


**eFigure 29**: Event rates (fraction of follow-ups with at least one event of a given type) by option for the roving reference confirmation (next-confirmed, 90 days “last” confirmed, 90 days “all” confirmed), 120 definitions each. **A)** PIRA. **B)** PIRA confirmed in RAW window. **C)** RAW. **D)** Undefined.

Changes to event type contributions (eTable 35) were also not statistically significant.

|  | Mean event type contribution (%) | | | p vs. next-confirmed | |
| --- | --- | --- | --- | --- | --- |
|  | Next-conf. | 90 days last | 90 days all | 90 days last | 90 days all |
| PIRA | 69.3 | 69.4 | 69.4 | 0.909 | 0.896 |
| PIRA with relapse during conf. | 7.3 | 7.3 | 7.2 | 0.957 | 0.883 |
| RAW | 15.7 | 15.6 | 15.6 | 0.470 | 0.617 |
| Undefined | 7.7 | 7.8 | 7.8 | 0.913 | 0.894 |

**eTable 35**: Mean event rates by roving reference confirmation mode, 120 definitions per mode.

### Effect of confirmation options

Overall event rates ranged from 20.0% when requiring sustained over ≥ 360 days to 30.0% when requiring 90 days “last” confirmation (eFigure 30, eTable 36).


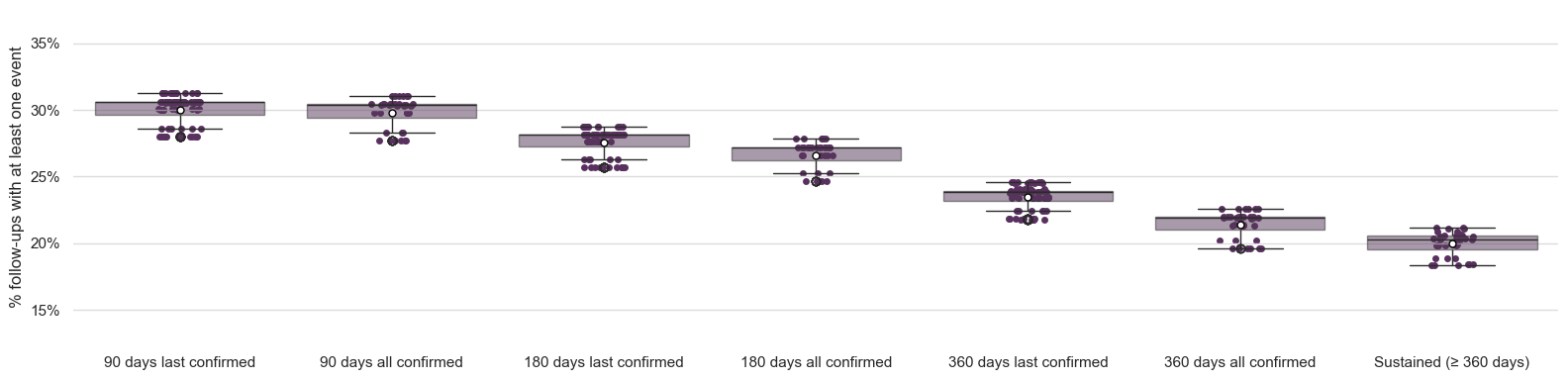


**eFigure 30**: Overall event rates for the 7 analyzed confirmation options.

**PIRA** event rates ranged from 12.5% when requiring sustained over ≥ 360 days to 22.8% when requiring 90 days “last” confirmation (eFigure 31A, eTable 36), and **RAW** rates ranged from 3.9% when requiring sustained over ≥ 360 days to 6.4% when requiring 90 days “last” confirmation (eFigure 31C, eTable 36).

|  | Mean event rate (%) | | | | | | |
| --- | --- | --- | --- | --- | --- | --- | --- |
|  | 90 d last | 90 d all | 180 d last | 180 d all | 360 d last | 360 d all | Sus ≥ 360 d |
| Overall | **30.0** | **29.8** | **27.6** | **26.6** | **23.5** | **21.4** | **20.0** |
| PIRA | 22.8 | 22.6 | 20.6 | 19.7 | 17.2 | 15.1 | 12.5 |
| PIRA rel. conf. | 1.5 | 1.7 | 2.0 | 2.5 | 2.2 | 2.9 | 3.8 |
| RAW | 6.4 | 6.2 | 5.8 | 5.1 | 5.2 | 3.9 | 3.9 |
| Undefined | 3.0 | 3.0 | 2.9 | 2.9 | 2.5 | 2.5 | 2.5 |

**eTable 36**: Mean event rates for the 7 analyzed confirmation options. **Abbreviations**: d: days, Sus: sustained.


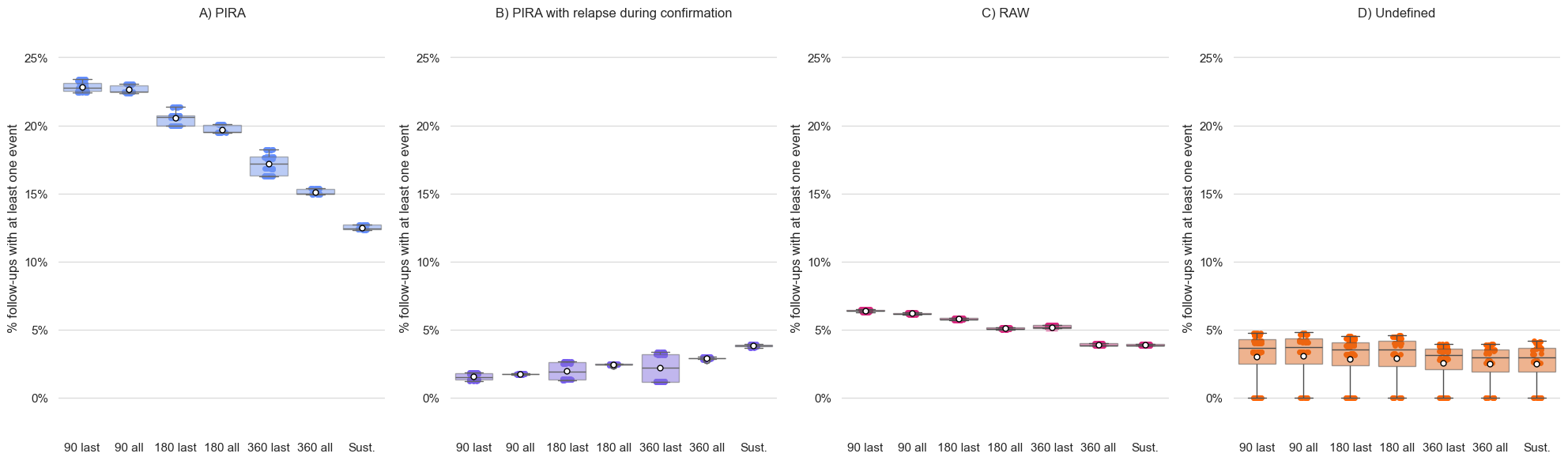


**eFigure 31**: Event rates by confirmation option. **A)** PIRA. **B)** PIRA with relapse during confirmation. **C)** RAW. **D)** Undefined. **Abbreviations**: 90 last: 90 days last-confirmed, 90 all: 90 days all-confirmed, 180 last: 180 days last-confirmed, 180 all: 180 days last-confirmed, 360 last: 360 days last-confirmed, 360 all: 360 days all-confirmed, Sust.: sustained over ≥ 360 days.

**PIRA** event type contributions ranged from 58.1% when requiring sustained over ≥ 360 days to 73.2% when requiring 90 days “last” confirmation (eFigure 32A, eTable 37). The contribution of **PIRA with relapse during confirmation** increased by almost a factor of 5 when going from 90 days last-confirmed (3.7%) to sustained over ≥ 360 days (18.1%) (eFigure 32B, eTable 37).

|  | Mean event type contribution (%) | | | | | | |
| --- | --- | --- | --- | --- | --- | --- | --- |
|  | 90 d last | 90 d all | 180 d last | 180 d all | 360 d last | 360 d all | Sus ≥ 360 d |
| PIRA | 73.2 | 73.0 | 71.1 | 70.3 | 68.7 | 66.7 | 58.1 |
| PIRA rel. conf. | 3.7 | 4.2 | 5.3 | 7.2 | 7.1 | 10.9 | 18.1 |
| RAW | 16.0 | 15.5 | 16.1 | 14.7 | 16.5 | 13.9 | 14.8 |
| Undefined | 7.1 | 7.3 | 7.5 | 7.9 | 7.7 | 8.5 | 9.0 |

**eTable 37**: Mean event rates for the 7 analyzed confirmation options. **Abbreviations**: d: days, Sust: sustained.


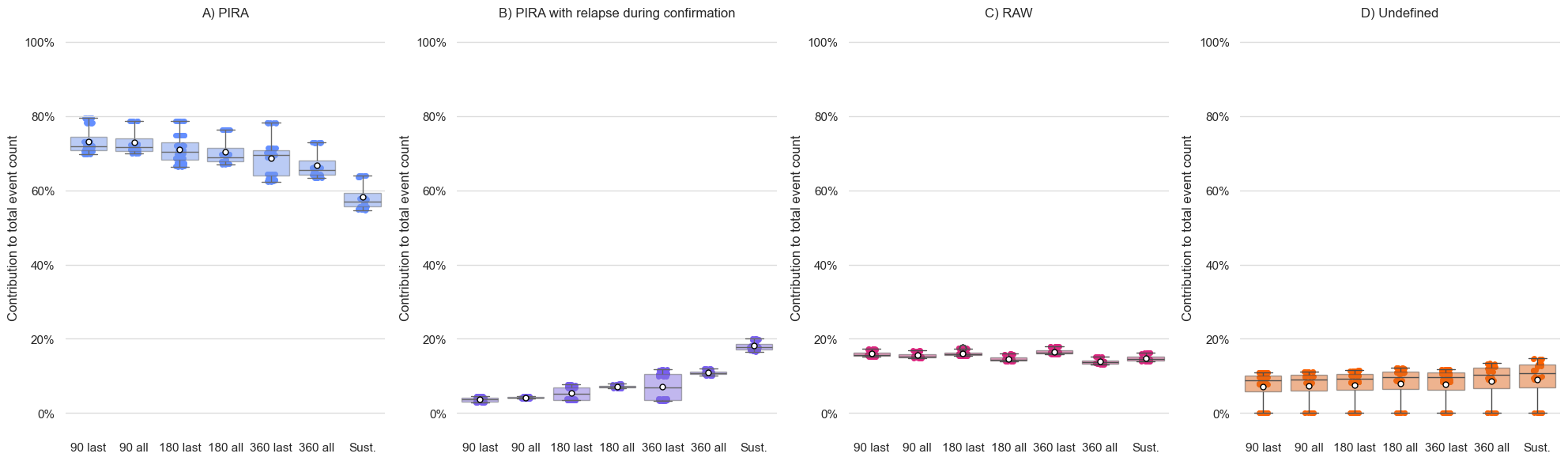


**eFigure 32**: Type contributions by confirmation option. **A)** PIRA. **B)** PIRA with relapse during confirmation. **C)** RAW. **D)** Undefined. **Abbrev**.: 90 last: 90 days last-confirmed, 90 all: 90 days all-confirmed, 180 last: 180 days last-confirmed, 180 all: 180 days last-confirmed, 360 last: 360 days last-confirmed, 360 all: 360 days all-confirmed, Sust.: sustained over ≥ 360 days.

### Relapses in confirmation interval

Allowing relapses in the confirmation interval when requiring “last” confirmation only affects event rates and event type contributions for PIRA and PIRA confirmed in RAW window. The rate of **PIRA with relapse during confirmation** decreased from 2.5% to 1.3% (p < 0.001) when allowing relapses, while the **PIRA** rate increased from 19.8% to 20.6% (p = 0.016) (eTable 38). The contribution of PIRA increased from 69.0% to 72.9% (p < 0.001) while the contribution of PIRA confirmed in RAW window decreased from 7.3% to 3.4% (p < 0.001) (eTable 38, eFigure 33).

|  | Mean event rate (%) | | p-value | Mean event type contribution (%) | | p-value |
| --- | --- | --- | --- | --- | --- | --- |
|  | Allow relapses | Don’t allow |  | Allow relapses | Don’t allow |  |
| PIRA | 20.6 | 19.8 | 0.016 | 72.9 | 69.0 | <0.001 |
| PIRA rel. conf. | 1.3 | 2.5 | <0.001 | 3.4 | 7.3 | <0.001 |
| RAW | 5.8 | 5.8 | - | 16.2 | 16.2 | - |
| Undefined | 2.8 | 2.8 | - | 7.4 | 7.4 | - |

**eTable 38**: Relapses in confirmation, last-confirmed only, 108 comparisons. A “-” indicates no difference.


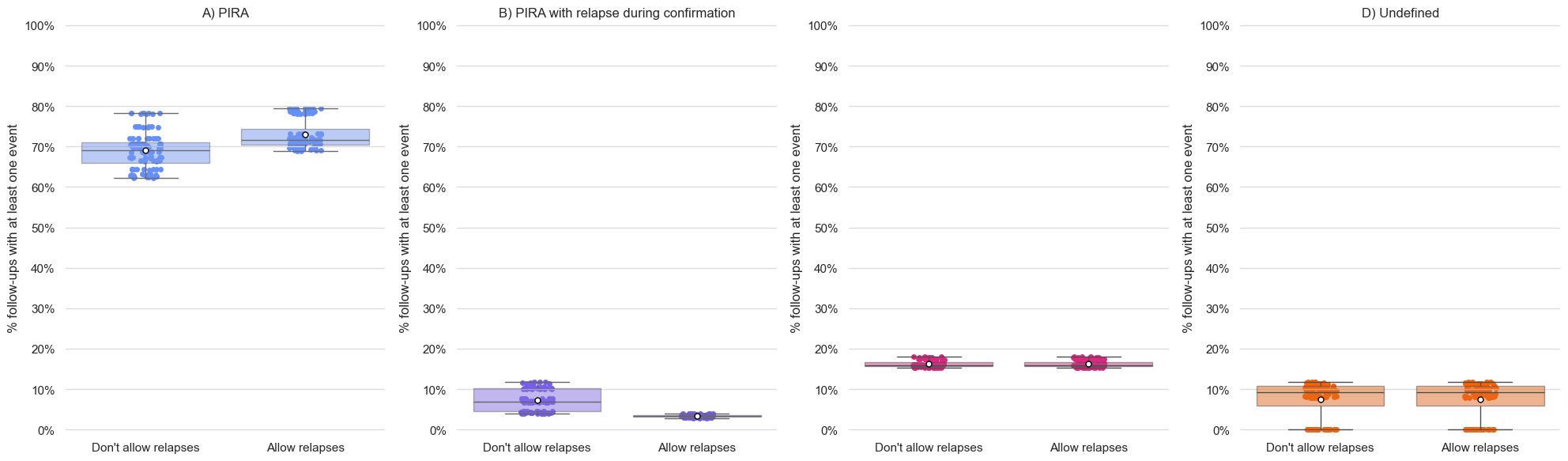


**eFigure 33**: Event type contributions by option to allow relapses within the confirmation interval for “last” confirmation, 108 definitions each. **A)** PIRA. **B)** PIRA confirmed in RAW window. **C)** RAW. **D)** Undefined.

### Example of a follow-up with a large variation in event type attribution


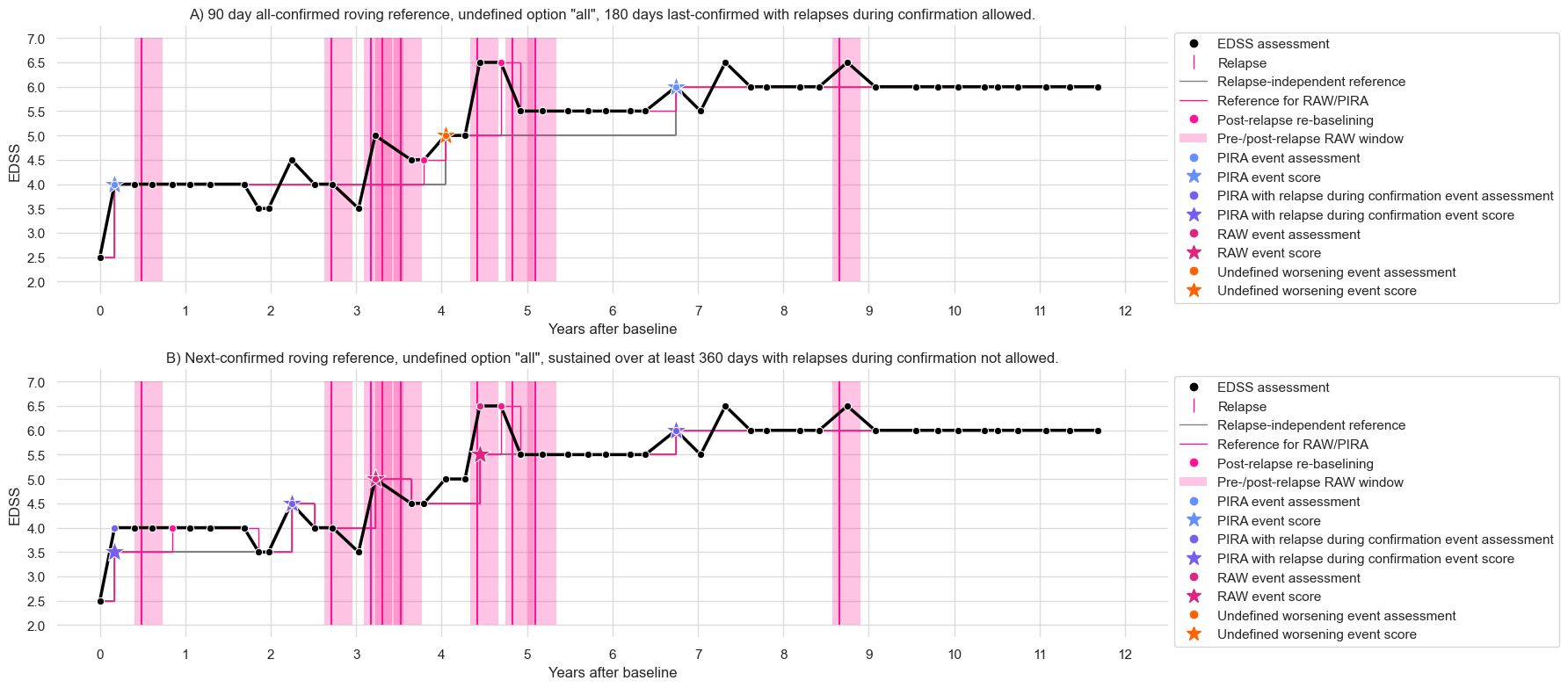


**eFigure 34**: RMS follow-up from AMSTR under two definitions compatible with the harmonized definition of PIRA. Undefined progression is always allowed (“all” option), RAW window 30 days pre- and 90 days post-relapse. **A)** 90 days all-confirmed roving reference, 180 days last-confirmed with relapses between event and confirmation allowed for PIRA. 2 PIRA events and 1 undefined event. **B)** Next-confirmed roving reference, sustained over at least 360 days, relapses during confirmation not allowed for PIRA. 3 PIRA with relapse during confirmation events, 2 RAW events.

# Analysis of the standardized PIRA definition

## Methods

The standardized definition of PIRA specifies the following criteria: A worsening must be compared with a reference re-baselined after each PIRA event, relapse, and EDSS improvement; it must not be preceded by relapses since the previous assessment; it must remain above the EDSS threshold for significant worsening throughout a confirmation period of at least 12 months; only EDSS scores not preceded by a relapse within 30 days are used for confirmation ^2^.

The standardized definition of PIRA differs from the previously published harmonized definition of PIRA in the minimal increase condition (+ 1.0 for references < 6, harmonized used < 5.5), different intervals pre- and post-relapse (“RAW window”), in the confirmation criteria for PIRA, and the criterion that no relapses must be present between previous score and event (optional for the harmonized definition) ^1^ .

Our algorithm identifies PIRA events alongside RAW events and the additional categories “PIRA with relapse during confirmation” and “undefined worsening” for ambiguous events. Therefore, there are parameters covering disability accrual event detection and classification that are not explicitly mentioned (or not required) for the standardized PIRA definition ^2^. Our algorithm also includes definition aspects that are not directly required for PIRA definition, but that affect relative PIRA event type contributions (see eTable 39). In addition, we chose a different implementation for the confirmation of improvements (roving reference), for the handling of relapses prior to potential PIRA candidates, and for the handling of relapses within the confirmation interval of a PIRA candidate (see the following subsections for a detailed description of the differences, and the Supplemental Methods for implementation details of our algorithm). eTable 39 describes the 144 parameter choices that reproduce the proposed standardized PIRA definition as closely as possible.

### Roving reference confirmation

Our implementation of a confirmed roving reference requires that the new reference candidate score and all confirmation scores are lower than the previous reference, and the new reference is set to the maximum of the candidate score and its confirmation scores (eFigure 35A). The re-baselining approach of the standardized PIRA definition defines an EDSS improvement as “any decrease of EDSS score that was confirmed with the same or a lower EDSS score” and sets the new reference “using the lowest EDSS score in the confirmation period” (eFigure 35B). We implemented the approach in a separate version of our algorithm to assess its impact on event rates and event type contributions (see Results), but did not include it in the main version.


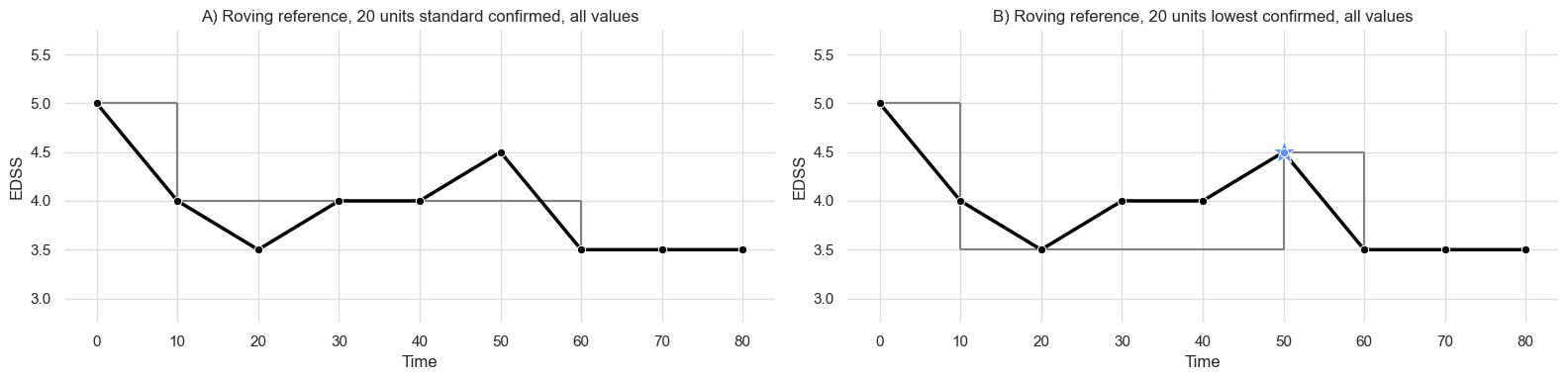


**eFigure 35**: **A)** Our implementation of roving reference confirmation, 20 units confirmation required. The improvement at step 10 is confirmed at steps 20 and 30, and the new reference is set to 4.0. **B)** Standardized PIRA implementation, 20 units confirmation required. The improvement at step 10 is confirmed at steps 20 and 30, and the new reference is the lowest confirmation score, i.e. 3.5.

The implementation using the lowest score can lead to the situation where the confirmation of the improvement is an event (eFigure 36B). Using the maximal candidate/confirmation score implementation prevents this (eFigure 36A).


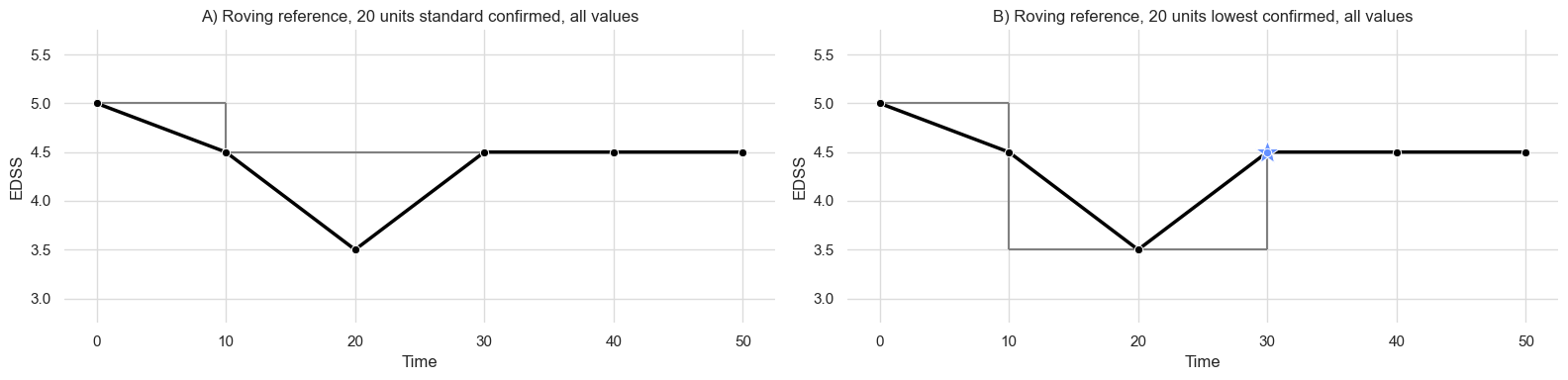


**eFigure 36**: **A)** Our implementation of roving reference confirmation, 20 units confirmation required. The improvement at step 10 is confirmed at steps 20 and 30, and the new reference is set to 4.5. **B)** Standardized PIRA implementation, 20 units confirmation required. The improvement at step 10 is confirmed at steps 20 and 30 (both scores are equal or lower than the improvement candidate), and the new reference is the lowest confirmation score, i.e. 3.5. Therefore, the increase at step 30 fulfills the minimum increase confirmation for an event.

Using the lowest confirmation scores also implicitly leads to inconsistent confirmation criteria and a large impact of minor EDSS fluctuations (eFigure 37).


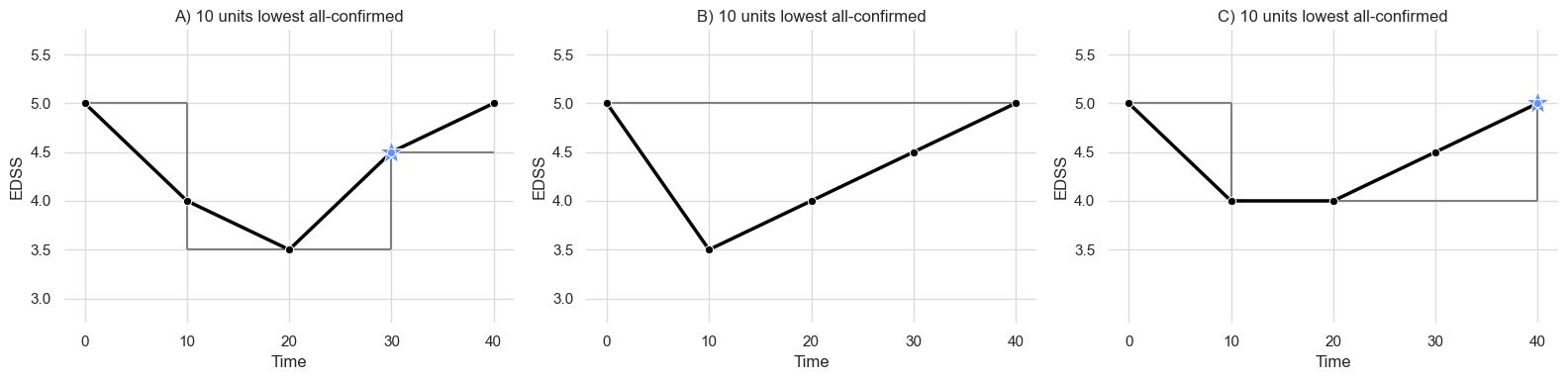


**eFigure 37**: Standardized PIRA implementation of roving reference confirmation, 10 units confirmation required. **A)** The improvement at step 10 is confirmed by the score at step 20, thus the baseline is reset to 3.5. Subsequently, the increase at step 30 fulfills the minimum increase criterion for PIRA. **B)** The improvement at step 10 is not followed by the same or a lower score, therefore it is not considered as new reference. The same holds for steps 20 and 30, which are also lower than the previous baseline, but not followed by an equal or lower score. Note that the difference between PIRA in A) and no PIRA in B) is caused by switching the scores at steps 10 and 20. **C)** The improvement at step 10 is confirmed by the score at step 20, thus the baseline is reset to 4.0, leading to an event at step 40. Note that the earlier detection of the event in A) compared to C) is due to an *unconfirmed* fluctuation at step 20. With our confirmation implementation, the reference would be set to 4.0 at step 10 in all three examples, leading to an event at step 40.

### Relapses prior to PIRA candidate events

The standardized PIRA definition requires the absence of relapses between the event candidate and the previous assessment in order to exclude a contribution of the relapse to the disability accrual. Our implementation ensures this by re-baselining after a relapse if it leads to a higher score and by classifying events at such post-relapse re-baselining assessments as *undefined worsening* (see main manuscript and Supplemental Methods). Therefore, if a relapse is present between event and candidate, the candidate is either still in the RAW window of the relapse and thus a RAW candidate, or after the RAW window, and thus an undefined event candidate (eFigure 38A, eFigure 38B). The optional condition that no relapses must be present between reference and event candidate (in order to exclude a contribution of the relapse) is also implicitly covered by our re-baselining implementation: if a relapse occurs, the score at the post-relapse re-baselining assessment is either equal or lower than the previous reference, and thus the relapse does not contribute to subsequent potential increases, or higher than the previous baseline, in which case the baseline is reset to this new higher score, and all subsequent scores are then compared to this new reference (eFigure 38C, eFigure 38D). We did not implement the no relapse between reference/previous and event candidate condition since it leads to a loss of sensitivity with respect to PIRA and would still require additional definitions for the other event types (eFigure 38A, eFigure 38B).


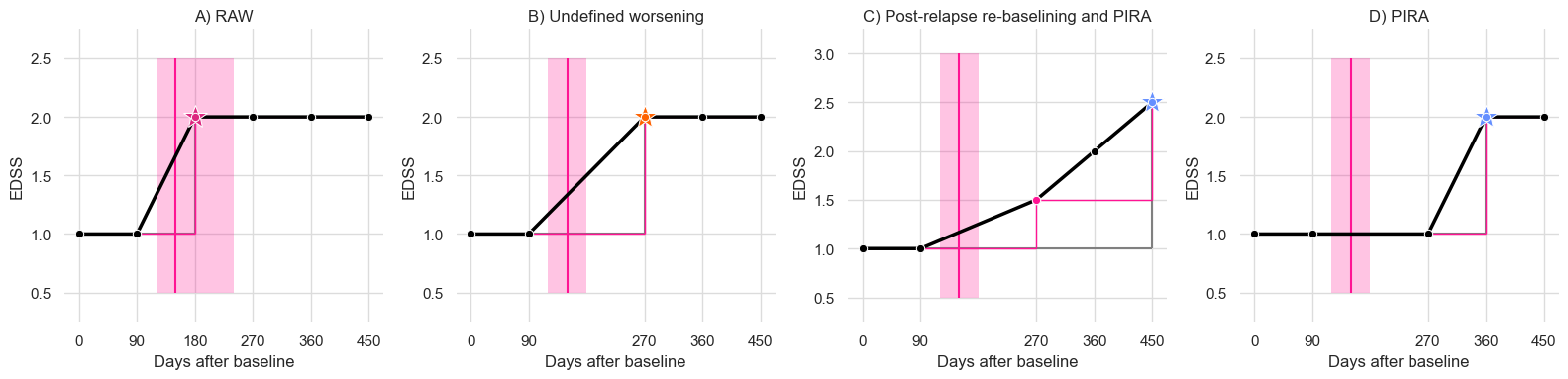


**eFigure 38**: Relapses between previous assessment and event candidate. **A)** The event candidate is within the RAW window, thus it is classified as RAW. **B)** The event candidate is outside the RAW window and thus at a post-relapse re-baselining assessment. It is classified as undefined worsening. **C)** The relapse leads to residual disability. This increase does not satisfy the minimal increase condition, thus it is not an undefined event. The baseline is reset to 1.5, and any subsequent increase is compared to this baseline. The increase at day 450 with respect to the new baseline at day 270 is therefore unambiguously independent of relapse activity. **D)** The score does not change after the relapse, thus the baseline remains unchanged and a contribution of the relapse can be excluded. Using the no relapse between previous score and event candidate would classify A) and B) as “not PIRA”, without information on whether it is RAW.

### Relapses in the confirmation interval of PIRA events

The standardized PIRA definition requires that “no relapses must occur in the 30 days before the confirmation score” and that “confirmation scores not fitting these criteria were disregarded”. We implemented this approach in a separate version of our algorithm to assess its impact on event rates and event type contributions (see Results), but did not include it in the main version. This confirmation strategy might lead to the confirmation of a PIRA event even though relapses are present within the confirmation period if there is at least one score that satisfies the confirmation distance criterion and is not in proximity of a relapse (eFigure 39).


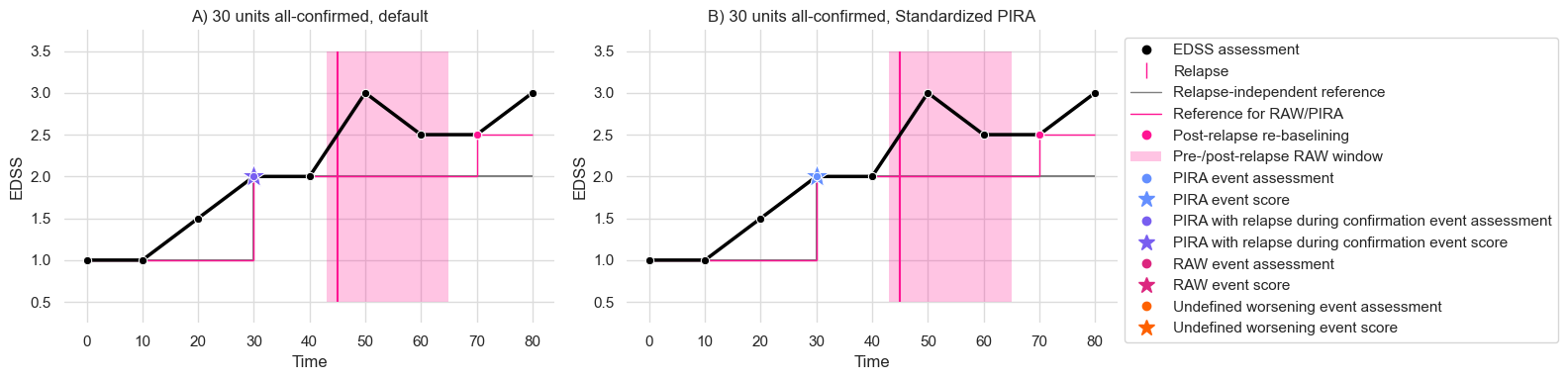


**eFigure 39**: **A)** Our implementation of PIRA confirmation, 30 units confirmation required. The scores at steps 40, 50, and 60 are the confirmation scores for the increase at step 30. Since there is a relapse within this period, the event is classified as *PIRA with relapse during confirmation*. **B)** Standardized PIRA definition. The confirmation scores at steps 50 and 60 are in proximity of a relapse and thus disregarded. The score at step 40 and the first score that then satisfies the minimal confirmation distance requirement (step 70) fulfill the minimal increase condition, thus the event is confirmed as PIRA.

Ignoring confirmation assessments might lead to event confirmation in the presence of recovery and subsequent increase (eFigure 40) and to event non-confirmation in the presence of recovery after the confirmation period if a relapse is present (eFigure 41).


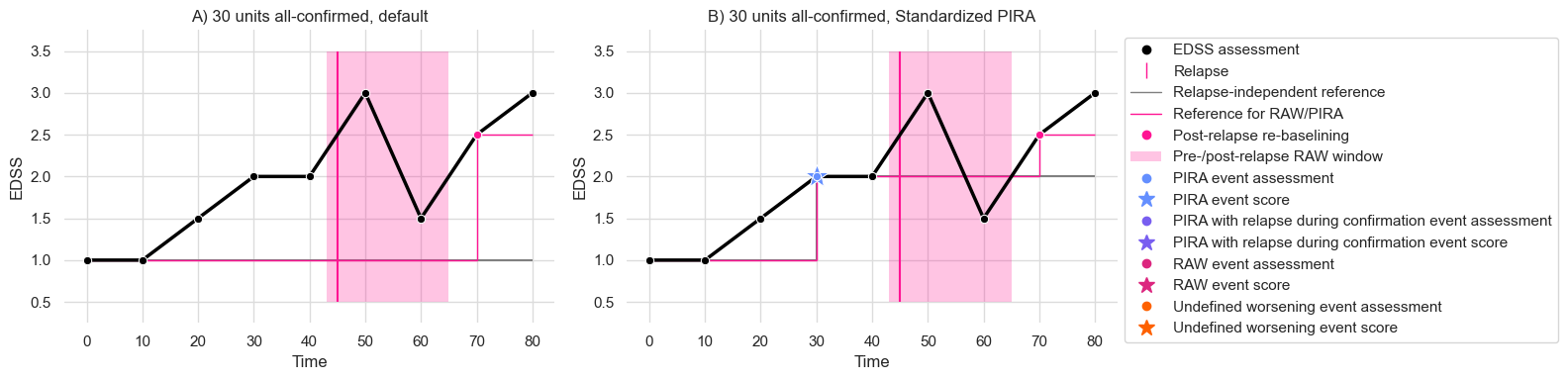


**eFigure 40**: **A)** Our implementation of PIRA confirmation, 30 units confirmation required. The scores at steps 40, 50, and 60 are the confirmation scores for the increase at step 30. Since the score at step 60 does not satisfy the minimal increase condition, the increase is unconfirmed. **B)** Standardized PIRA definition. The confirmation scores at steps 50 and 60 are in proximity of a relapse and thus disregarded. The score at step 40 and first score that then satisfies the minimal confirmation distance requirement (step 70) fulfill the minimal increase condition, thus the event is confirmed as PIRA.


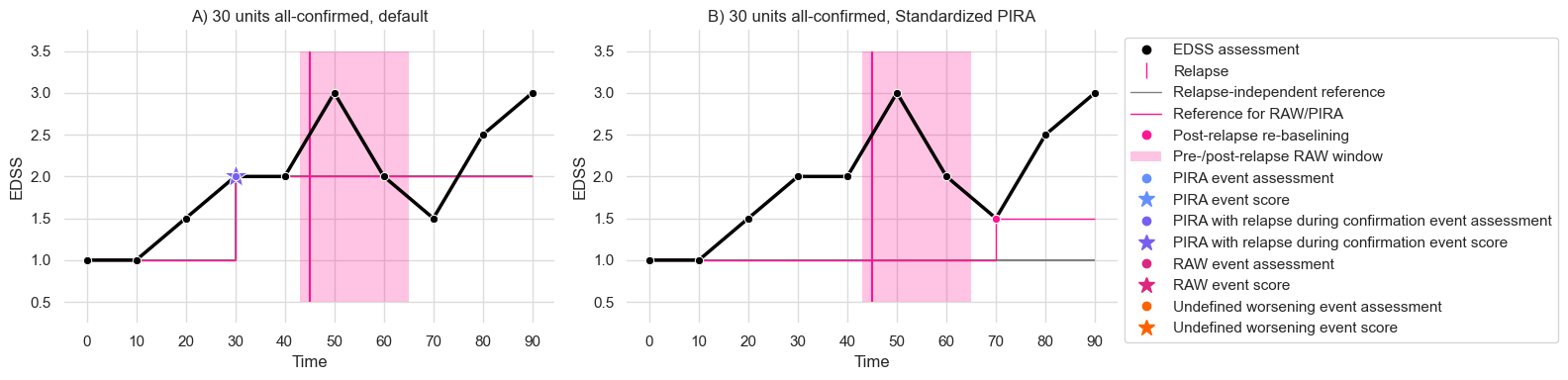


**eFigure 41**: **A)** Our implementation of PIRA confirmation, 30 units confirmation required. The scores at steps 40, 50, and 60 are the confirmation scores for the increase at step 30. Since there is a relapse within this period, the event is classified as *PIRA with relapse during confirmation*. **B)** Standardized PIRA definition. The confirmation scores at steps 50 and 60 are in proximity of a relapse and thus disregarded. The first score that then satisfies the minimal confirmation distance requirement (step 70) does not fulfill the minimal increase condition, thus the event is not confirmed, even though there is an assessment that satisfies the minimal confirmation distance condition and all assessments within the specified confirmation period of 30 days fulfill the minimum increase condition.

Events may be left unconfirmed if no confirmation scores outside the relapse window are present, even if there are assessments that satisfy the minimal confirmation distance (eFigure 42).


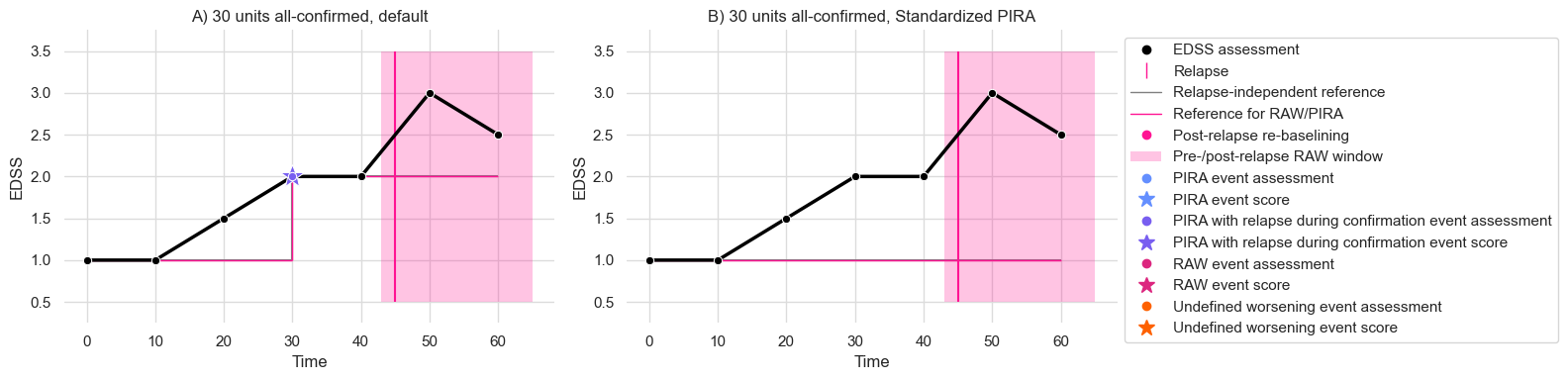


**eFigure 42**: **A)** Our implementation of PIRA confirmation, 30 units confirmation required. The scores at steps 40, 50, and 60 are the confirmation scores for the increase at step 30. Since there is a relapse within this period, the event is classified as *PIRA with relapse during confirmation*. **B)** Standardized PIRA definition. The confirmation scores at steps 50 and 60 are in proximity of a relapse and thus disregarded. There is no score that satisfies the minimal confirmation distance requirement, thus the event is not confirmed, even though there is an assessment that satisfies the minimal confirmation distance condition and all assessments within the specified confirmation period of 30 days fulfill the minimum increase condition.

In contrast to our implementation, where the RAW window size for event categorization and confirmation is equal (only one – clinically informed – definition of what “proximity to relapse” means), the standard PIRA definition allows different window sizes for confirmation (eFigure 43).


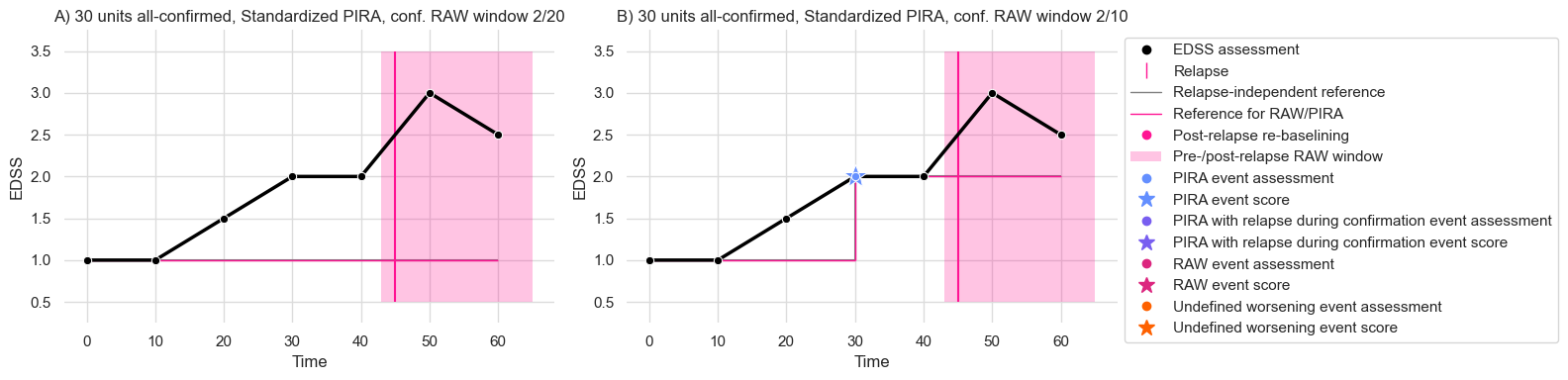


**eFigure 43**: Standardized PIRA definition. **A)** Confirmation RAW window 2 units pre-/20 units post-relapse (same as for event categorization. No confirmation scores that satisfy the minimal confirmation distance condition, thus unconfirmed. **B)** Confirmation RAW window 2 units pre-/10 units post-relapse. The score at step 60 is thus not in proximity of a relapse by confirmation RAW window size standards, and thus the event is confirmed. **Abbreviations**: conf.: confirmation.

### Tested options

| Option | Parameter choice for reproducing Müller et al. 2025 | N tested |
| --- | --- | --- |
| Event merging | Not mentioned/discussed. We assume that there is no merging, thus chose **False** | 1 |
| Undefined events | Not mentioned/discussed. We propose using **re-baselining only**, but tested all four options. | 4 |
| Constraints for undefined | Not mentioned/discussed. We propose using **unconstrained**, but tested all three options. | 3 |
| Baseline | For the harmonized definition, a new reference due to improvement must be lower than the previous reference, and confirmation scores must be equal to or lower than the new reference candidate. The new reference is then the minimum of all confirmation scores. In our implementation, a new reference and its confirmation scores must be lower than the previous reference, and the new reference is the maximum of the candidate and confirmation scores. We tested both our proposed (“all”) and the standardized definition’s version for the roving reference (improvement) with ≥ 3 months (90 days) confirmation. | 2 |
| Minimal increase | + 1.5 for reference 0, + 1.0 for reference < 6.0, + 0.5 else | 1 |
| Confirmation | The reference definition requires confirmation over ≥ 12 months, where all scores (except those close to relapses, see “allow relapses”) must fulfill the confirmation condition. We tested **12 months** (360 days) **“all”** confirmed. | 1 |
| Confirmation type | **Minimum**. | 1 |
| Require confirmation for last visit | Not mentioned/discussed, we tested **True** | 1 |
| Left hand side confirmation tolerance | Not mentioned/discussed, we tested **0** (i.e. earliest confirmation assessment for confirmation at x days is at day x) | 1 |
| Right hand side confirmation tolerance | Not mentioned/discussed, we tested **∞** (i.e. confirmation assessment can be at any time after the minimal confirmation distance) | 1 |
| RAW window size | The proposed definition requires “no relapses between previous and worsening”. This option is not supported in our algorithm, but implicitly covered: if a relapse happens between the previous score and the candidate score, the candidate score is either still in proximity of the relapse (within the RAW window) and thus a RAW candidate, not a PIRA candidate, or it is after the RAW window. If it is after the RAW window, it is either equal or lower than the previous score, thus not an event candidate, or higher than the previous score and thus considered a post-relapse re-baselining assessment or undefined event and can therefore not be PIRA. This approach ensures that previous relapses do not contribute to PIRA. It also implicitly covers the criterion that no relapses must occur between reference and worsening (if the relapse leads to residual disability, the reference is reset, and if it does not lead to residual disability it does not contribute to potential later PIRA events). Since our algorithm requires the specification or a RAW window, we tested **0 days pre- and 90 days post-relapse**, **30 days pre- and 90 days post-relapse**, and **180 days pre-/post-relapse**, following the tested RAW window sizes for the analysis in ^2^. | 3 |
| Allow relapses in confirmation interval | According to the proposed standardized definition, no relapses must occur in the 30 days before each confirmation score, and confirmation scores not fitting these criteria were disregarded. We implemented this condition as an experimental extra option and compared it to our standard (recommended) implementation. Our implementation does not allow any relapses within the confirmation interval for “all” confirmed (irrespective of whether or not assessments in proximity to relapses are present), does not disregard any confirmation scores, and classifies events as *PIRA confirmed in the presence of relapses* if relapses are present. We tested our implementation with **all-confirmed and relapses not allowed**, and the **proposed definition with scores ≤ 30 days after a relapse discarded**. | 2 |
| Minimal distance | Not mentioned/discussed, we tested **no minimal distance requirement**. | 1 |
| Total tested options |  | **144** |

**eTable 39**: Tested disability accrual and RAW/PIRA definition options.

## Results

See main manuscript for cohort details. Note that our cohort satisfies the recommendation that intervals between visits should not exceed 12 months ^1^.

### Event rate and event type contribution ranges

**Overall event rates** (fraction of follow-ups with at least one event) ranged from 19.1% to 21.7% with a mean event rate of 20.9%. **PIRA** event rates ranged from 13.9% to 16.7% with a mean event rate of 15.3%, and **RAW** event rates (fraction of follow-ups with at least one RAW event) ranged from 3.3% to 5.9% (mean 4.3%) (eTable 40, eFigure 44A).

|  | Event rate (%) | | | Contrib. to events (%) | | | Contrib. to ΔEDSS (%) | | |
| --- | --- | --- | --- | --- | --- | --- | --- | --- | --- |
|  | Min | Mean | Max | Min | Mean | Max | Min | Mean | Max |
| Disability accrual (overall) | **19.1** | **20.9** | **21.7** | **-** | **-** | **-** | **-** | **-** | **-** |
| PIRA | 13.9 | 15.3 | 16.7 | 59.8 | 70.1 | 85.8 | 58.9 | 68.9 | 84.7 |
| PIRA with relapse during conf. | 0.0 | 1.3 | 2.8 | 0.0 | 5.0 | 12.1 | 0.0 | 4.8 | 11.6 |
| RAW | 3.3 | 4.3 | 5.9 | 12.1 | 17.0 | 25.4 | 13.0 | 18.0 | 26.9 |
| Undefined | 0.0 | 2.2 | 4.0 | 0.0 | 7.9 | 14.9 | 0.0 | 8.3 | 15.3 |

**eTable 40**: Event rate, event type contribution to the total number of events and event type contribution to the total ΔEDSS, overall and by event type for 144 definitions of disability accrual. **Abbreviations**: Contrib.: Contribution.


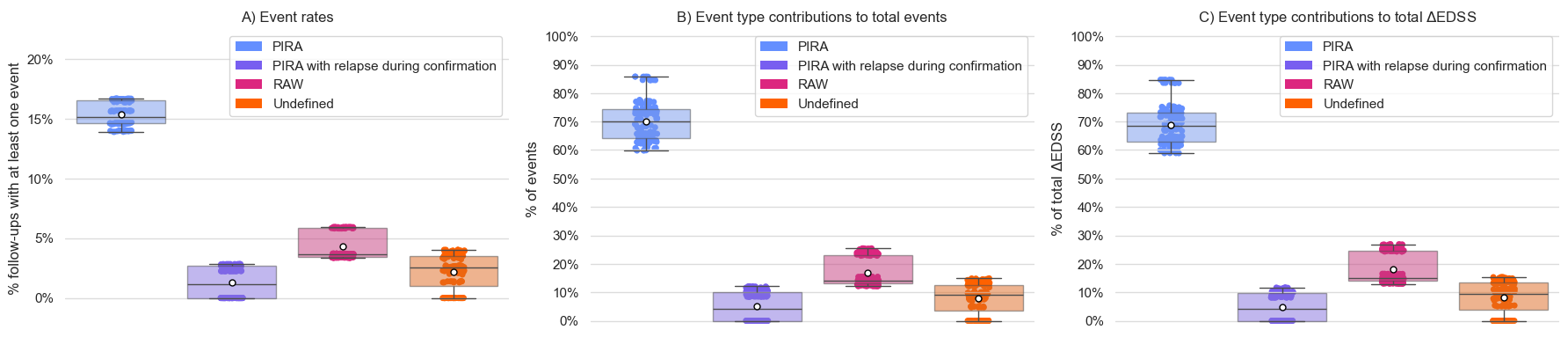


**eFigure 44**: Event rates and contributions by event type for 144 definitions of disability accrual. **A)** Event rates (fraction of follow-ups with at least one event of a given type). **B)** Event type contribution (number of events of a given type over total number of events). **C)** Event type contribution to the total ΔEDSS.

**Overall**, between 871 and 1,016 (median 989) events of any type and total EDSS score increases from 1,079.0 to 1,269.5 (median 1,238.5) points were observed. Of all events, between 59.8% and 85.8% (mean 70.1%) were **PIRA**, and 12.1% to 25.4% (mean 17.0%) were **RAW** (eFigure 44B, eTable 40). Of the total EDSS score increase, 58.9% to 84.7% (mean 68.9%) was due to **PIRA**, and 13.0% to 26.9% (mean 18.0%) was due to **RAW** (eFigure 44C, eTable 40).

### Effect of the options for confirmation

Ignoring confirmation assessments ≤ 30 days after a relapse significantly increased PIRA event rates (from a mean event rate of 14.4% to a mean event rate of 16.3%, p < 0.001) and PIRA event contributions (from a mean event type contribution of 65.2% to a mean event type contribution of 75.1%, p < 0.001) when compared to our implementation of PIRA confirmation. Average event rates and event type contributions for PIRA with relapse during confirmation drop from 2.6% and 10.1%, respectively, to 0.0% by design when using the PIRA confirmation strategy of ^2^, while changes to RAW and undefined event rates and event type contributions are small and not statistically significant (eTable 41, eFigure 45, eFigure 46).

|  | Mean event rate (%) | | p-value | Mean event type contribution (%) | | p-value |
| --- | --- | --- | --- | --- | --- | --- |
|  | Standardized | Our implementation |  | Standardized | Our implementation |  |
| Overall | **20.9** | **20.9** | **0.959** | **-** | **-** | **-** |
| PIRA | 16.3 | 14.4 | <0.001 | 75.1 | 65.2 | <0.001 |
| PIRA rel. conf. | 0.0 | 2.6 | <0.001 | 0.0 | 10.1 | <0.001 |
| RAW | 4.3 | 4.3 | 0.901 | 16.9 | 17.0 | 0.951 |
| Undefined | 2.2 | 2.1 | 0.796 | 8.0 | 7.8 | 0.801 |

**eTable 41**: PIRA confirmation, standardized PIRA version vs. our implementation, 72 comparisons each. A “-” indicates no difference.


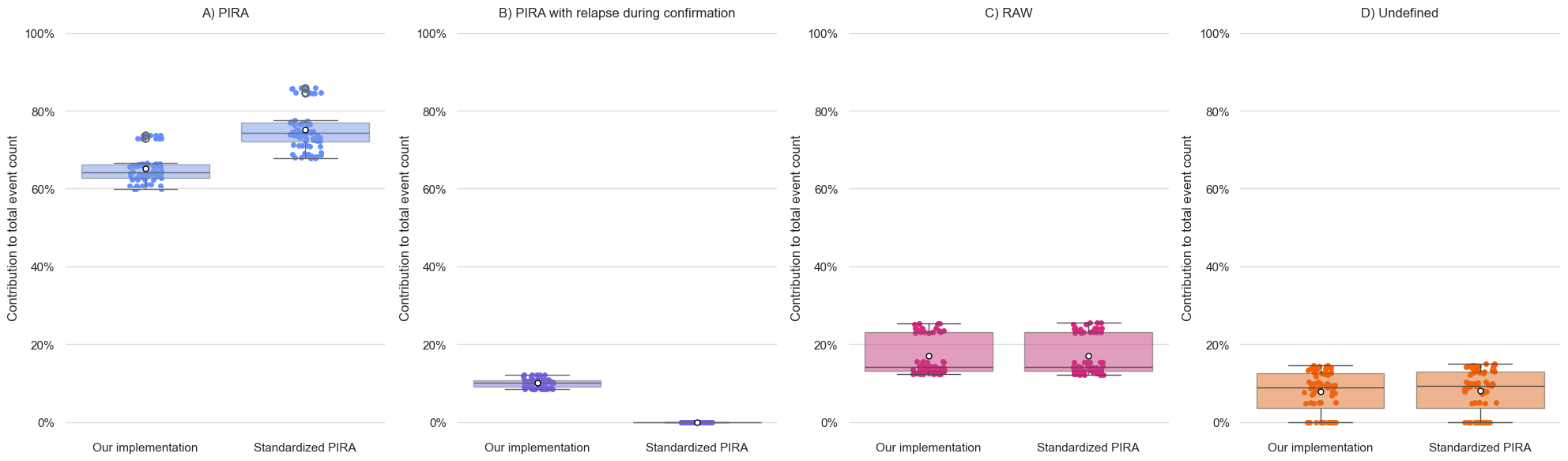


**eFigure 45**: Event type contributions by PIRA confirmation implementation, 72 definitions each. Standardized PIRA ignores assessments ≤ 30 days after a relapse for PIRA confirmation. **A)** PIRA. **B)** PIRA with relapse during confirmation. **C)** RAW. **D)** Undefined.


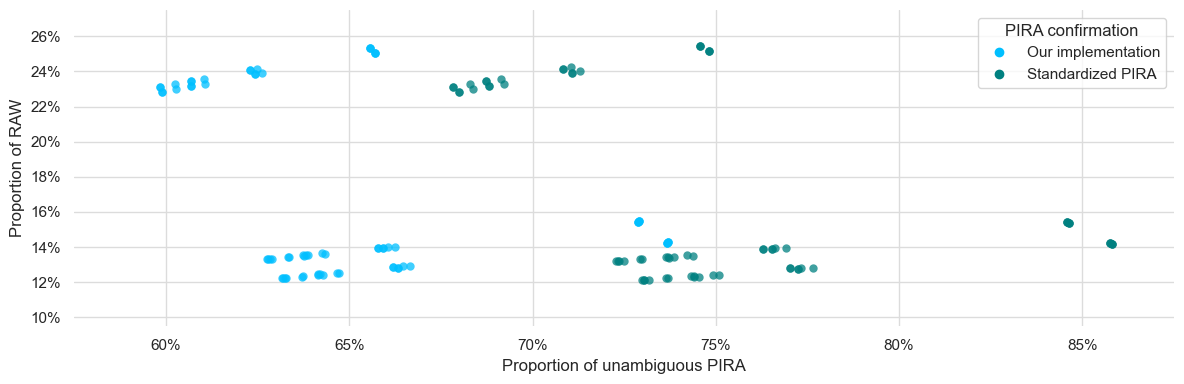


**eFigure 46**: Contribution of PIRA (x-axis) and RAW (y-axis) to the total number of events, 144 definitions. The options for PIRA confirmation implementation are color coded.

### Effects of the RAW window size

Choosing a RAW window size of 180 days pre- and post-relapse significantly decreased the average PIRA contribution (from 72.3% to 66.4%, p < 0.001) and increased the average RAW contribution (from 12.9% to 23.9%, p < 0.001) when compared to a window size of 0 days pre- and 90 days post-relapse (eTable 42, eFigure 47, eFigure 48, eFigure 49).

|  | Mean event contribution (%) | | | p-value when compared to 0/90 | |
| --- | --- | --- | --- | --- | --- |
|  | 0/90 | 30/90 | 180/180 | 30/90 | 180/180 |
| PIRA | 72.3 | 71.7 | 66.4 | 0.638 | <0.001 |
| PIRA with relapses during conf. | 5.4 | 5.3 | 4.4 | 0.866 | 0.293 |
| RAW | 12.9 | 14.1 | 23.9 | <0.001 | <0.001 |
| Undefined | 9.3 | 9.0 | 5.3 | 0.797 | <0.001 |

**eTable 42**: Event type contributions by RAW window size, 480 definitions per window size. P-values are shown for the comparison with the 30/30 window.


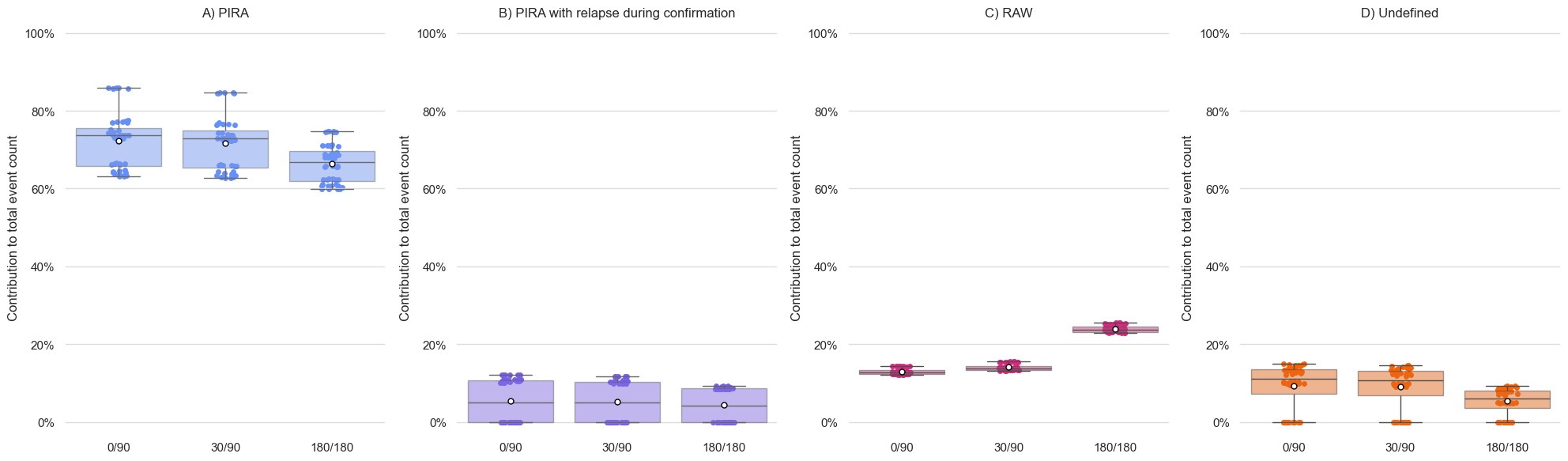


**eFigure 47**: Event type contributions by RAW window size, 48 definitions each. **A)** PIRA. **B)** PIRA with relapse during confirmation. **C)** RAW. **D)** Undefined.


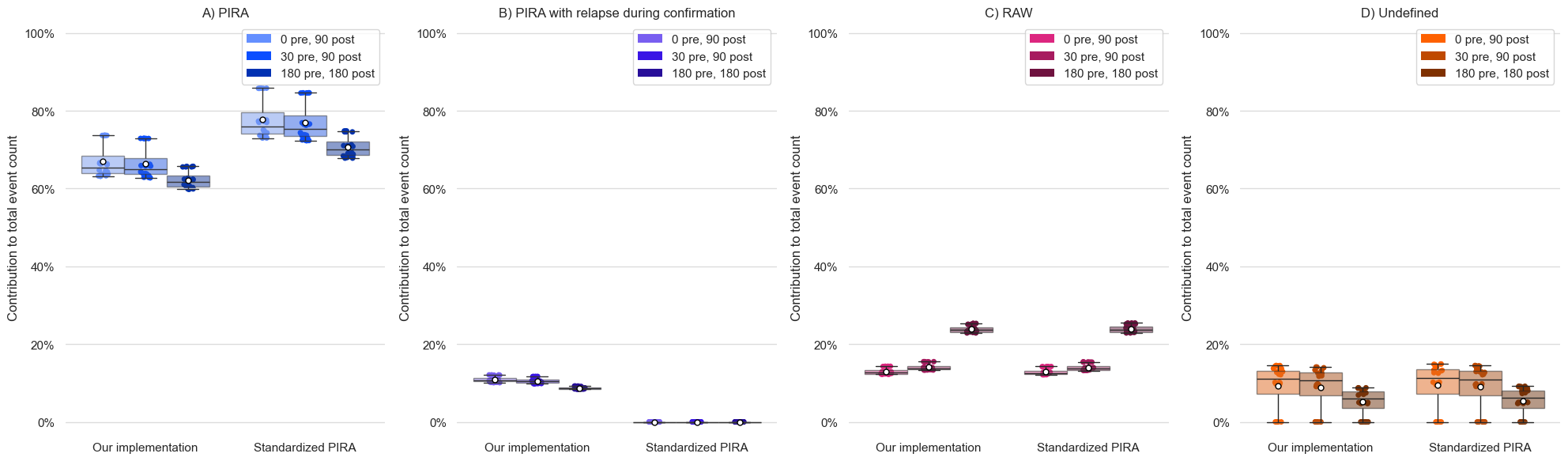


**eFigure 48**: Event type contributions by RAW window size and PIRA confirmation implementation, 24 definitions each. Standardized PIRA ignores assessments ≤ 30 days after a relapse for PIRA confirmation. **A)** PIRA. **B)** PIRA with relapse during confirmation. **C)** RAW. **D)** Undefined.


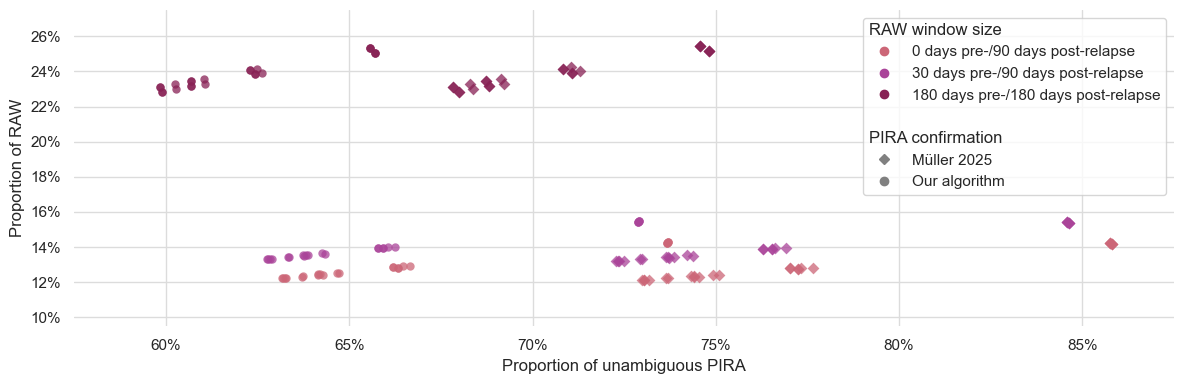


**eFigure 49**: Contribution of PIRA (x-axis) and RAW (y-axis) to the total number of events, 144 definitions. The options for RAW window size are color coded, and the PIRA confirmation implementation is indicated by different markers.

### Effect of the options for undefined worsening

Ignoring events at post-relapse re-baselining assessments significantly decreased the mean **overall** event rate (follow-ups with at least one event irrespective of type) from 21.0% with the “re-baselining only” option to 19.6% with the “never” option (p < 0.001). The “end” and “all” options (that have equal overall event rates by design) increase the mean event rate from 21.0% with the “re-baselining only” option to 21.6% (p = < 0.001). Event rates **by type** do not change significantly, except for undefined worsening, which is ignored with the “never” option (eTable 43).

|  | Mean event rate (%) | | | | p-value vs. RB only | | |
| --- | --- | --- | --- | --- | --- | --- | --- |
|  | RB only | Never | End | All | Never | End | All |
| Disability accrual (overall) | **21.0** | **19.6** | **21.6** | **21.6** | **<0.001** | **<0.001** | **<0.001** |
| PIRA | 15.4 | 15.4 | 15.4 | 15.3 | - | - | 0.673 |
| PIRA with relapse during conf. | 1.3 | 1.3 | 1.3 | 1.3 | - | - | 0.920 |
| RAW | 4.3 | 4.3 | 4.3 | 4.3 | - | - | 0.860 |
| Undefined | 2.2 | 0.0 | 3.0 | 3.4 | <0.001 | <0.001 | <0.001 |

**eTable 43**: Mean event rates for the four options for undefined worsening, and p-values (permutation test) for the comparison of the “never”, “all”, and “end” options to the default “re-baselining only” option (36 comparisons each). **Abbreviations**: RB only: re-baselining only. A “-” indicates no difference.

Ignoring events at post-relapse re-baselining assessments also significantly increased PIRA and RAW contributions to the total event count compared to the default re-baselining only mode, and using the “all” option significantly reduces PIRA and RAW contributions while increasing the contribution of undefined worsening (eFigure 50, eTable 44). The average contribution of **PIRA** to the total event count increased from 69.9% to 76.2% (p < 0.001) when ignoring all undefined events instead of allowing undefined events at re-baselining assessments (all other definition aspects equal, i.e. 36 comparisons) (eTable 44, eFigure 51A).


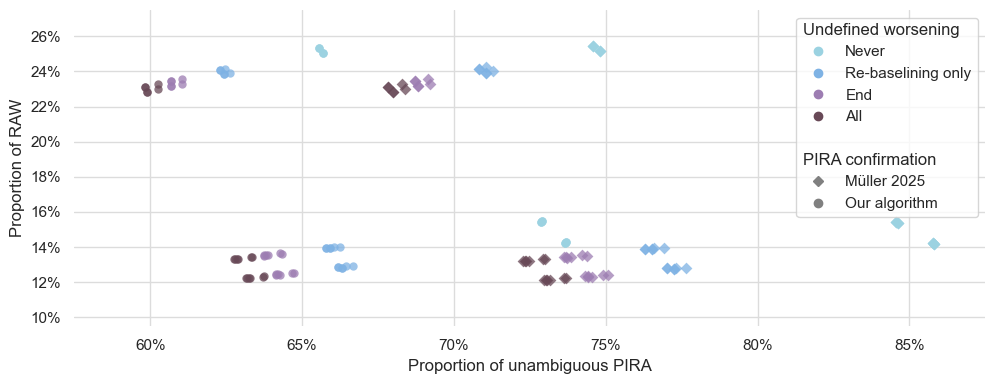


**eFigure 50**: Contribution of PIRA (x-axis) and RAW (y-axis) to the total number of events, 144 definitions. The options for undefined worsening are color coded, and the PIRA confirmation implementation is indicated by different markers.

|  | Mean event contribution (%) | | | | p-value vs. RB only | | |
| --- | --- | --- | --- | --- | --- | --- | --- |
|  | RB only | Never | End | All | Never | End | All |
| PIRA | 69.9 | 76.2 | 67.8 | 66.7 | <0.001 | 0.088 | 0.014 |
| PIRA with relapse during conf. | 5.0 | 5.5 | 4.9 | 4.7 | 0.701 | 0.894 | 0.835 |
| RAW | 16.9 | 18.3 | 16.4 | 16.2 | 0.252 | 0.692 | 0.484 |
| Undefined | 8.1 | 0.0 | 10.9 | 12.4 | <0.001 | <0.001 | <0.001 |

**eTable 44**: Event type contributions by undefined worsening annotation mode, 36 definitions per mode. Abbreviations: RB only: re-baselining only.


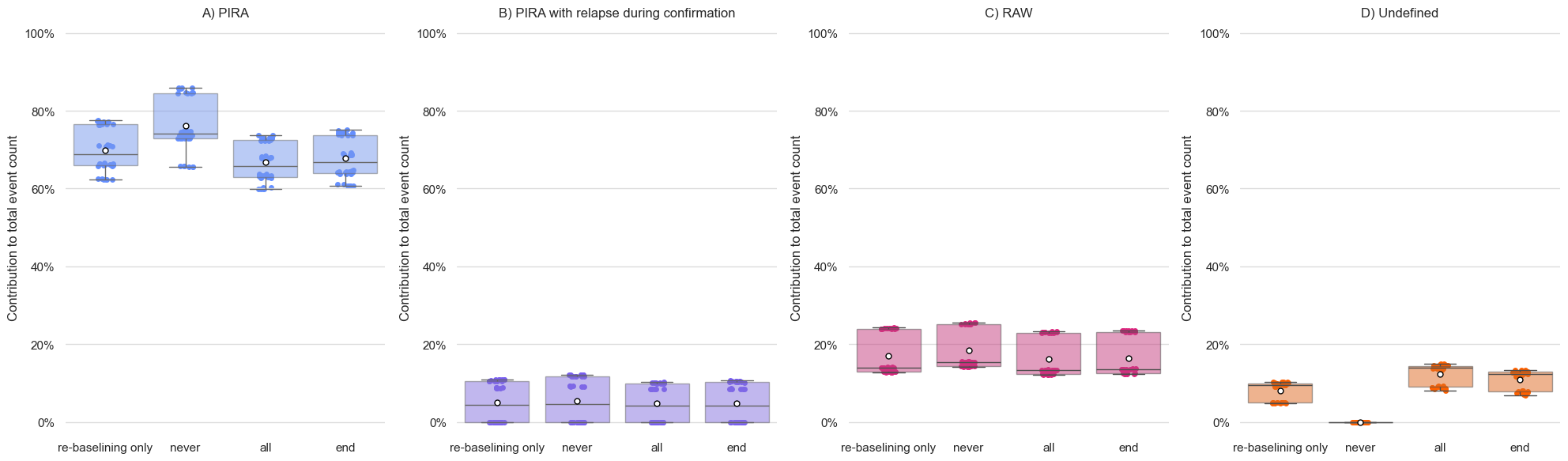


**eFigure 51**: Contributions of each event type to the overall event count by option for undefined worsening, 360 definitions each. **A)** PIRA contribution. **B)** PIRA with relapse during confirmation contribution. **C)** RAW contribution. **D)** Undefined contribution.

Requiring a potential undefined event score to have a greater or equal or a greater score than the current RAW/PIRA baseline does not have a large impact on event rates or event type contributions. The mean overall event rate is 21.4% for the “any” and “equal or greater” option, and 21.3% for the “greater only” option (36 definitions each; the “never” option for undefined worsening excluded because only undefined events are affected by this threshold choice). The proportion of follow-ups with at least one undefined event decreased from 2.9% with the “any” option to 2.8% with the “greater only” option (p = 0.420, 36 comparisons, “never” for undefined excluded), while the rates for the other event types remain unchanged (eTable 45). Changes to the event type contributions are small and not statistically significant (eTable 46).

|  | Mean event rate (%) | | | p-value vs. “any” | |
| --- | --- | --- | --- | --- | --- |
|  | Any | Equal or greater | Greater only | Equal or greater | Greater only |
| PIRA | 15.3 | 15.3 | 15.3 | - | - |
| PIRA with relapse during conf. | 1.3 | 1.3 | 1.3 | - | - |
| RAW | 4.3 | 4.3 | 4.3 | - | - |
| Undefined | 2.9 | 2.9 | 2.8 | - | 0.420 |

**eTable 45**: Event rates by undefined worsening threshold mode, 36 definitions per mode. A “-” indicates no difference.

|  | Mean event type contributions (%) | | | p-value vs. “any” | |
| --- | --- | --- | --- | --- | --- |
|  | Any | Equal or greater | Greater only | Equal or greater | Greater only |
| PIRA | 68.0 | 68.0 | 68.4 | 0.984 | 0.732 |
| PIRA with relapse during conf. | 4.9 | 4.9 | 4.9 | 0.978 | 0.952 |
| RAW | 16.5 | 16.5 | 16.6 | 0.994 | 0.907 |
| Undefined | 10.7 | 10.6 | 10.1 | 0.955 | 0.431 |

**eTable 46**: Event type contributions by threshold mode, 36 definitions per mode.

### Effect of baseline choice

Changing the roving baseline confirmation implementation from our algorithm’s version (candidate score and confirmation scores lower than previous reference, new reference is the maximum of candidate and confirmation scores) to the standardized PIRA definition’s version (candidate score lower than previous reference, confirmation scores equal or lower than candidate, new reference is the minimum of confirmation scores) does not change event rates or event type contributions significantly (eTable 47, eFigure 52, eFigure 53).


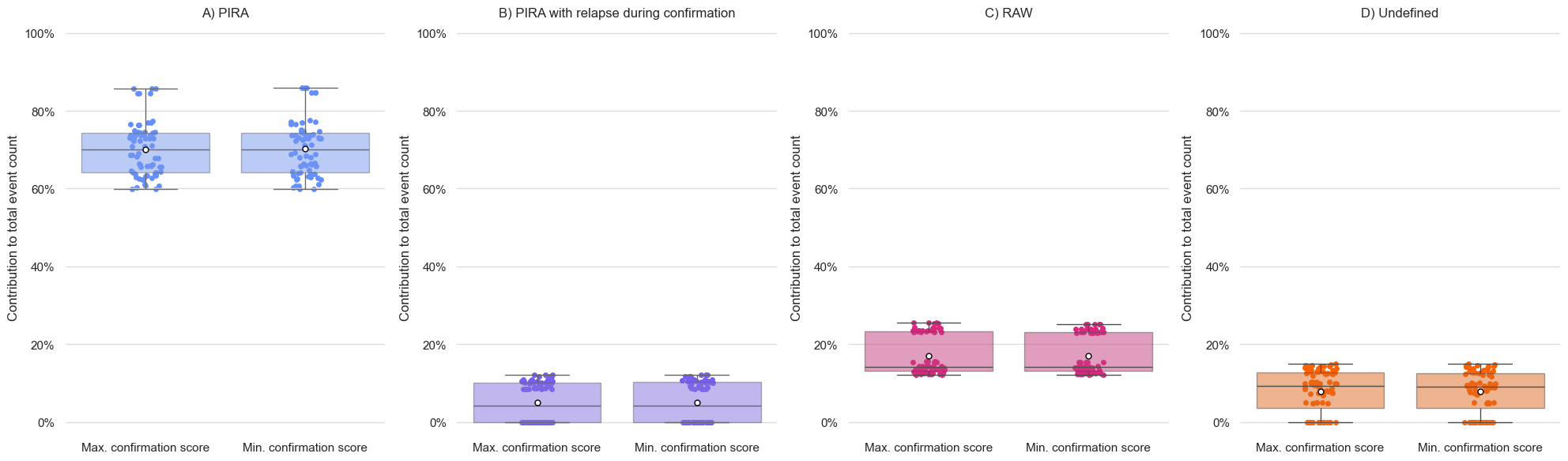


**eFigure 52**: Event type contributions by roving reference confirmation implementation, 72 definitions each. **A)** PIRA. **B)** PIRA with relapse during confirmation. **C)** RAW. **D)** Undefined.

|  | Mean event rate (%) | | p-value | Mean event type contribution (%) | | p-value |
| --- | --- | --- | --- | --- | --- | --- |
|  | Standardized | Our implementation |  | Standardized | Our implementation |  |
| Overall | **20.9** | **20.9** | **0.992** | **-** | **-** | **-** |
| PIRA | 15.4 | 15.3 | 0.846 | 70.2 | 70.1 | 0.921 |
| PIRA rel. conf. | 1.3 | 1.3 | 0.961 | 5.0 | 5.0 | 0.972 |
| RAW | 4.3 | 4.3 | 0.825 | 16.9 | 17.0 | 0.912 |
| Undefined | 2.2 | 2.2 | 0.967 | 7.8 | 7.9 | 0.946 |

**eTable 47**: Roving reference confirmation, standardized PIRA version vs. our implementation, 72 comparisons each. A “-” indicates no difference.


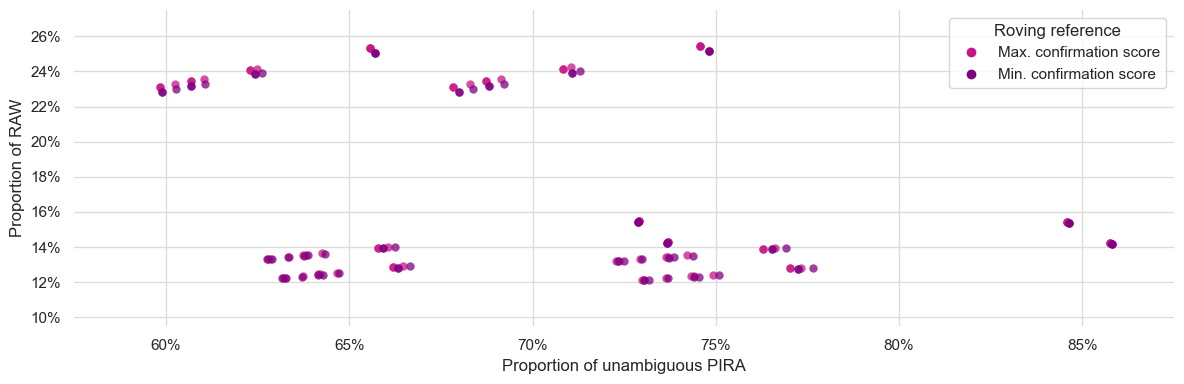


**eFigure 53**: Contribution of PIRA (x-axis) and RAW (y-axis) to the total number of events, 144 definitions. The options for roving reference confirmation are color coded.

### Example of a follow-up with a large variation in event type attribution


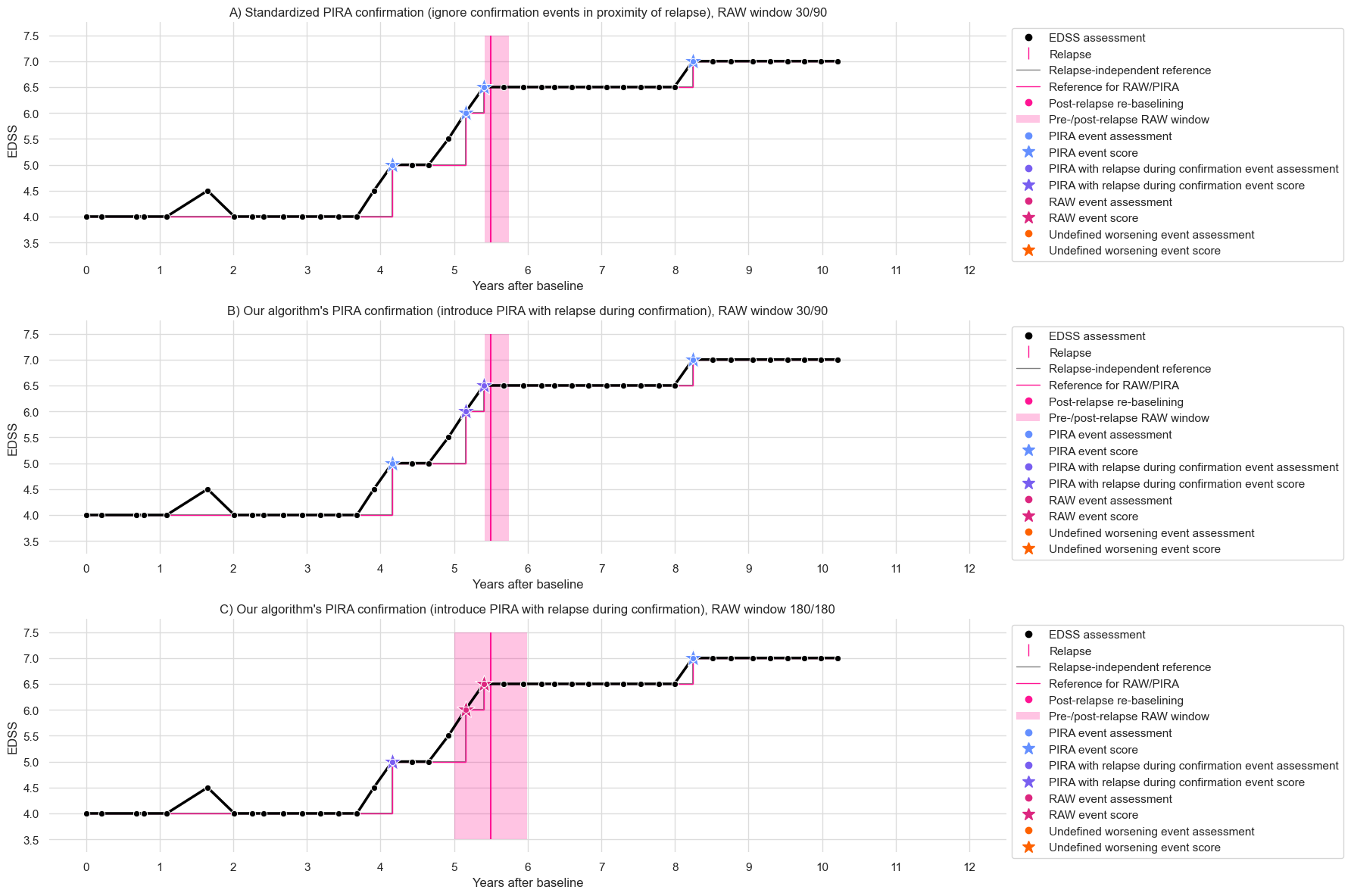


**eFigure 54**: RMS follow-up from AMSTR under three definitions compatible with the standardized definition of PIRA. Undefined progression at re-baselining only, 90 days all-confirmed roving reference (our confirmation approach). **A)** Standardized PIRA confirmation implementation (ignore confirmation events in proximity of relapse), RAW window 30 days pre- and 90 days post-relapse. 4 PIRA events. **B)** Our PIRA confirmation implementation (introduce PIRA with relapse during confirmation), RAW window 30 days pre- and 90 days post-relapse. 2 PIRA events, 2 PIRA with relapse during confirmation events. **C)** Our PIRA confirmation implementation (introduce PIRA with relapse during confirmation), RAW window 180 days pre- and 180 days post-relapse. 1 PIRA event, 1 PIRA with relapse during confirmation event, 2 RAW events.

# Supplemental methods

## General remarks

This document is illustrated with examples tailored to showcase the properties of individual definition options and combinations thereof. They are not necessarily based on actual real-world examples, and they use arbitrary time units and time steps optimized for readability and easy calculation. The algorithm does not enforce units or rely on pre-defined options (e.g. RAW windows of only 30 days pre- and post-relapse). See methods.ipynb on the project’s GitHub page (https://github.com/drstrupf/multiple-sclerosis-disability-progression) for how the visualizations were created.

## Notation

*S = (s_0_, s_1_, s_2_, ..., s_n_)*: a series of EDSS scores from one baseline and *n* follow-up assessments

*s_i_*: the EDSS score of the *i*-th assessment

*t_i_*: the time of the *i*-th assessment

*r_0_*: the fixed baseline EDSS, with *r_0_ = s_0_*

*r_i_*: the reference EDSS score for the *i*-th assessment

# Relapse-independent definition aspects

This section covers all aspects of defining EDSS worsening that are not related to relapses, i.e. general minimal increase requirements, confirmation conditions, minimal distance conditions, baselines, post-event re-baselining, and event merging. These definitional aspects allow the identification of disability accrual events in the absence of relapses, e.g. in PPMS cohorts. In the absence of relapses, all events are classified as Progression Independent of Relapse Activity (PIRA).

## Minimal required increase

The **minimal increase condition** requires a score to be equal or greater than the reference plus a minimum increase:

*s_i_ ≥ r_i_ + I(r_i_),*

where the minimum required increase *I(r_i_)* may depend on the current reference. A common definition of *I(r)* is

+ 1.5 if *r* = 0

*I(r)* = + 1.0 if 1.0 ≤ *r* < 5.5

+ 0.5 if *r* ≥ 5.5.

The algorithm allows the choice of a cutoff at which the requirement changes from + 1.0 to + 0.5 points, and an optional requirement of + 1.5 if the reference score is 0.


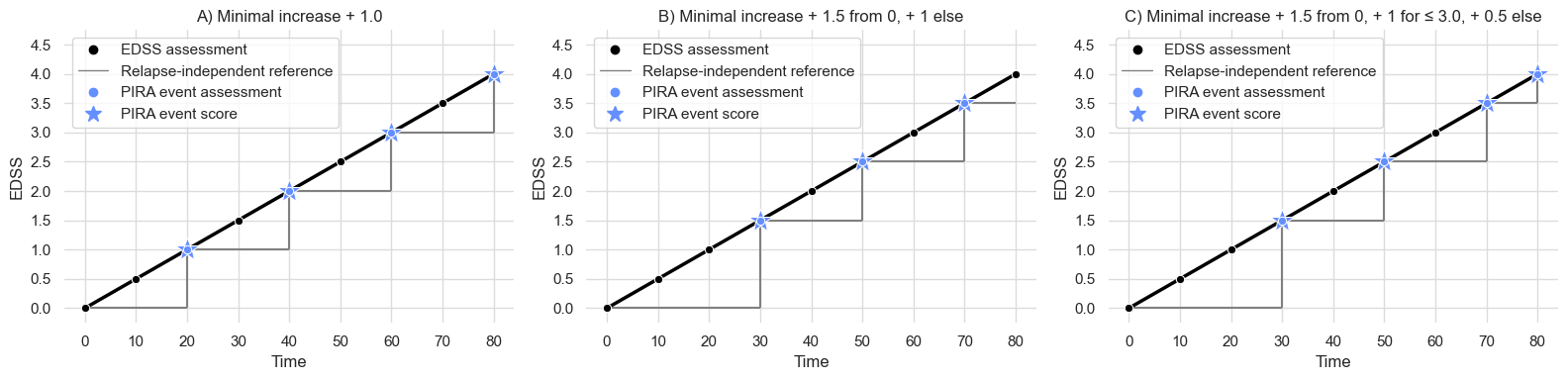


**eFigure 55**: Minimal increase options. **A)** Minimal increase + 1.0 irrespective of the reference. **B)** Minimal increase + 1.5 for reference 0, + 1.0 else. **C)** Minimal increase + 1.5 for reference 0, minimal increase + 1.0 for references up to and including 3.0, and + 0.5 else. The reference is reset after each event.

## Event confirmation

### Confirmed score

If an event requires confirmation, all confirmation scores must satisfy the confirmation condition (see next subsection) and the event score is the minimum of all confirmation scores (eFigure 56).


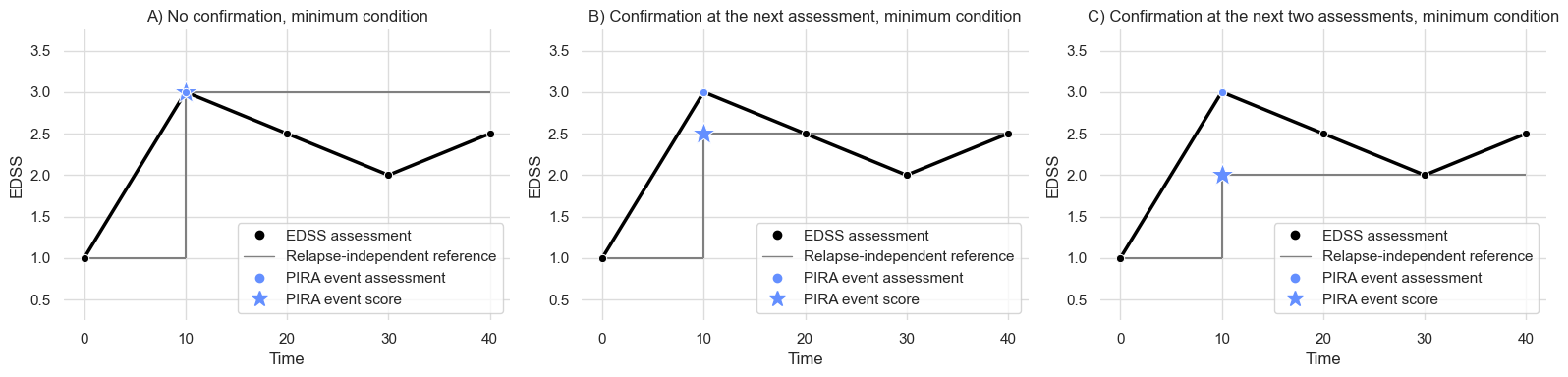


**eFigure 56:** Confirmed score. **A)** No confirmation required. The event score for the increase at step 10 is 3.0. **B)** Confirmation at the next assessment required. The score at the next assessment also fulfills the criterion of an increase of at least + 1.0 with respect to the baseline, so the increase at step 10 is confirmed, but only at 2.5. **C)** Confirmation at the next two assessments required. The scores at the next two assessment also fulfill the criterion of an increase of at least + 1.0 with respect to the baseline, so the increase at step 10 is confirmed, but only at 2.0. Minimal increase + 1.0.

### Confirmation condition

For an increase *s_i_* to be **confirmed**, the follow-up assessment scores have to satisfy a confirmation condition. For the **confirmation condition**, there are two options: values have to be either a) equal or greater than *r_i_ + I(r_i_)* (“minimum”), or b) equal or greater than *s_i_* (“monotonic”). Consider e.g. a reference EDSS *r* = 1.0, and a potential disability accrual event with *s* = 2.5. Following variant a), all confirmation scores have to be ≥ 1.0 + 1.0 (eFigure 57A), whereas following variant b) all confirmation scores have to be ≥ 2.5 (eFigure 57B).


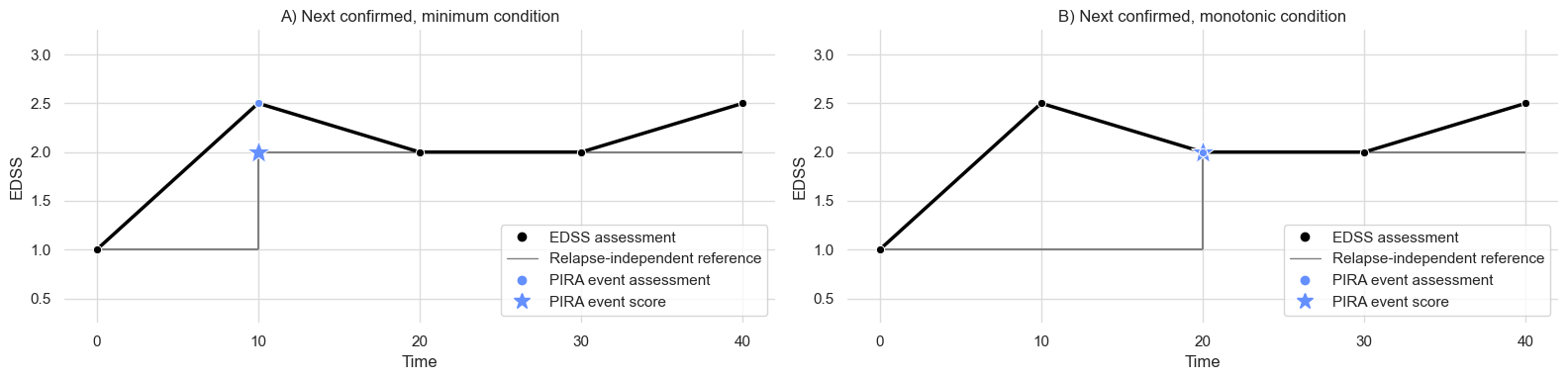


**eFigure 57:** Confirmation conditions. **A)** Minimum condition. The score at step 20 fulfills the confirmation criterion, so the increase at step 10 is a confirmed event with event score 2.0. **B)** Monotonic condition. The increase at step 10 is not a confirmed event because the confirmation score at step 20 is lower. However, the increase at step 20 is confirmed by the score at step 30. Minimal increase + 1.0, confirmation at the next assessment required.

### Confirmation interval

The **confirmation scores** for a potential event at time *t_i_* and a given **confirmation interval** *Δt* are either a) all scores *s_j_* at times *t_j_* with *t_i_* < *t_j_* ≤ *t_i_* + *Δt* ∪ min{*t_j_* | *t_j_* ≥ *t_i_* + Δt}, i.e. all scores within the confirmation time plus the first score *t_j_* that satisfies *t_j_* ≥ *t_i_* + Δt (“all”, eFigure 58A), or b) only the score *s_j_* at time *t_j_* with *t_j_* = min{*t_j_* | *t_j_* ≥ *t_i_* + Δt}, i.e. the first score at or after the required confirmation time (“last”, eFigure 58B). The confirmation interval can be freely chosen.


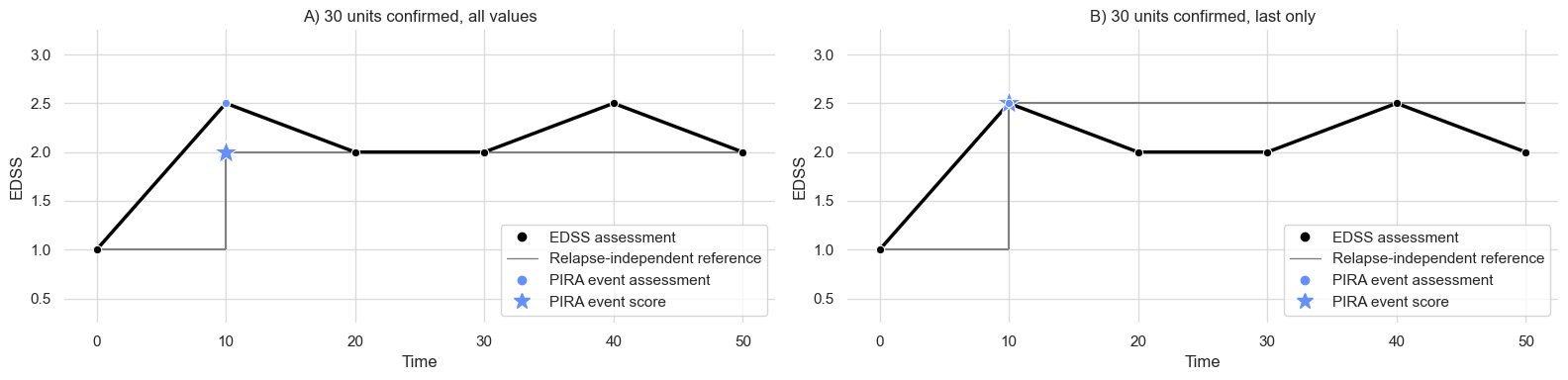


**eFigure 58:** Confirmation value inclusion options. **A)** All scores within the confirmation interval must satisfy the confirmation condition. For a confirmation over 30 units, the confirmation scores for the event at step 10 are thus the scores at steps 20, 30, and 40, and the confirmed event score is 2.0. **B)** Only the first score at or after the required confirmation time must satisfy the confirmation condition. The confirmation score for the event at step 10 is thus the score at step 40, and the confirmed event score is 2.5. Minimal increase + 1.0, minimum confirmation condition.

### Confirmation distance types

Confirmation can be over a certain minimal distance or sustained over the entire follow-up. Choosing a confirmation interval smaller than the smallest time step in the data amounts to requiring confirmation at the next assessment.


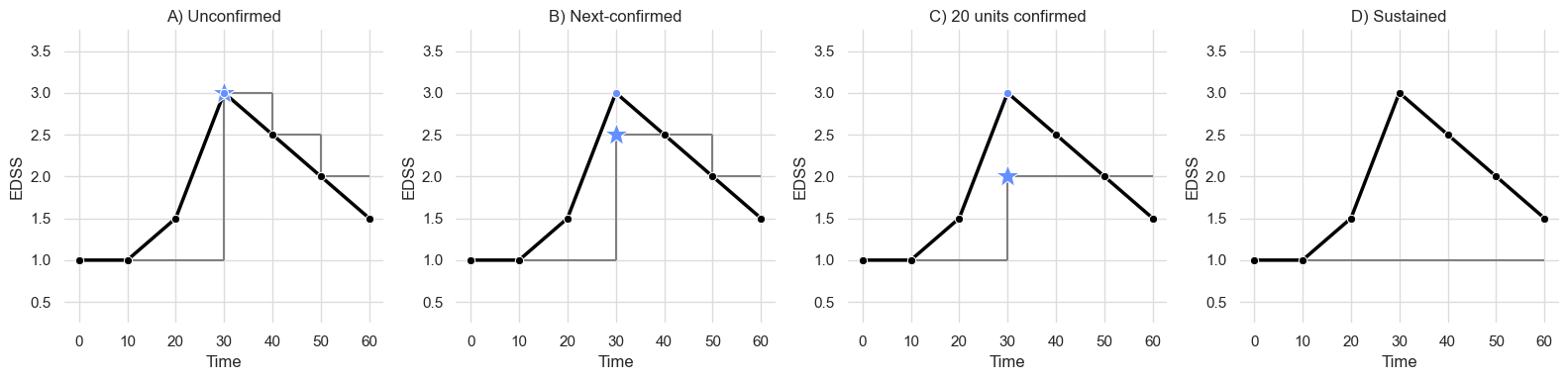


**eFigure 59:** Confirmation distance types. **A)** Unconfirmed disability accrual, event at step 30 with event score 3.0. **B)** Next-confirmed disability accrual, event at step 30 with event score 2.5. **C)** 20 units confirmed, event at step 30 with event score 2.0. **D)** Sustained, i.e. there is no event because the last score of the follow-up is not sufficiently larger than the reference. Minimum increase + 1.0, minimum condition. **Legend:** Black dots represent EDSS scores, blue stars represent PIRA event scores, blue dots represent PIRA event assessments, and the grey curve represents the reference.

### Confirmation distance tolerances and constraints

The algorithm supports **left-hand tolerance** for the confirmation distance, i.e. confirmation scores are all scores *s_j_* at times *t_j_* with *t_i_* < *t_j_* ≤ *t_i_* + *Δt* – tolerance ∪ min{*t_j_* | *t_j_* ≥ *t_i_* + Δt - tolerance} (eFigure 60).


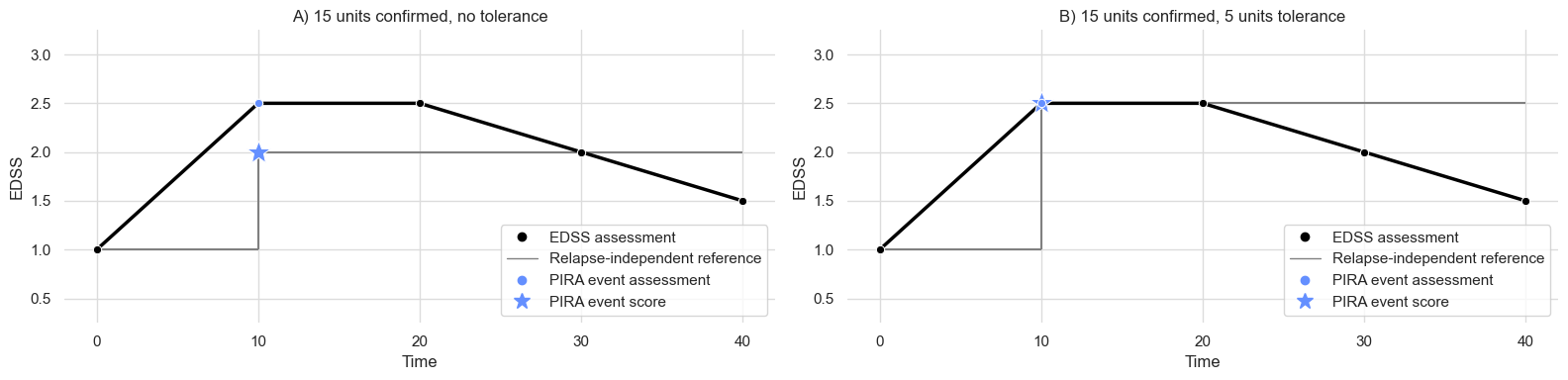


**eFigure 60:** Left-hand tolerance for event confirmation, 15 units confirmation required. **A)** No tolerance, i.e. the confirmation scores for the event at step 10 are the scores at steps 20 and 30 (the first one that satisfies *t_j_* ≥ *t_i_* + Δt), thus the confirmed event score is 2.0. **B)** 5 units tolerance, thus the score at step 20 already satisfies the *t_j_* ≥ *t_i_* + Δt - tolerance condition, and the score at step 30 is not required for confirmation anymore. Minimum increase + 1.0, minimum condition.

The algorithm supports **right-hand constraints** for the confirmation distance, i.e. the time *t_min_* of the first score that satisfies the confirmation distance (*t_min_* = min{*t_j_* | *t_j_* ≥ *t_i_* + Δt }) must satisfy *t_min_* ≤ *t_i_* + Δt + constraint (eFigure 61).


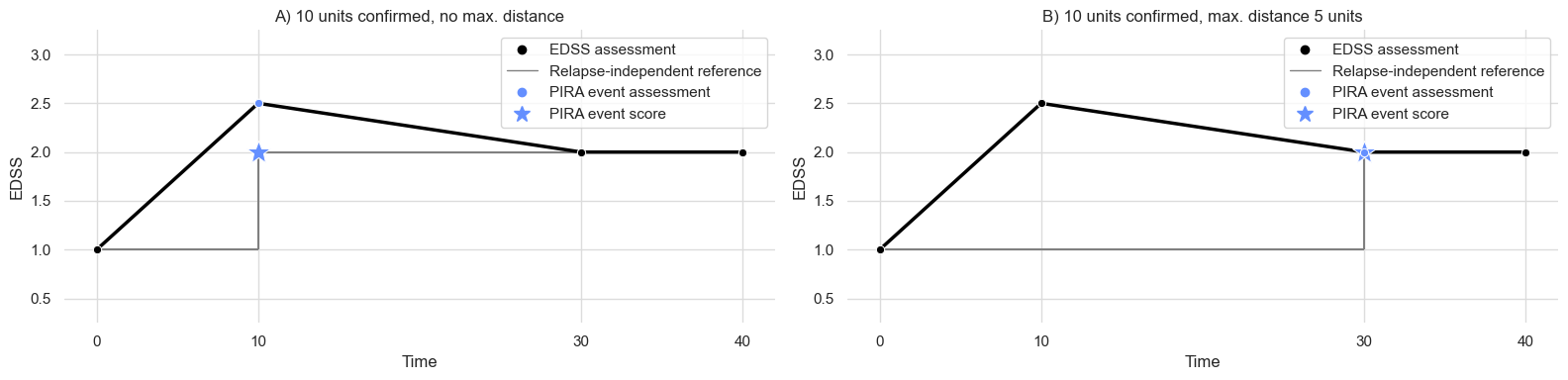


**eFigure 61:** Confirmation over 10 units required. **A)** No right-hand constraint, i.e. the event at step 10 is confirmed by the score at step 30. **B)** Maximal distance from the minimal confirmation distance of 5 units. The score at step 30, which is the first to satisfy the 10 units minimal distance, is more than 10 + 5 units from the event, thus it does not qualify as confirmation score. However, the score at step 30 is a confirmed disability accrual with respect to the baseline 1.0 since the score at step 40 satisfies the minimal and maximal confirmation distance requirements.

Allowing a left-hand tolerance is equivalent to reducing the confirmation distance by the provided tolerance *only* if no right-hand side constraint is implemented (eFigure 62).


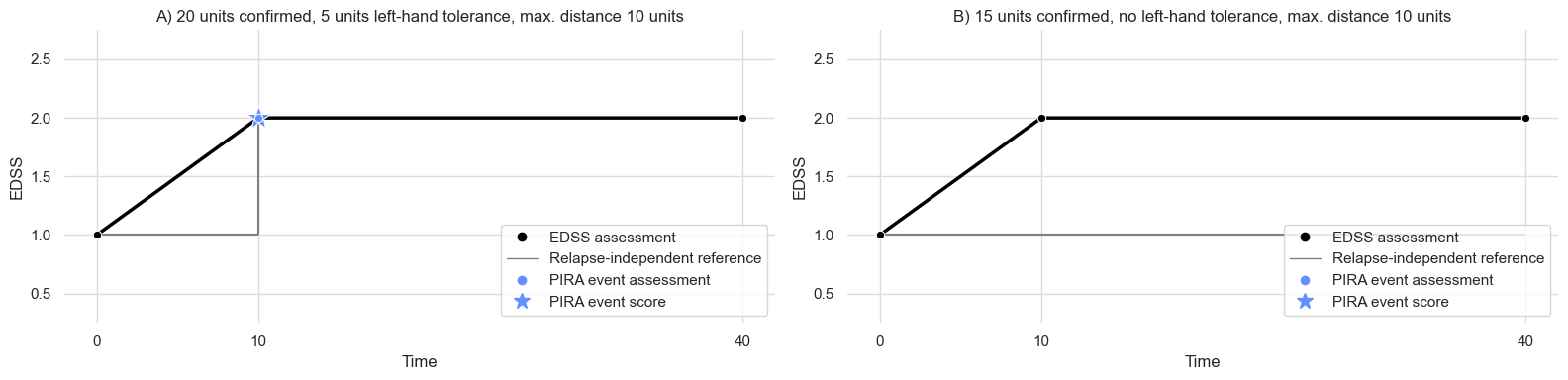


**eFigure 62:** Combination of left-hand tolerance and right-hand constraint. **A)** 20 units confirmation with 5 units tolerance. The maximal timestamp (right-hand constraint) for a confirmation assessment for an event at step 10 is *t_i_* + Δt + constraint = 10 + 20 + 10 = 40, i.e. the assessment at step 40 is a valid confirmation assessment. **B)** Reducing the minimal distance instead of allowing a left-hand tolerance yields a maximal timestamp *t_i_* + Δt + constraint = 10 + 15 + 10 = 35, i.e. the assessment at step 40 is not a valid confirmation assessment.

### Special options for sustained

For sustained disability accrual, all scores after an event must satisfy the confirmation condition, and there must be at least one assessment after the event candidate. The “last” option for the inclusion of confirmation values is not available for sustained disability accrual. The algorithm supports requiring a minimum duration of the post-event follow-up. If a minimal post-event follow-up distance is required, there must be at least one assessment with a timestamp *t_j_* that satisfies *t_j_* ≥ *t_i_* + minimum duration (eFigure 63). Sustained disability accrual ignores left-hand tolerance and right-hand constraints.


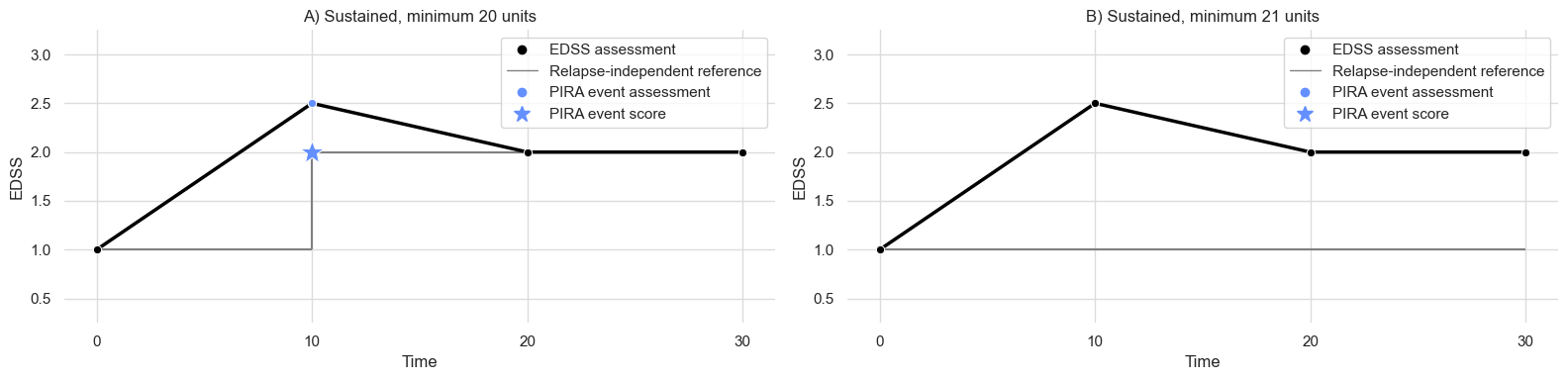


**eFigure 63:** Minimal duration requirement for sustained disability accrual. **A)** Minimum duration 20 units. This condition is satisfied for the increase at step 10 as the assessment at step 30 satisfies *t_j_* ≥ *t_i_* + minimum duration = 10 + 20 = 30. **B)** Minimum duration 21 units. The increase at step 10 is not confirmed since there is no assessment with a timestamp *t_j_* that satisfies *t_j_* ≥ *t_i_* + minimum duration = 10 + 21 = 31. Minimum increase + 1.0, minimum condition.

### Optional exemption from confirmation for the last assessment

The algorithm allows exempting the last assessment from the confirmation requirement (eFigure 64).


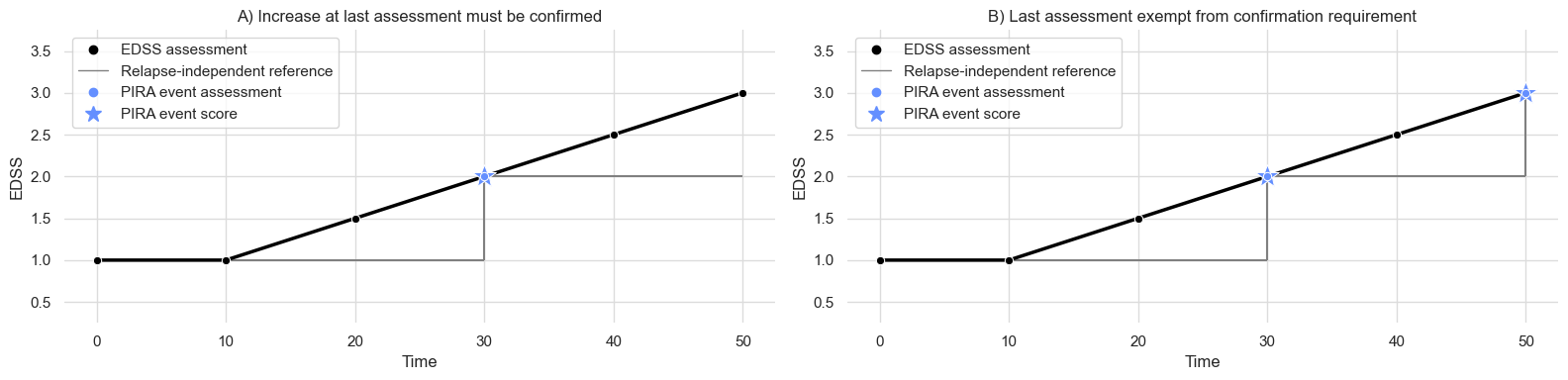


**eFigure 64: A)** All increases must be confirmed, thus the increase at step 50 does not qualify as disability accrual. **B)** The last assessment is exempt from the confirmation requirement, thus the increase at step 50 qualifies as disability accrual. Minimum increase + 1.0, next-confirmed.

## Minimal distance

The algorithm supports requiring optional minimal distance conditions for disability accrual event candidates. The **minimal distance condition** requires a minimum duration between the score and either a) the previous assessment (eFigure 65) or b) the reference assessment (eFigure 66):

1. *t_i_ ≥ t_i - 1_ + Δt*
2. *t_i_ ≥ t_r_ + Δt*

where *t_r_* is the timestamp of the assessment that now serves as the reference. For disability accrual over fixed baseline, b) is equivalent to *t_i_ ≥ t_0_ + Δt*.

### Minimal distance to previous assessment

When requiring a minimal distance to the previous assessment, a score increase at *t_i_* can only qualify as an event if it satisfies *t_i_ ≥ t_i - 1_ + Δt*, where *t_i - 1_* is the timestamp of the previous assessment (eFigure 65).


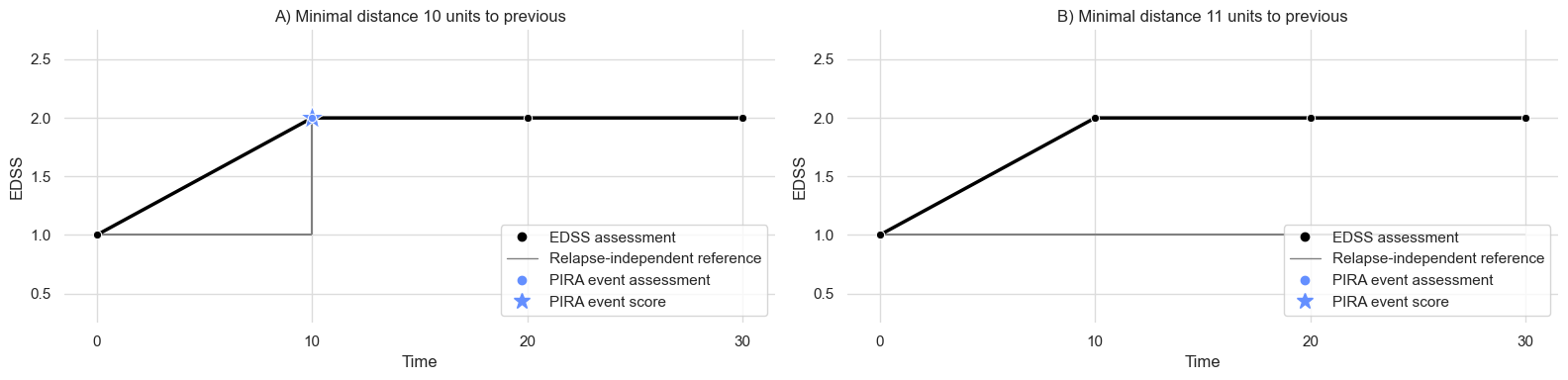


**eFigure 65**: Minimal distance to the previous assessment. **A)** The increase at step 10 is ≥ 10 units from the last assessment (step 0), thus it qualifies as a disability accrual event. **B)** The increase at step 10 is < 11 units from the last assessment (step 0), thus it does not qualify as a disability accrual event.

### Minimal distance to the reference assessment

When requiring a minimal distance to the reference assessment, a score increase at *t_i_* can only qualify as an event if it satisfies *t_i_ ≥ t_r_ + Δt*, where *t_r_* is the timestamp of the reference assessment (eFigure 66).


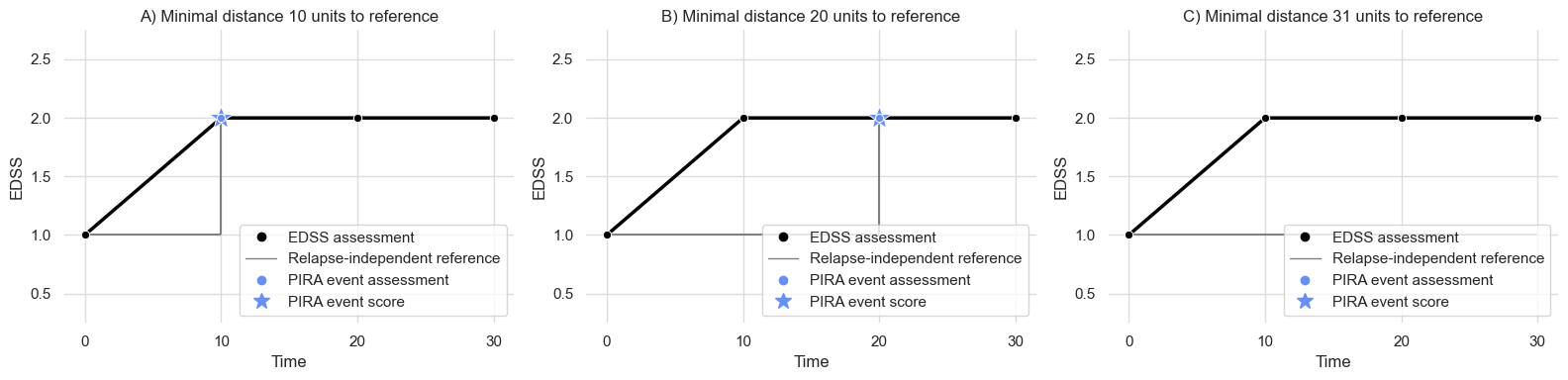


**eFigure 66**: Minimal distance to the reference, no confirmation requirement. **A)** Minimal distance 10 units. The increase at step 10 is ≥ 10 units from the reference (step 0), thus it qualifies as a disability accrual event. **B)** Minimal distance 20 units. The increase at step 10 is < 20 units from the last assessment (step 0), thus it does not qualify as a disability accrual event. However, the score at step 20 satisfies the minimal increment and minimal distance condition, thus it is a disability accrual event. **C)** Minimal distance 31 units. No assessment satisfies the minimal distance to reference condition, thus there are no disability accrual events.

### Minimal distance and confirmation

When requiring a minimal distance in combination with requiring event confirmation, the confirmation interval starts at the assessment that satisfies the minimal distance requirement (the event candidate), not at the first increase (eFigure 67).


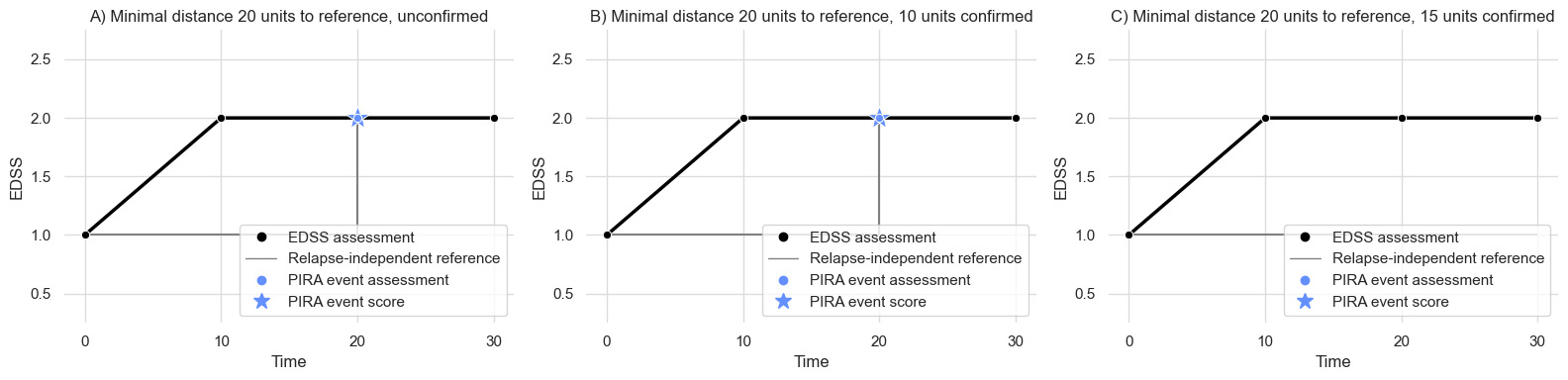


**eFigure 67**: Minimal distance to reference (20 units) and confirmation condition. **A)** The assessment at step 20 is the first increase that satisfies the minimal distance condition. It does not require confirmation, thus it qualifies as disability accrual. **B)** The assessment at step 20 is the first increase that satisfies the minimal distance condition, and the assessment at step 30 satisfies the confirmation requirement of 10 units. **C)** The assessment at step 20 is the first increase that satisfies the minimal distance condition, but it is not confirmed since there is no assessment at time ≥ 20 + 15.

### Minimal distance to roving reference

For disability accrual over roving baseline (see Baselines section), the minimal distance to reference can lead to a counterintuitive loss of sensitivity if an event is preceded by a decrease in EDSS where the last (lowest) score before the potential event is too close (eFigure 68).


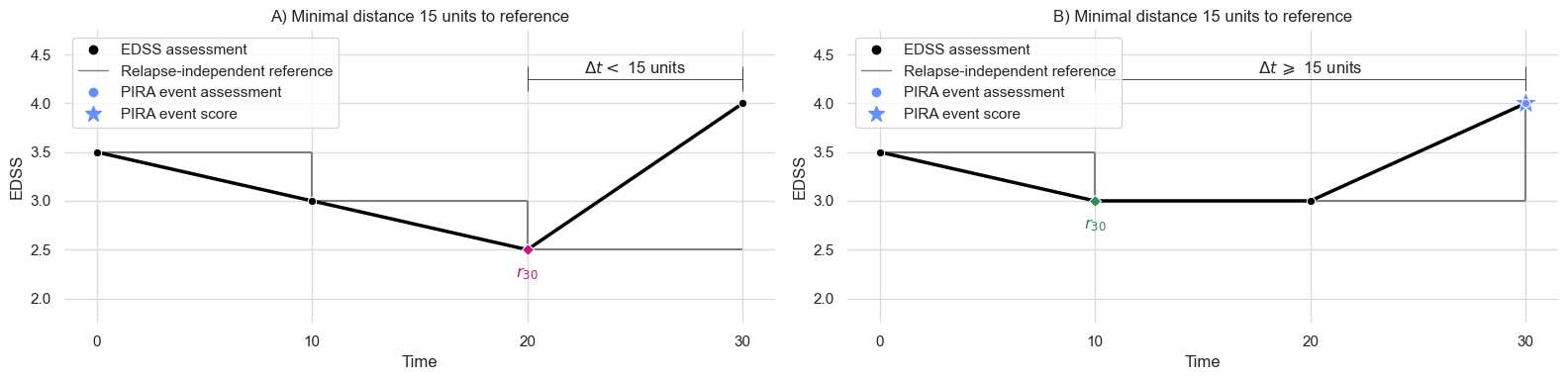


**eFigure 68**: Minimal distance to reference with an unconfirmed roving reference. **A)** The score at step 20 qualifies as a new roving reference for the event at step 30, thus the distance between the increase at step 30 and the reference does not satisfy the minimal distance condition, and the increase at step 30 does not qualify as event. **B)** The roving reference is last reset at step 10, thus the distance between the reference and the event candidate at step 30 satisfies the minimal distance condition and the increase at step 30 qualifies as an event.

To mitigate this problem, the algorithm can optionally instead check whether there is *any* previous reference that is low enough for an increase to count as an event and that satisfies the minimal distance requirement (“backtracking”, eFigure 69).


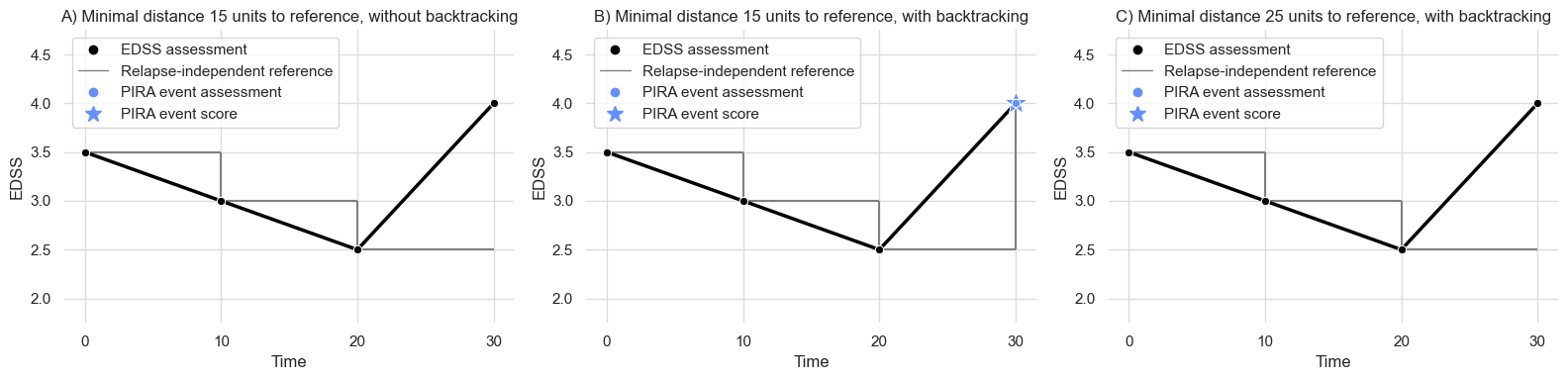


**eFigure 69**: Minimal distance to reference with a roving reference. **A)** The score at step 20 qualifies as a new roving reference, thus the distance between the increase at step 30 and the reference does not satisfy the minimal distance condition. However, the increase at step 30 would be an event if the score at step 20 was 3.0 instead of 2.5, because in this case the reference would be the score at step 10 (see eFigure 68). **B)** With backtracking, if there is a reference assessment with a score low enough for the increase at 30 to be an event and that satisfies the minimal distance condition, the increase at step 30 qualifies as an event. This is the case for the assessment at step 10. **C)** The last reference that would satisfy the minimal distance condition (the assessment at step 0) is not low enough for the score at step 30 to count as an event.

Backtracking uses the closest (and thus lowest) reference that satisfies the minimal distance condition, and the confirmation condition has to be satisfied with respect to this reference (eFigure 70).


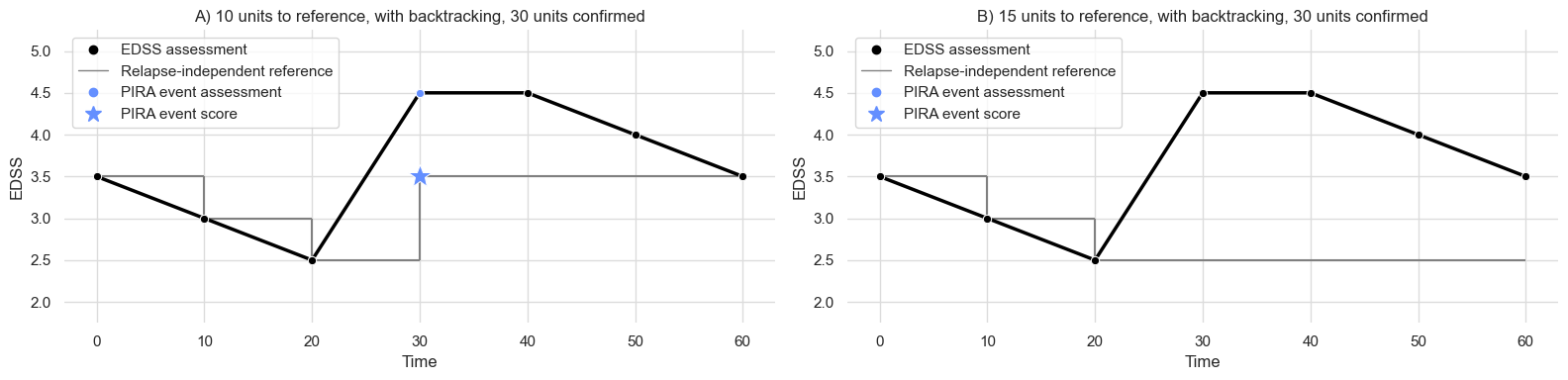


**eFigure 70**: Minimal distance to reference with backtracking and confirmation over 30 units, minimal increase + 1.0. **A)** Minimal distance 10 units, thus the score 2.5 at step 20 is the reference for the increase at step 30. The confirmation scores at steps 40, 50, and 60 all satisfy the confirmation condition score ≥ 2.5 + 1, thus the increase at step 30 is confirmed disability accrual. **B)** Minimal distance 15 units, thus the reference for the increase is the assessment at step 10 with score 3.0. Since the assessment at step 60 does not satisfy the confirmation condition score ≥ 3.0 + 1, the increase at step 30 is not confirmed.

Note that backtracking does not extend beyond past events (see eFigure 77 in the post-event re-baselining section) or beyond post-relapse re-baselining events (see eFigure 95 in the post-relapse re-baselining and minimal distance section).

## Baselines

### Baseline types

The algorithm supports two types of baselines: fixed baseline and roving reference.

The **fixed baseline** option uses the first assessment (“study baseline”) as reference for disability accrual, irrespective of potential improvement (eFigure 71A). The study baseline *r_0_* is the score measured at the first assessment of a connected follow-up, i.e. *r_0_ = s_0_*. When assessing disability accrual with respect to fixed baseline, the reference at the *i*-th assessment is thus *r_i_* = *r_0_ = s_0_*.

The **roving reference** option adjusts the reference in case of an improvement, optionally with a confirmation condition for the improvement (eFigure 71B). If no confirmation is required (and in the absence of relapses, see Post-relapse re-baselining), the roving reference at the *i*-th assessment is the minimum of all previously measured scores, i.e. *r_i_ = min(s_0_, s_1_, ..., s_i - 1_)*. Note that the roving reference is only adjusted whenever a decrease in EDSS is observed, but not in case of an increase, since this could mask an event and lead to a loss of sensitivity.


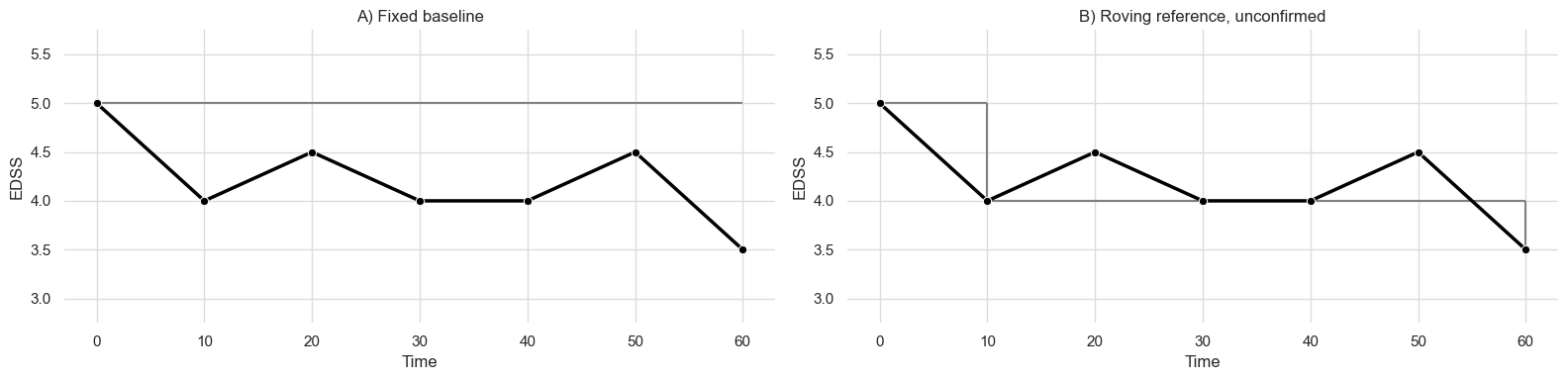


**eFigure 71**: **A)** Fixed baseline. **B)** Roving reference without confirmation requirement. The reference is reset at the improvements at steps 10 and 60. **Legend:** Black dots represent EDSS scores, and the grey curve represents the reference.

### Confirmation options for the roving reference

When using a roving reference, optional confirmation conditions can be required. A new reference is confirmed if all confirmation scores are smaller than the previous reference, and the new reference score is the maximum of the new reference candidate all confirmation scores. The **confirmation scores** for a potential new reference at time *t_i_* and a given confirmation interval *Δt* are either a) all scores *s_j_* at times *t_j_* with *t_i_* < *t_j_* ≤ *t_i_* + *Δt* ∪ min{*t_j_* | *t_j_* ≥ *t_i_* + Δt}, i.e. all scores within the confirmation time plus the first score *t_j_* that satisfies *t_j_* ≥ *t_i_* + Δt (“all”, eFigure 72A), or b) the score *s_j_* at time *t_j_* with *t_j_* = min{*t_j_* | *t_j_* ≥ *t_i_* + Δt}, i.e. the first score at or after the required confirmation time (“last”, eFigure 72B). The confirmation interval can be freely chosen. Choosing a confirmation interval smaller than the smallest time step in the data amounts to requiring confirmation at the next assessment (eFigure 72C). The “sustained” option is not available for roving reference confirmation since it would not allow any subsequent disability accrual events.


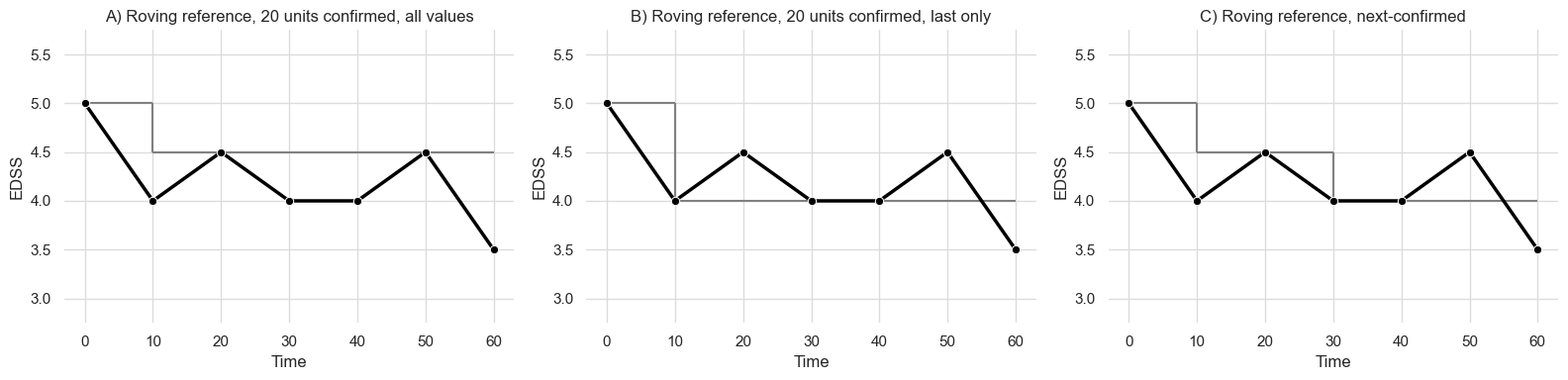


**eFigure 72**: **A)** Roving reference confirmed over a given time interval (20 units), all values included. The improvement at step 10 is confirmed at steps 20 and 30 with score 4.5, but the improvement at step 30 is not confirmed at step 50, thus it does not qualify as a new reference. **B)** Roving reference confirmed over a given time interval (20 units), last only. The confirmation assessment for the improvement at step 10 is the one at step 30 (the one at step 20 is ignored), thus the new reference is 4.0. **C)** Roving reference confirmed at the next assessment. The improvement at step 10 is confirmed at step 20, but only at 4.5, thus the new roving reference is 4.5. The improvement at step 30 is confirmed at step 40, thus the reference is reset again. The improvement at step 60 is unconfirmed. **Legend:** Black dots represent EDSS scores, and the grey curve represents the reference.

The algorithm supports left-hand tolerance for the confirmation distance (eFigure 73). It is equivalent to reducing the confirmation distance by the provided tolerance *only* if no right-hand side constraint (eFigure 74) is implemented (see Confirmation distance tolerances and constraints and eFigure 62).


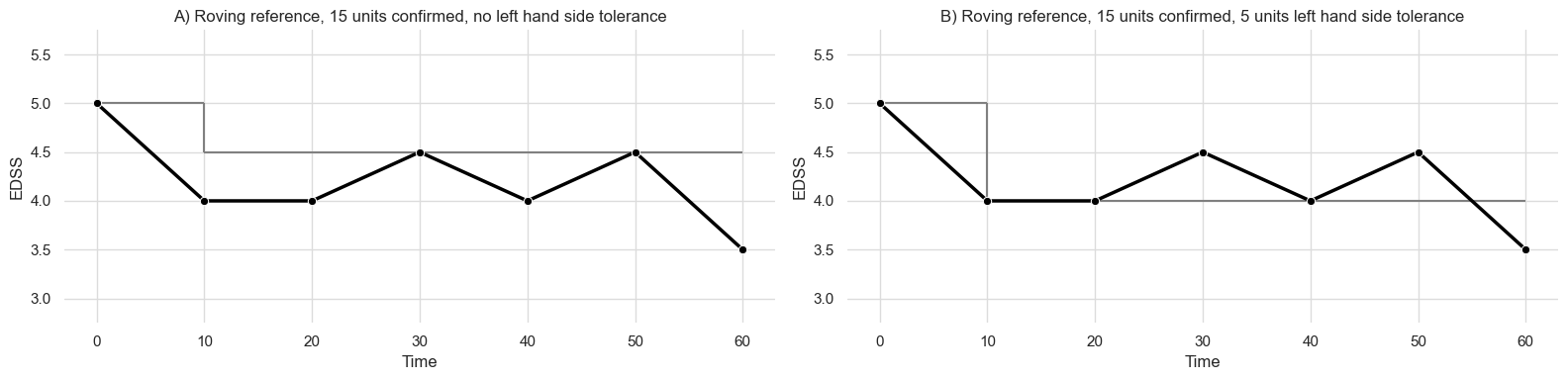


**eFigure 73**: Roving reference, 15 units confirmed, all values included. **A)** No tolerance, i.e. the first score that satisfies the confirmation distance condition for the improvement at step 10 is the one at step 30 (minimal timestamp *t_i_* + Δt – tolerance = 10 + 15 - 0 = 25). The confirmation scores are thus 4.0 (step 20) and 4.5 (step 30), and the new reference is thus 4.5. **B)** 5 units tolerance, i.e. the first score that satisfies the confirmation distance condition is the one at step 20 (minimal timestamp *t_i_* + Δt – tolerance = 10 + 15 - 5 = 20). The confirmation score and the new reference is thus 4.0. **Legend:** Black dots represent EDSS scores, and the grey curve represents the reference.

The algorithm supports right-hand constraints for the confirmation distance, i.e. it is possible to set a maximal distance between the required confirmation distance and the first score that satisfies the condition (eFigure 74).


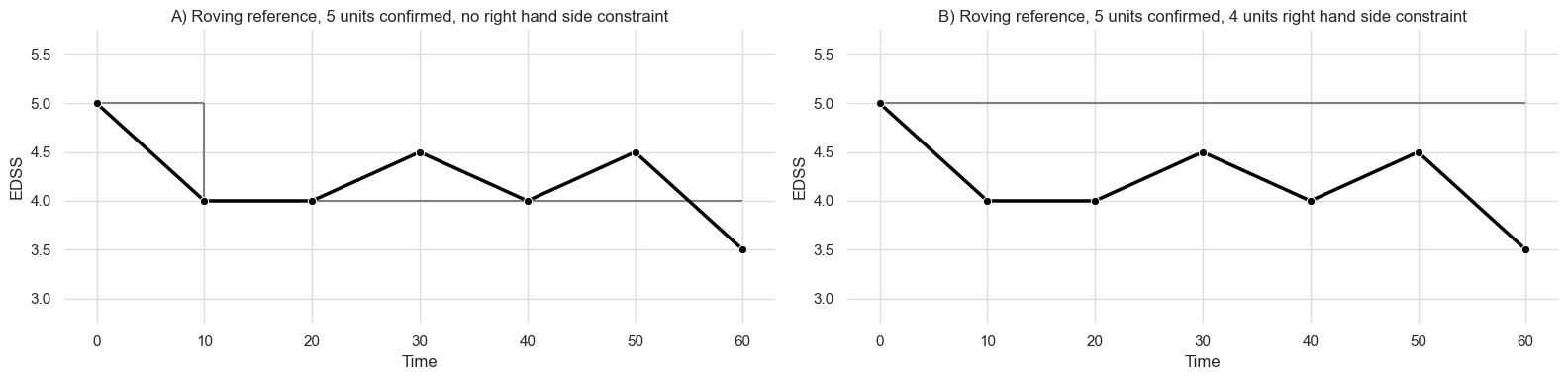


**eFigure 74**: Roving reference, 5 units confirmed, all values included. **A)** No right-hand constraint. **B)** Right-hand constraint of max. 4 units, thus the score at step 20 does not qualify as a confirmation score for step 10 (maximal timestamp *t_i_* + Δt + constraint = 10 + 5 + 4 = 19). **Legend:** Black dots represent EDSS scores, and the grey curve represents the reference.

## Post-event re-baselining

After each event, the baseline is reset to the confirmed event score (eFigure 75).


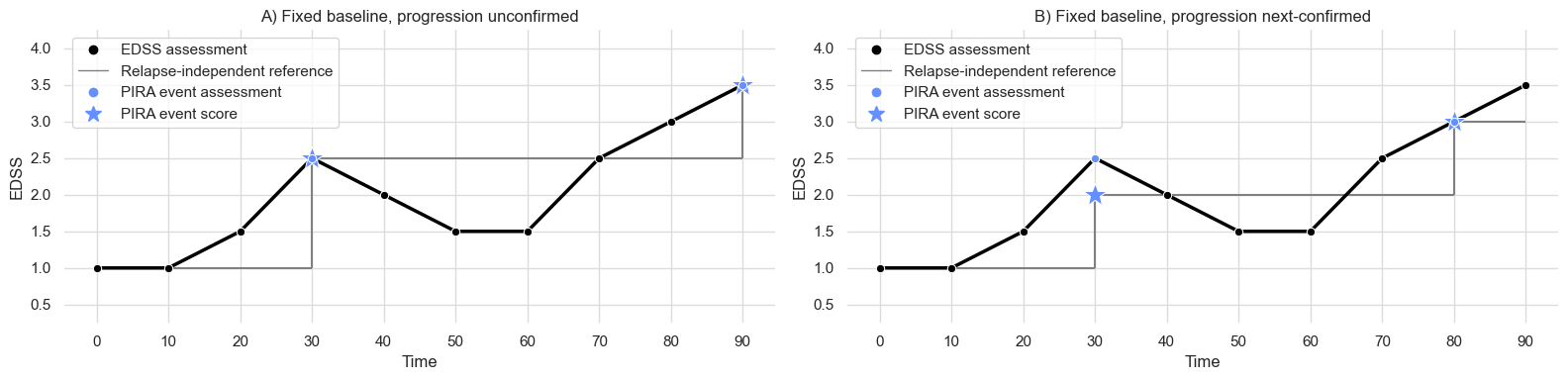


**eFigure 75**: Post-event re-baselining. After a disability accrual event, the reference is reset to the confirmed event score. **A)** No confirmation requirement, thus the event score of the PIRA event at day 30 is 2.5. **B)** The event must be confirmed at the next assessment, thus the confirmed event score is 2.0. Black dots represent EDSS scores, and the grey curve represents the reference.

### Post-event re-baselining and roving reference

If using a roving reference, the post-event reference can be reset if a subsequent assessment satisfies the criteria for a new roving reference (eFigure 76).


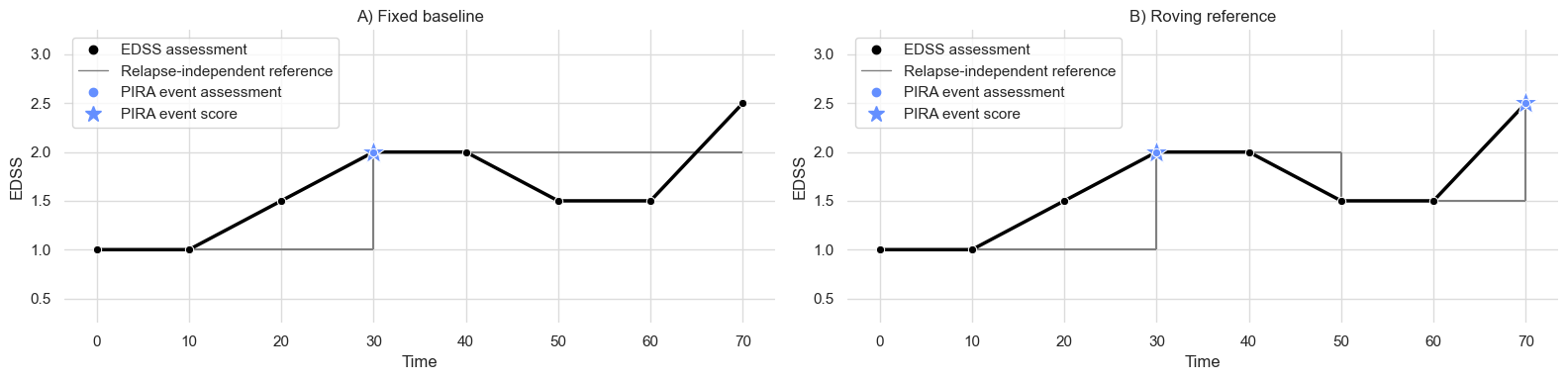


**eFigure 76**: Post-event re-baselining. **A)** Fixed baseline. The post-event reference stays at the confirmed event score 2.0. **B)** Roving reference. The score at step 50 qualifies as a new roving reference, thus the reference for subsequent event candidates is 1.5. Minimal increase + 1.0, next-confirmed roving reference, no confirmation for disability accrual required.

### Post-event re-baselining, roving reference, and minimal distance to reference

When requiring a minimal distance to reference and allowing backtracking, the backtracking only goes back to (and including) the last event, i.e. references recorded before an event are irrelevant as references for subsequent events (eFigure 77).


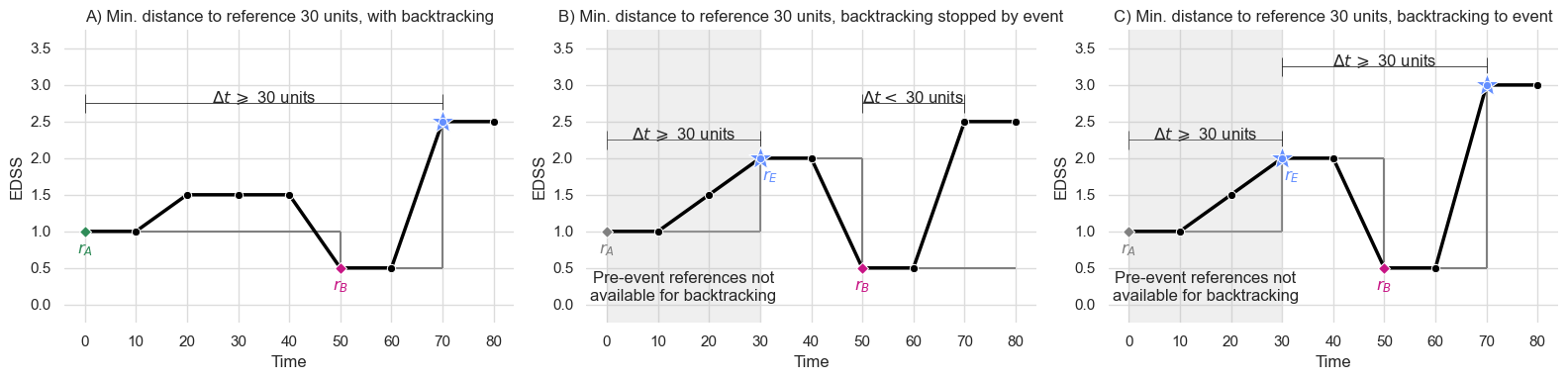


**eFigure 77**: Minimal distance to reference 30 units with backtracking, next-confirmed event, minimal increase over reference + 1.0. **A)** The new reference *r_B_* at step 50 is too close to the increase at step 70, but backtracking allows using the previous reference *r_A_*, thus the increase at step 70 qualifies as an event. **B)** The increase at step 30 qualifies as confirmed disability accrual with respect to the initial baseline *r_A_*, thus the baseline is set to the confirmed event score 2.0. The references available for the increase at step 70 are *r_B_* and *r_E_* (the reference set at the event), but *r_B_* is too close and *r_E_* is not low enough, thus the increase at step 70 does not qualify as an event. **C)** The increase at step 30 qualifies as confirmed disability accrual with respect to the initial baseline *r_A_*, thus the baseline is set to the confirmed event score 2.0. The references available for the increase at step 70 are *r_B_* and *r_E_* (the reference set at the event). While *r_B_* is too close, *r_E_* satisfies the minimal distance condition and is low enough, thus the increase at step 70 qualifies as an event. **Legend:** Black dots represent EDSS scores, blue stars represent PIRA event scores, blue dots represent PIRA event assessments, and the grey curve represents the reference. The grey shaded area marks assessments not available as references for assessments at times > 30.

## Event merging

Sequential events of the same type (typically PIRA, in case of a monotonic disability accrual) can be either annotated as a series of single events (eFigure 78A) or merged into one event with a correspondingly higher event score (eFigures 78B, 78C). Events are merged if an event’s confirmed score is greater than the previous event’s confirmed score. The following subsections provide an overview of merging options and the effects of combining confirmation requirements with event merging.

### Maximal distance

The algorithm supports maximal merge distance constraints (eFigure 78B). Events are only merged if they are within this maximal merge distance. Setting the maximal merge distance to ∞ allows event merging irrespective of distance.


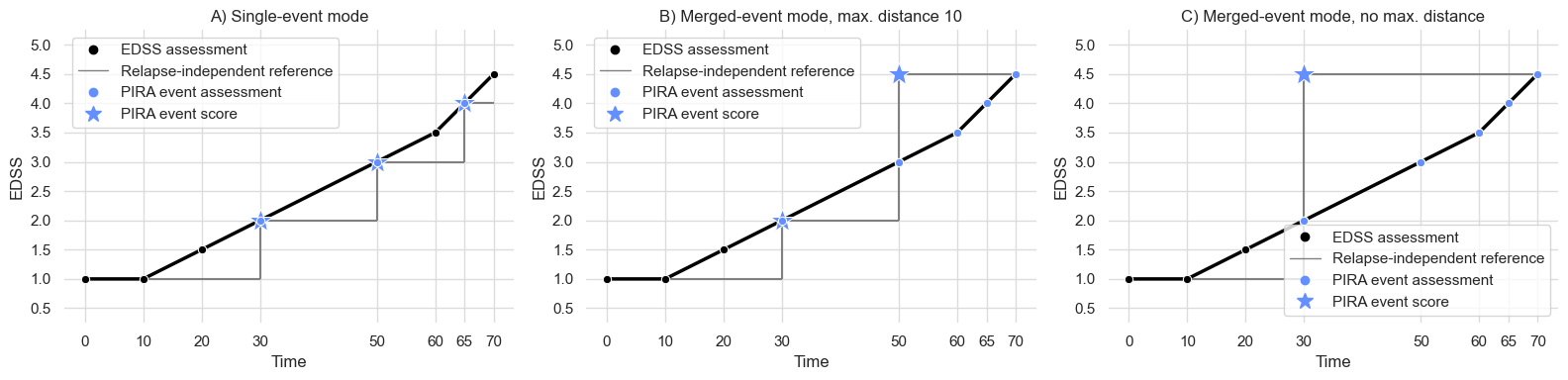


**eFigure 78**: Examples for merging events. **A)** Single-event mode, i.e. no merging. Three events with an event ΔEDSS of 1.0 each. **B)** Merged mode with a maximal merge distance of 10 units. Two events, one with event ΔEDSS 1.0, one with event ΔEDSS 2.5 points. **C)** Merged mode without maximal merge distance. One event with ΔEDSS 3.5 points.

### Maximal repetition distance

The algorithm supports maximal repetition distance constraints, i.e. sequential assessments with the same score can be either treated as stabilization if they happen outside the maximal repetition distance (ends the merging) or as repetition assessment if they happen within the maximal repetition distance (merging continues) (eFigure 79).


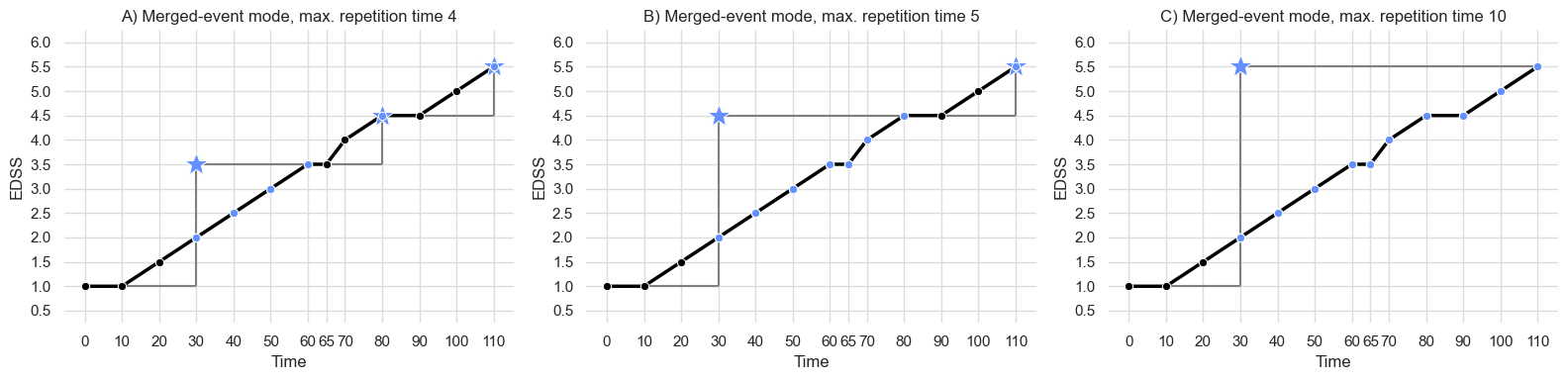


**eFigure 79**: Maximal repetition time. **A)** Assessments with a distance > 4 units are treated as stabilization, thus the event merging ends at step 60. **B)** Assessments with a distance > 5 units are treated as stabilization, thus the event merging ends at step 80. **C)** Assessments with a distance > 10 units are treated as stabilization, thus all events are merged into one. **Legend:** Black dots represent EDSS scores, blue stars represent PIRA event scores, blue dots represent PIRA event assessments, and the grey curve represents the reference.

The maximal repetition time is computed with respect to the last event, i.e. in case of multiple assessments with the same score as the event’s confirmed score, each of them must be within the maximal repetition time for the merging to continue (eFigure 80).


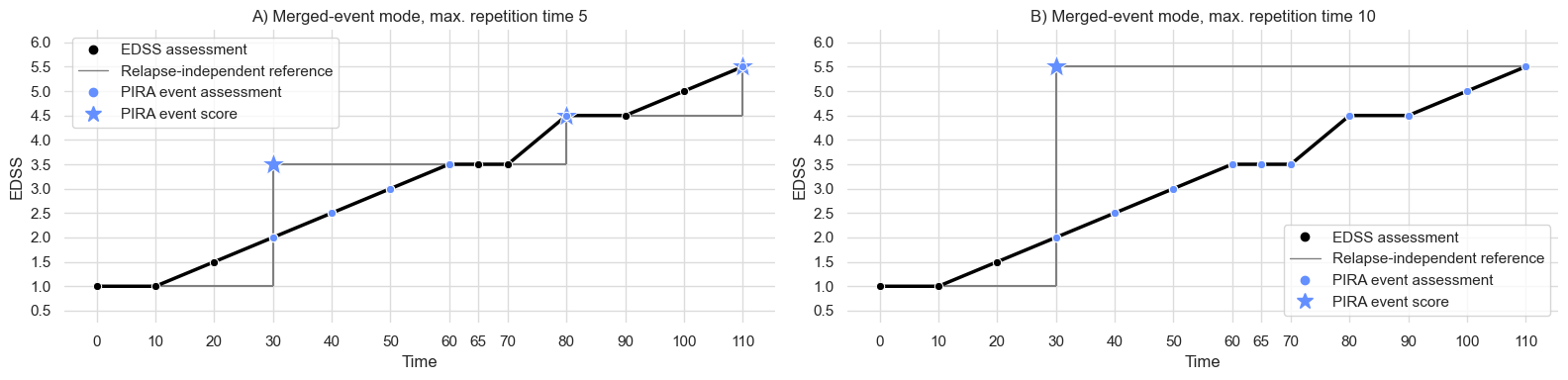


**eFigure 80**: Event merging. **A)** Max. repetition time 5 units. The assessment at step 65 qualifies as a repetition measurement for the event at step 60, but the assessment at step 70 does not, thus the merging process ends. **B)** Max. repetition time 10 units. The assessments at steps 65 and 70 both qualify as repetition measurement, and the next score is again an increase with respect to the previous confirmed score, so the merging continues.

Repetition measurements that are not followed by another increase are not included in the merged event (eFigure 81).


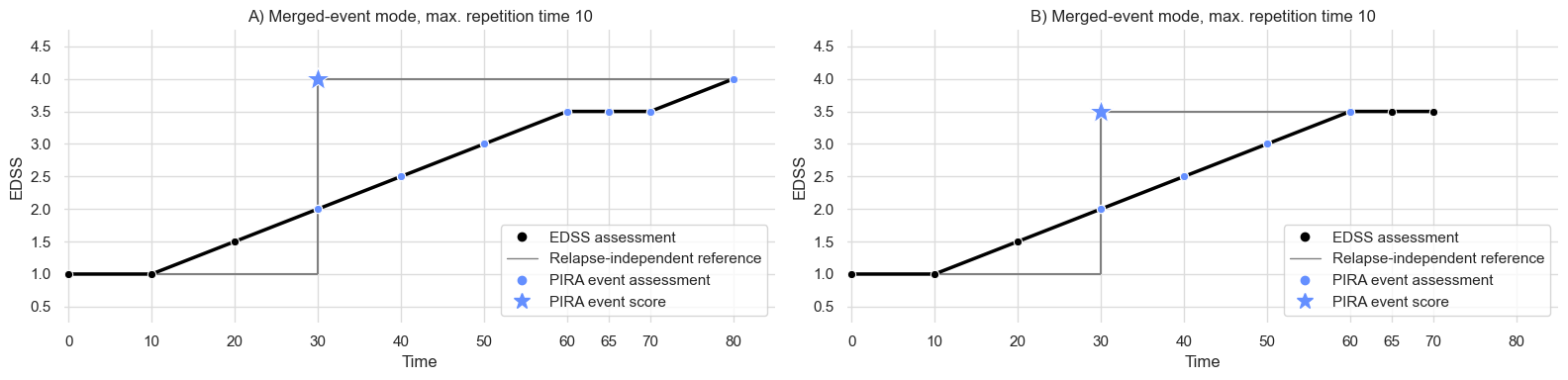


**eFigure 81**: Event merging, max. repetition time 10 units. **A)** The assessments at steps 65 and 70 both qualify as repetition measurement, and the score at the next assessment is again an increase with respect to the previous confirmed score, so the merging continues. **B)** The assessments at steps 65 and 70 both qualify as repetition measurement, but they are not followed by an increase, so the merging terminates at the last increase at step 60.

### Short-term improvements

Event merging either stops in case of a stagnation (previous section) or if a score’s confirmed score is lower than the previous confirmed event score. If no confirmation is required, this means that even a short-term improvement ends the event merging (Figure 82A). If event confirmation is required, an event’s *confirmed* score could be lower than the event’s score, thus a short-term improvement back to the confirmed score does not end the merge (if within the maximal repetition distance) (Figure 82B). A short-term improvement below the previous confirmed event score does end the merge (Figure 82C).


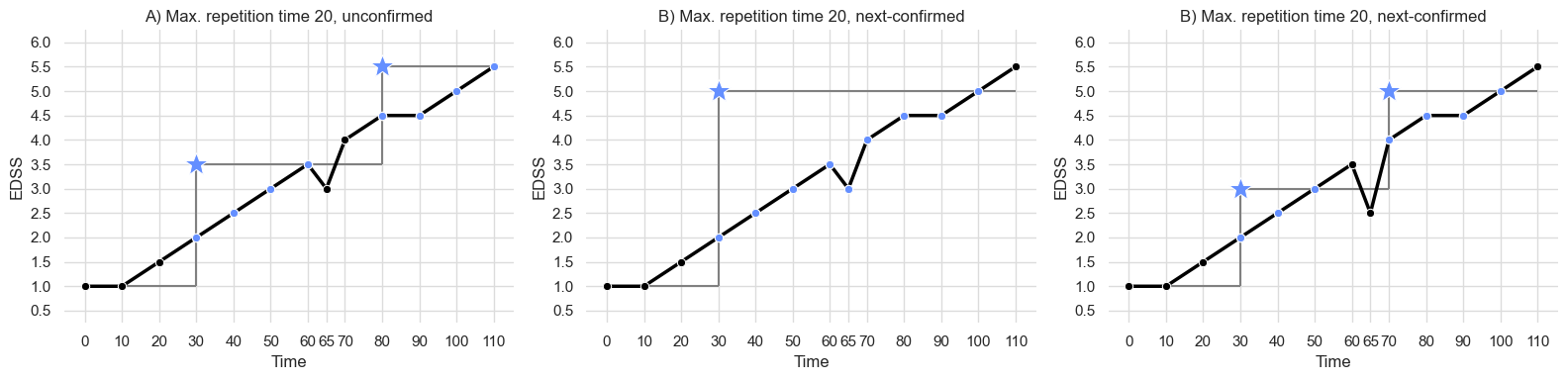


**eFigure 82**: Event merging, max. repetition distance 20 units. **A)** Unconfirmed disability accrual. The confirmed score at step 60 is thus 3.5, and the merging stops because the following score is lower. **B)** Next-confirmed disability accrual. The confirmed score at step 60 is 3.0, thus the event at step 65 with confirmed score 3.0 is considered a repetition measurement and the merging continues. **C)** The score at step 65 is lower than the confirmed score from step 60, so the merging is terminated. **Legend:** Black dots represent EDSS scores, blue stars represent PIRA event scores, blue dots represent PIRA event assessments, and the grey curve represents the reference.

Another example is shown in eFigure 83, where a score is equal to the previous confirmed event score. The merge only continues if the *confirmed* event score of this assessment also is equal to the previous confirmed event score.


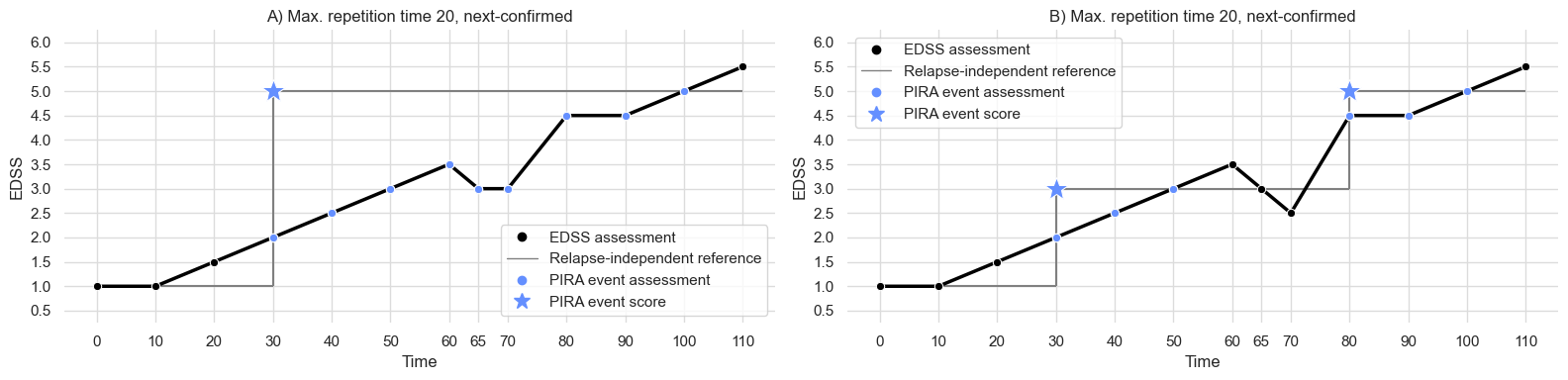


**eFigure 83**: Event merging, max. repetition distance 20 units, next-confirmed disability accrual. **A)** Next-confirmed disability accrual. The confirmed score at step 60 is 3.0, thus the events at steps 65 and 70 with confirmed score 3.0 are considered repetition measurements and the merging continues. **B)** The confirmed event score at step 65 is 2.5. This is lower than the confirmed score from step 60, so the merging is terminated.

### Short-term improvements with all vs. last confirmation

Choosing “all” vs. “last” confirmation requirements (see Confirmation interval) can affect event merging (eFigure 84). Since “last” confirmation leads to equal or greater confirmed scores compared to “all” confirmation, short-term improvements are more likely to terminate the merging process when using “last” confirmation.


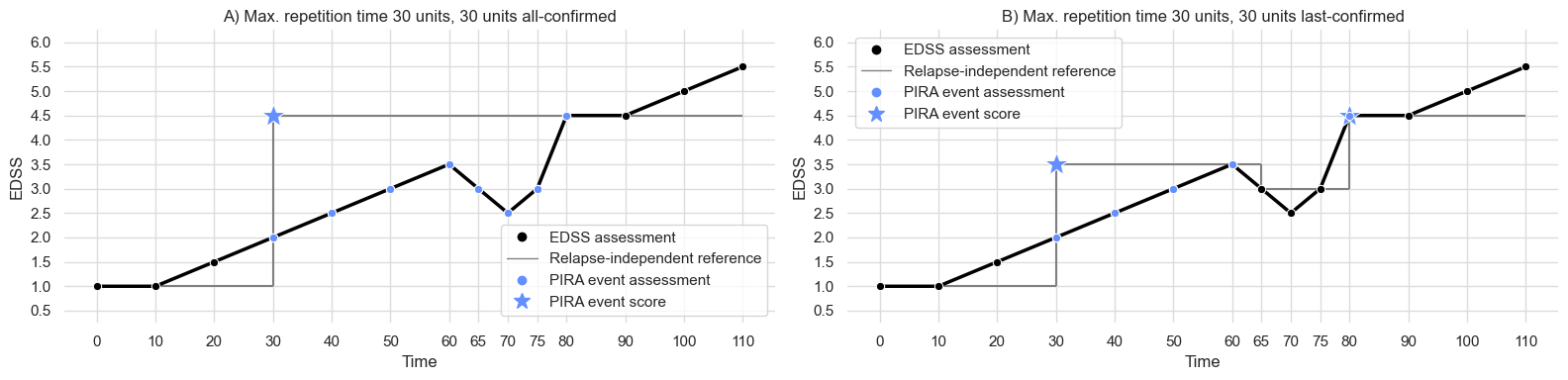


**eFigure 84**: Event merging, max. repetition distance 20 units, 30 units confirmed disability accrual. **A)** Confirmation for all values within the confirmation interval. The confirmed score at step 50 is 2.5, and it is considered a repetition measurement of the score at step 40 because it is within the maximum repetition distance. The same is true for the confirmed scores at steps 60, 65, and 70. The confirmed score at step 75 is 3.0, so the merging continues. **B)** Confirmation only for the first value that satisfies the minimal confirmation distance. The confirmed event score at step 60 is 3.5, since the corresponding confirmation assessment is the one at step 90. The following score is lower, thus the merging is terminated.

# Relapse-related definition aspects

This section covers all aspects of defining disability accrual events that are related to relapses, i.e. event type classification, the distance to relapses for Relapse Associated Worsening (RAW) or PIRA, the effect of relapses on confirmation conditions, minimal distance conditions, references, and event merging.

## Event types

### RAW and PIRA

An event is classified as **RAW** if it i) occurs in proximity of a relapse, e.g. within 30 days prior until 90 days after a relapse (within the “RAW window”) and ii) with respect to the post-relapse adjusted reference (“RAW/PIRA reference”, see Re-baselining rules section), and ii) if it is not a post-relapse re-baselining assessment (the first assessment after the RAW window) (eFigure 85A). An event is classified as **PIRA** if i) it occurs outside the RAW window of a relapse and ii) with respect to the RAW/PIRA reference, iii) it is not a post-relapse re-baselining assessment, and iv) if the confirmation assessments – if required – are not in a RAW window (eFigure 85B).


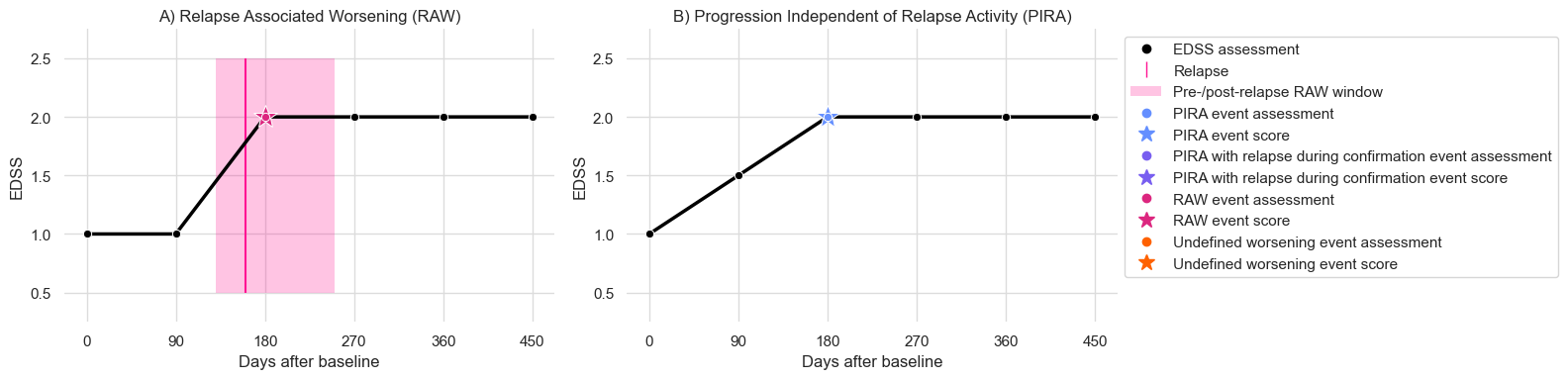


**eFigure 85**: Examples of **A)** RAW and **B)** PIRA. Black dots represent EDSS scores, the pink vertical line indicates a relapse, and the pink shaded area indicates the RAW window, the period in which an event is considered relapse-associated (typically 30 days before until 90 days after a relapse). Minimal required increase + 1.0 point on the EDSS scale.

### PIRA with relapse during confirmation and Undefined Worsening

An event that occurs outside the RAW window and with respect to the RAW/PIRA baseline and is not a re-baselining assessment but that is confirmed in proximity of a relapse is classified as **PIRA with relapse during confirmation** (eFigure 86A). An event that fulfills neither the RAW nor PIRA criteria is classified as **Undefined** **Worsening** (eFigure 86B). This happens e.g. if a post-relapse re-baselining assessment is itself a disability accrual event. In contrast to PIRA with relapse during confirmation, undefined worsening events are an issue of follow-up quality, usually due to missing assessments in proximity of a relapse.


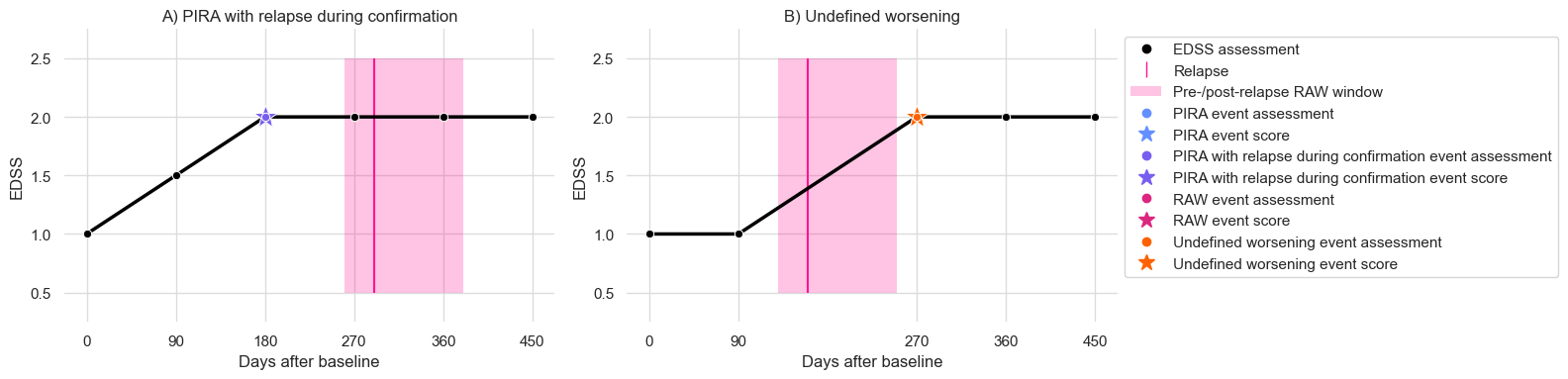


**eFigure 86**: Examples of **A)** PIRA with relapse during confirmation and **B)** undefined worsening. In A) the confirmation assessment is within the RAW window of the next relapse. The event at day 180 does thus not fulfill PIRA criteria, but cannot be considered RAW, either. It is thus labeled as *PIRA with relapse during confirmation*. In B) the post-relapse re-baselining assessment is itself an event. It is not possible to classify the event at day 270 as either PIRA or RAW (without any assessments in the RAW window, it is not possible to determine whether the increase happened before, during, or after), thus it is labeled as *Undefined Worsening.*

### RAW/PIRA reference and relapse-independent reference

Undefined events can also happen in case of a sequence of relapses which by themselves do not result in a sufficient EDSS increase to qualify as RAW (eFigure 87). To account for such cases, the algorithm keeps track of two references, a relapse-independent one that is only reset in case of an event and that is used to identify undefined events, and a relapse-dependent one that is reset after an event and in case of post-relapse residual disability and that is used to identify RAW/PIRA (see Post-relapse re-baselining section). **RAW/PIRA always take precedence over undefined**, i.e. an event can only be classified as undefined if it does not classify as RAW or PIRA (see the Undefined events section for further details on what events can qualify as undefined worsening).


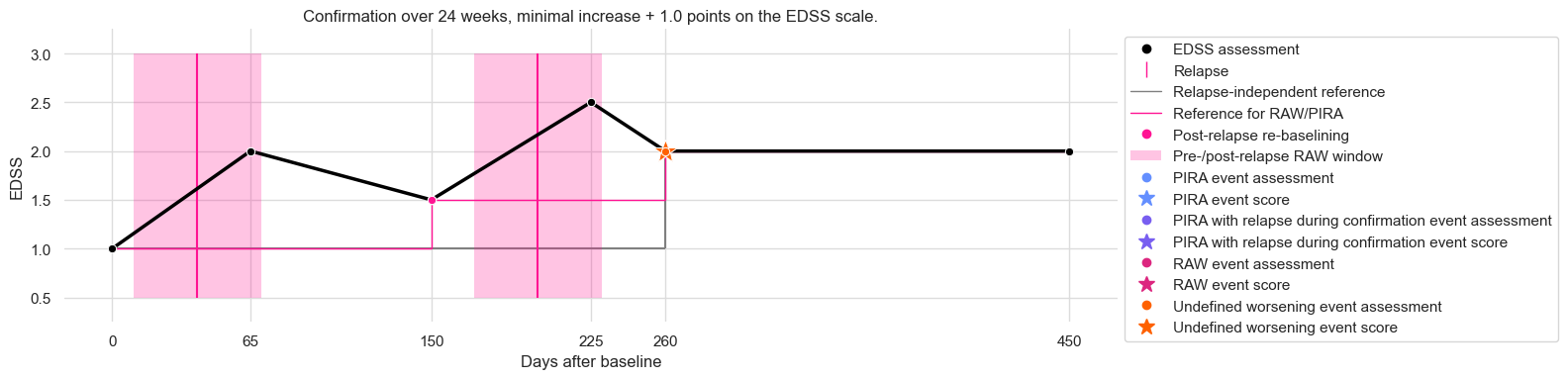


**eFigure 87**: Minimal increase for disability accrual + 1.0, event must be confirmed at the next assessment. The first relapse does not lead to confirmed RAW (the confirmation score at day 150 is too low), and the second relapse also does not lead to confirmed RAW with respect to the first assessment after the previous relapse (which is considered the post-relapse re-baselining assessment). The assessment at day 260 is, however, a large enough increase over the initial pre-relapse baseline. Therefore, the disability accrual observed at day 260 is classified as undefined.

### Relapses and event confirmation

The confirmation assessment(s) for a RAW event are not subject to any restrictions with respect to subsequent relapses, i.e. they may or may not be within the RAW window of a subsequent relapse. For PIRA, if a confirmation assessment is within the RAW window of a relapse, the event is labeled as PIRA with relapse during confirmation instead of as unambiguous PIRA. In addition, when using the “all” option for confirmation (see Confirmation interval section), if there is a relapse at any time within the confirmation interval, the event is also labeled as PIRA with relapse during confirmation. When using the “last” option for confirmation (see Confirmation interval section), relapses within the confirmation interval can optionally be allowed (eFigure 88A) or not allowed (eFigure 88B).


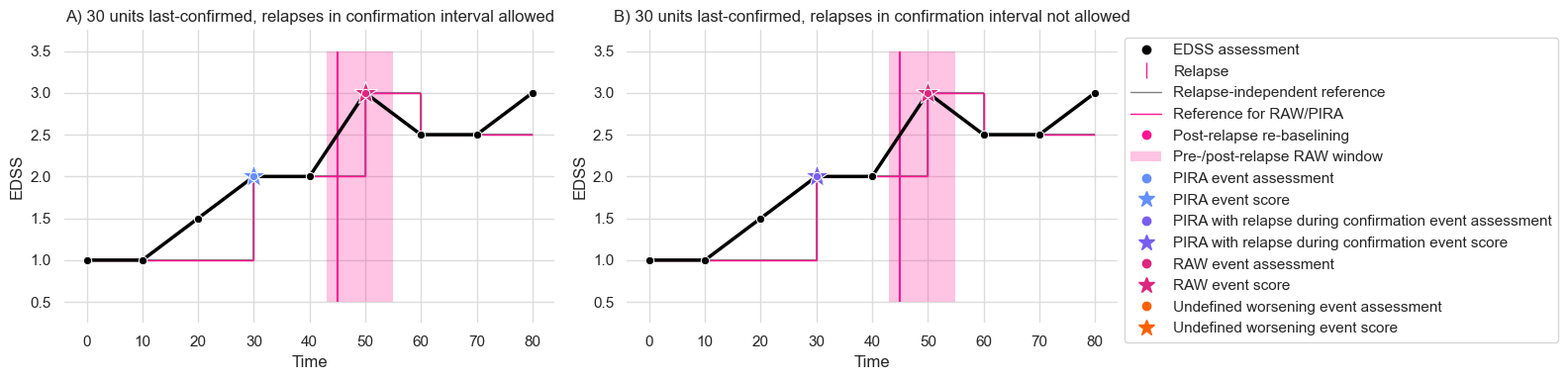


**eFigure 88**: “Last” confirmation over 30 units required, i.e. only the first assessment that satisfies the minimal confirmation time requirement is considered for confirmation. **A)** Relapses within confirmation interval allowed. The event at step 30 is confirmed by the assessment at step 60, which is not within a RAW window, thus the event is labeled as *PIRA*. **B)** Relapses within confirmation interval not allowed. Due to the relapse at step 45, which is within the confirmation interval, the event is labeled as *PIRA with relapse during confirmation*. Minimal required increase + 1.0 point on the EDSS scale.

Note that the switch from unambiguous PIRA to PIRA with relapse during confirmation is triggered by the presence of a relapse in the confirmation interval, irrespective of whether there is a confirmation assessment within the relapse’s RAW window (eFigure 89).


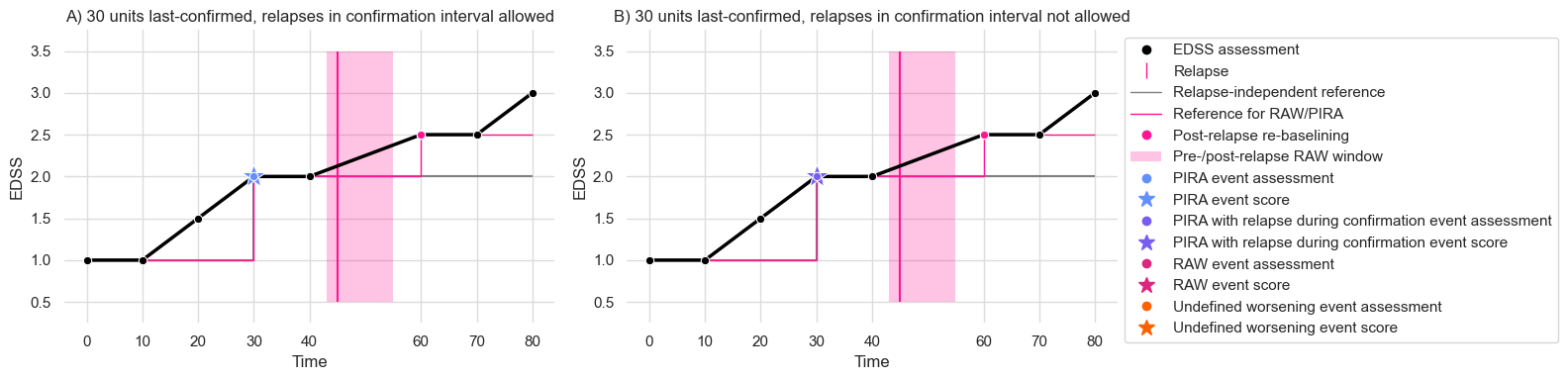


**eFigure 89**: Last” confirmation over 30 units required, i.e. only the first assessment that satisfies the minimal confirmation time requirement is considered for confirmation. **A)** Relapses within confirmation interval allowed. The event at step 30 is confirmed by the assessment at step 60, which is not within a RAW window, thus the event is labeled as *PIRA*. **B)** Relapses within confirmation interval not allowed. Due to the relapse at step 45, which is within the confirmation interval, the event is labeled as *PIRA with relapse during confirmation*. Minimal required increase + 1.0 point on the EDSS scale.

The requirement that the confirmation assessment itself must not be in a RAW window still applies even for “last” confirmation and relapses within confirmation interval allowed (eFigure 90).


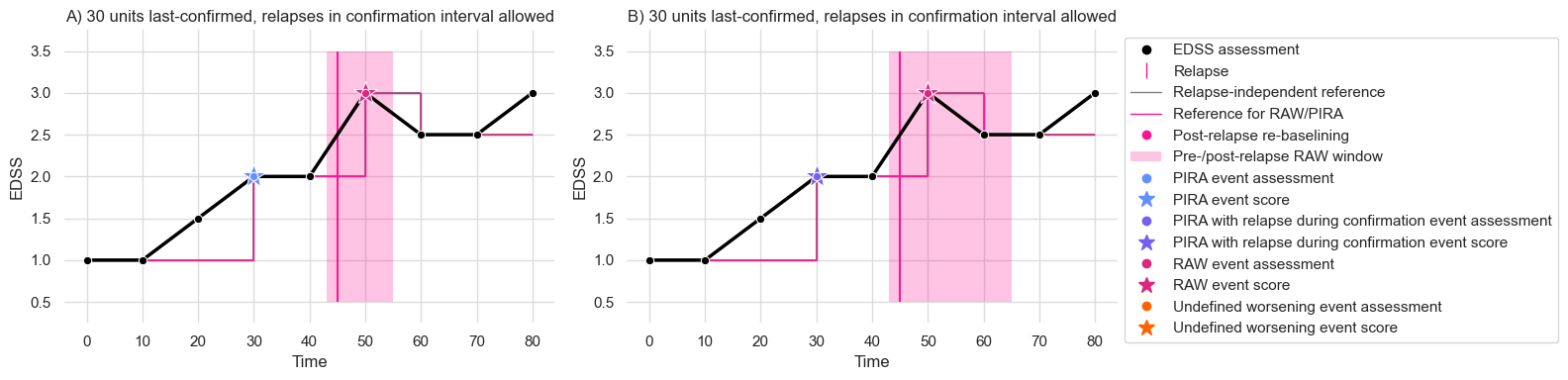


**eFigure 90**: “Last” confirmation over 30 units required, i.e. only the first assessment that satisfies the minimal confirmation time requirement is considered for confirmation, relapses within confirmation interval allowed. **A)** Post-relapse RAW window 10 units. The event at step 30 is confirmed by the assessment at step 60, which is not within a RAW window, thus the event is labeled as *PIRA*. **B)** Post-relapse RAW window 20 units. The event at step 30 is confirmed by the assessment at step 60, which is within a RAW window, thus the event is labeled as *PIRA with relapse during confirmation*. Minimal required increase + 1.0 point on the EDSS scale.

### Relapses, event types, and event merging

Events of different types, such as RAW and PIRA (eFigure 91), are never merged.


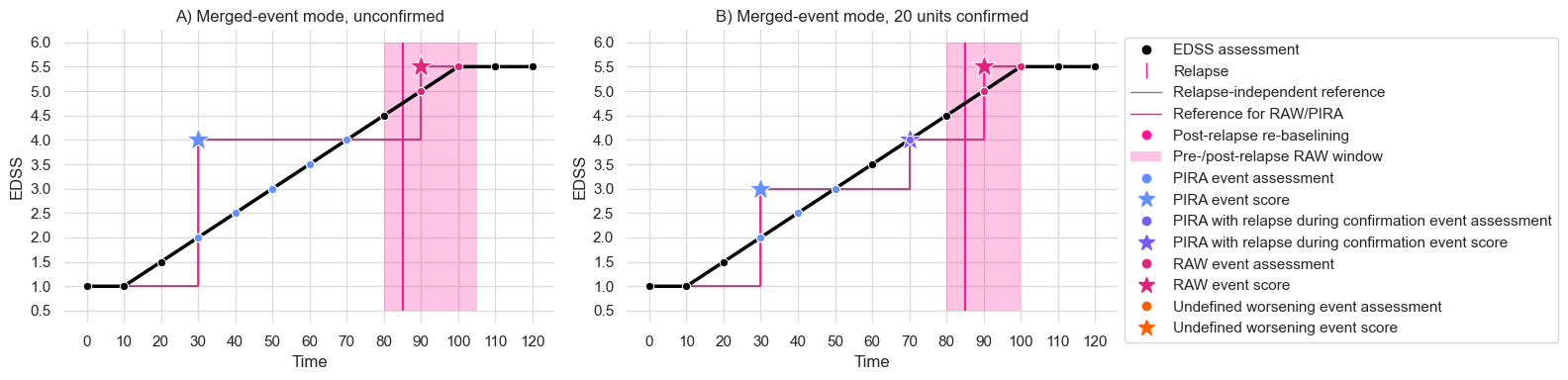


**eFigure 91**: Event merging, no maximal distance, minimal increase + 1.0 EDSS points. **A)** No confirmation required. The increases at steps 30 to 70 are all PIRA with respect to the baseline from step 0, thus they are merged into one PIRA event. The increases at steps 90 and 100 are RAW with respect to the post-event baseline from step 70 and thus merged. **B)** Confirmation at 20 units required. The increases at steps 30, 40, and 50 are PIRA, but the increase at step 60 is not because a confirmation assessment is in the RAW window of the subsequent relapse, thus the merging of PIRA events terminates after step 50. The increase at step 60 is not an event because it does not satisfy the minimal increase condition with respect to the post-event baseline from step 50. The increase at step 70 does and is classified as PIRA with relapse during confirmation. The increase at step 80 is already within the RAW window, thus it would not be of the same event type and the merging is terminated. The increases at steps 90 and 100 are RAW with respect to the post-event baseline from step 70 and thus merged.

Undefined events are also never merged (eFigure 92).


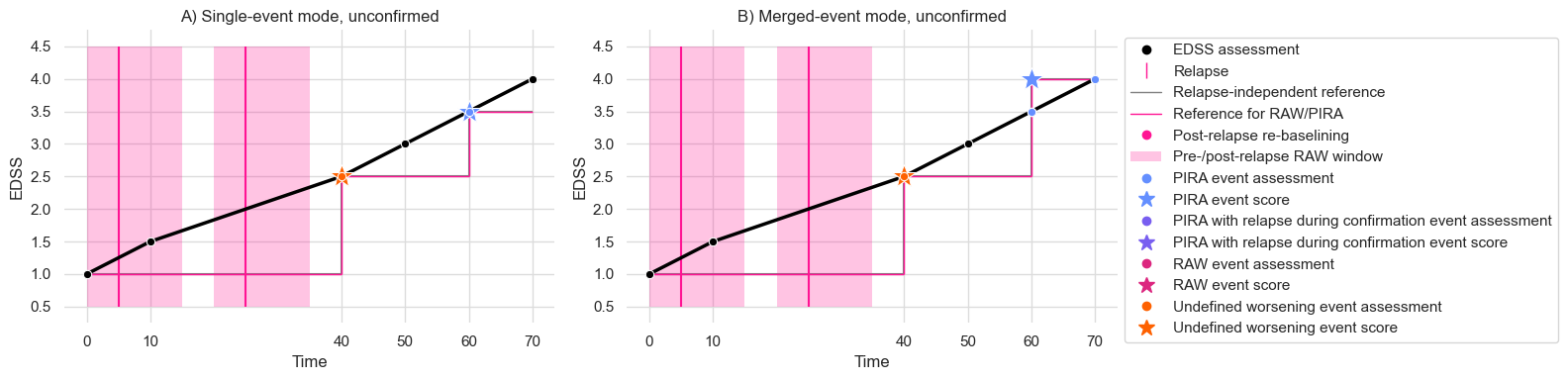


**eFigure 92**: Minimal increase + 1.0 EDSS points, no confirmation required. **A)** Single-event mode. The increase at step 40 is a post-relapse re-baselining assessment and thus undefined worsening. The increase at step 50 does not fulfill the minimum increase criterion with respect to the post-event baseline from step 40. The increase at step 60 is PIRA, and the increase at step 70 does not qualify as PIRA with respect to the post-event baseline from step 60. **B)** Merged-event mode. The increase at step 40 is a post-relapse re-baselining assessment and is thus labeled as undefined worsening. Undefined events are never merged because any following increase would be of another type (PIRA in this case). The increase at step 50 does not fulfill the minimum increase criterion with respect to the post-event baseline from step 40. The increases at steps 60 and 70 are PIRA with respect to the post-event baseline from step 40 and thus merged.

## Post-relapse re-baselining

### General rules

The baseline for RAW and PIRA is reset after a relapse if the relapse causes residual disability. This residual disability requires no confirmation (this is consistent with the determination of the study baseline, which also does not require confirmation). If the score at the post-relapse re-baselining assessment is equal to or lower than the previous reference, it remains unchanged (eFigure 93).


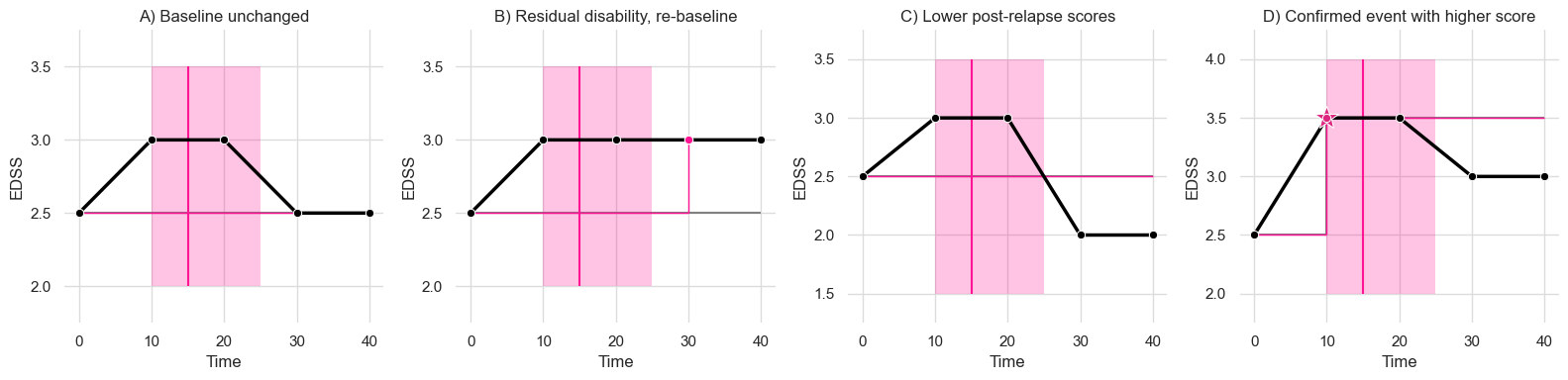


**eFigure 93**: Post-relapse re-baselining. **A)** The score drops to the pre-relapse reference, thus the reference remains unchanged. **B)** Residual disability, thus the reference is set to 3.0. **C)** The post-relapse score is lower than the previous reference, thus the reference remains unchanged (it could, however, be lowered to 2.0 when using a roving reference). **D)** The last confirmed event score is higher than the score at the post-relapse re-baselining assessment, thus the reference remains unchanged. **Legend:** Black dots represent EDSS scores, the pink vertical line indicates a relapse, the pink shaded area indicates the RAW window, pink dots indicate post-relapse re-baselining, red stars indicate RAW events, and the grey and pink curves represent the relapse-independent (for undefined) and relapse-dependent (for RAW/PIRA) references.

### Post-relapse re-baselining and events

Residual disability always takes precedence over confirmed scores. In case of a RAW event with a confirmed event score lower than the score at the following re-baselining, the RAW/PIRA reference is reset to the residual disability score (eFigure 94A). In case of an undefined worsening event with a confirmed score lower than the score recorded at the post-relapse re-baselining assessment, the score at the re-baselining assessment is kept as a new reference for RAW and PIRA (eFigure 94B).


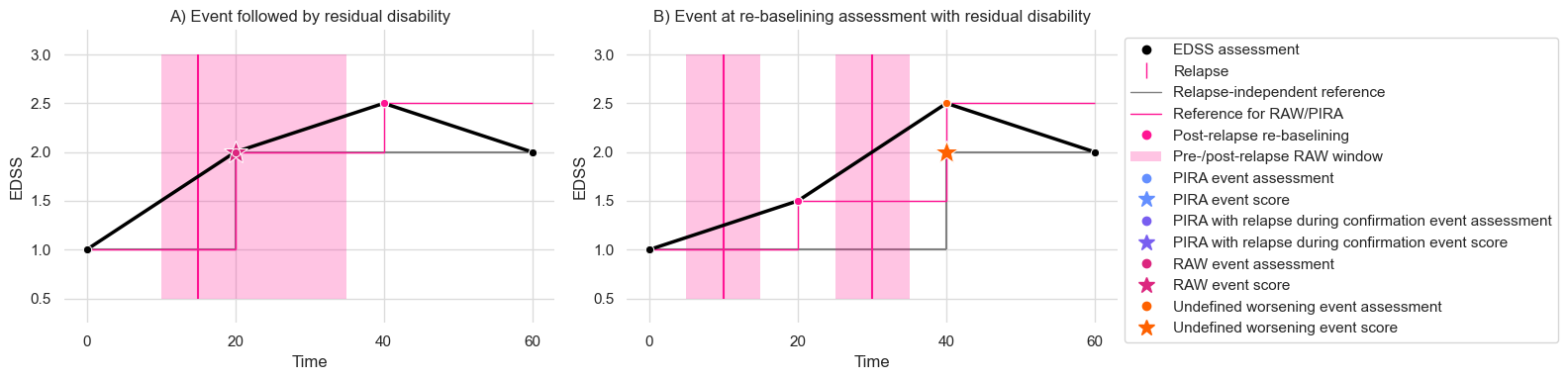


**eFigure 94**: Post-relapse re-baselining, special cases where the re-baselining coincides with an undefined worsening event. Confirmation at the next assessment required, minimal increase + 1.0. **A)** RAW event with an event score of 2.0, followed by residual disability. The RAW/PIRA reference is set to 2.5 at step 40 while the relapse-independent reference remains at the confirmed event score. **B)** The confirmed event score is lower than the post-relapse score, thus the reference for RAW/PIRA is higher than the confirmed score.

### Post-relapse re-baselining and minimal distance

Post-relapse re-baselining stops backtracking for RAW/PIRA if a minimal distance to reference is required (eFigure 95).


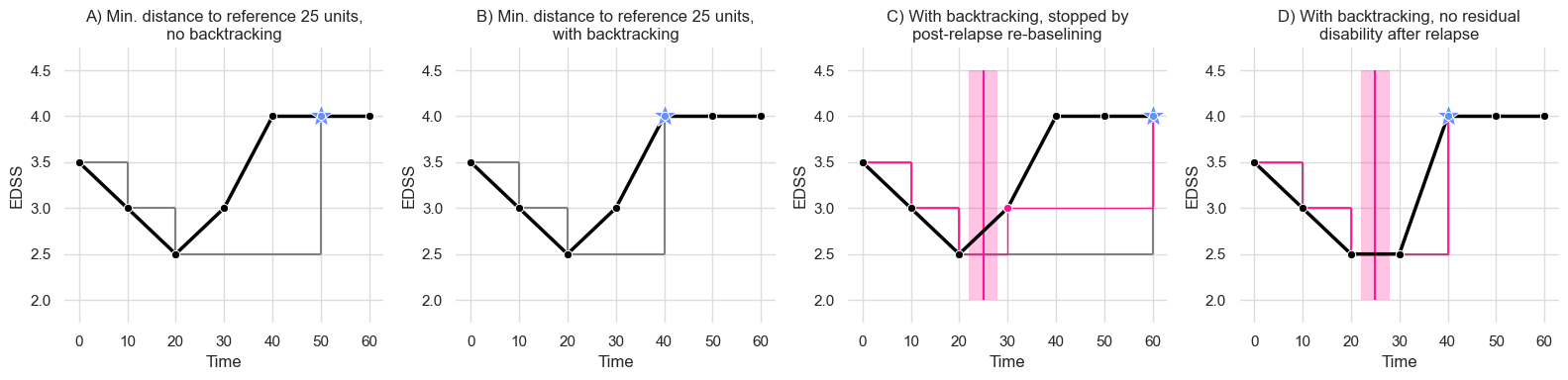


**eFigure 95**: Minimal distance to reference 25 units, minimal increase + 1.0, unconfirmed roving reference. **A)** No relapse, no backtracking. The assessment at step 40 does not satisfy the minimal distance condition, but the assessment at step 50 does, so it is classified as PIRA. **B)** With backtracking, the assessment at step 40 qualifies as PIRA because there is a reference low enough that satisfies the minimal distance condition (the assessment at step 10). **C)** With relapse and post-relapse re-baselining due to residual disability. After the relapse at step 25, the reference for RAW/PIRA is reset to 3.0, and backtracking past this assessment is not allowed, thus the first assessment that qualifies as PIRA is the one at step 60. **D)** No residual disability, therefore no post-relapse re-baselining, and backtracking past the relapse is allowed, thus the assessment at step 40 is PIRA. **Legend:** Black dots represent EDSS scores, the pink vertical line indicates a relapse, the pink shaded area indicates the RAW window, pink dots indicate post-relapse re-baselining, blue stars indicate PIRA events, and the grey and pink curves represent the relapse-independent (for undefined) and relapse-dependent (for RAW/PIRA) references.

### Multiple relapses

In case of multiple relapses, special rules for post-relapse re-baselining apply. The re-baselining assessment of one relapse can be within the RAW window of the next (eFigure 96B), coincide with the post-relapse re-baselining assessment of the next relapse (eFigure 96C), or RAW windows can overlap and be treated as one relapse (eFigure 96D).


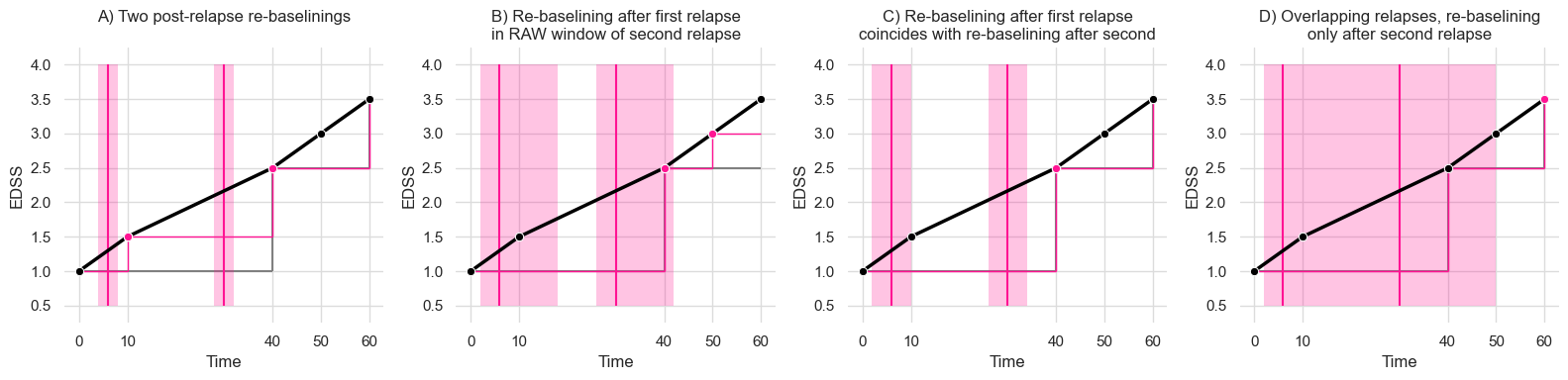


**eFigure 96**: Post-relapse re-baselining, **events not annotated**. Pink dots are post-relapse re-baselining assessments. **A)** Two relapses, each followed by an assessment after the RAW window and before the raw window of the next relapse. The assessments at steps 10 and 40 are post-relapse re-baselining assessments. **B)** Two relapses, where the first assessment after the RAW window of the first relapse is in the RAW window of the second relapse. The assessments at steps 40 and 50 are post-relapse re-baselining assessments. **C)** Two relapses, where the first assessment after the RAW window of the first relapse is after the RAW window of the second relapse. The assessment at step 40 is the post-relapse re-baselining assessment for both. **D)** Overlapping RAW windows, post-relapse re-baselining only at step 60. **Legend:** Black dots represent EDSS scores, the pink vertical line indicates a relapse, the pink shaded area indicates the RAW window, pink dots indicate post-relapse re-baselining, and the grey and pink curves represent the relapse-independent (for undefined) and relapse-dependent (for RAW/PIRA) references.

### Post-relapse re-baselining and ambiguous events

Note that the post-relapse re-baselining rules implemented to identify only unambiguous RAW or PIRA events may lead to at first glance counterintuitive behavior in certain special cases, such as undefined worsening events within the RAW window of a relapse (eFigure 97A, eFigure 97B). In eFigure 97A, the event at step 40 is categorized as undefined since there are no assessments after the first and before the second relapse, and it is thus impossible to exclude the possibility that the increase observed at step 40 had in fact happened between the relapses. In eFigure 97B, there is a series of relapses before the event, but there is also a relapse-free period. Without any assessments between steps 10 and 50, it is not possible to determine the origin of the disability accrual at step 50, and it is classified as undefined worsening. In eFigure 97C, there is no relapse-free period between steps 10 and 40, so the disability accrual at step 40 is unambiguously relapse-associated. The next event is undefined, though, because the last assessment within the RAW window at step 50 does not satisfy the minimal increase condition with respect to the post-event baseline from step 40, and the next increase can thus not be fully explained by the relapse.


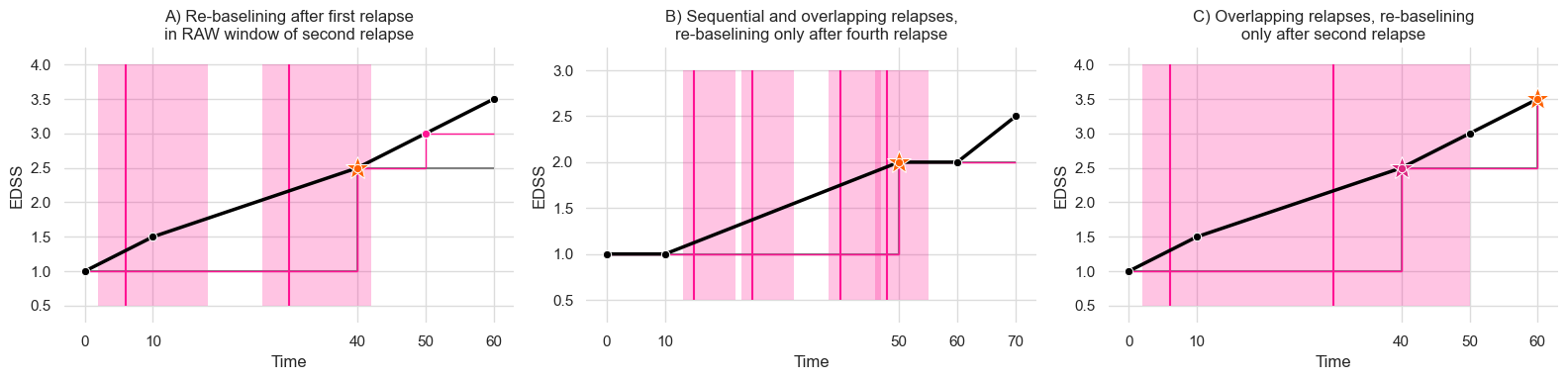


**eFigure 97**: Post-relapse re-baselining and events. Minimal increase + 1.0, no confirmation required. **A)** Two relapses, where the first assessment after the RAW window of the first relapse is in the RAW window of the second relapse. The assessments at steps 40 and 50 are post-relapse re-baselining assessments. The score at step 40 is a sufficient increase with respect to the baseline from step 0, and since it happens at a post-relapse re-baselining assessment, it is classified as undefined although it is in proximity to a relapse. From a clinical point of view this makes sense because it is not possible to exclude the possibility that the increase happened in the period between the two RAW windows. **B)** Sequential and overlapping relapses. The event at step 50 is undefined since it happens at the post-relapse re-baselining assessment for the first three relapses, which happens to fall within the RAW window of the last relapse. Note that the score at step 60 is not a post-relapse re-baselining because it is equal to the event score from step 50. **C)** Overlapping RAW windows, post-relapse re-baselining only at step 60. The increase at step 40 is RAW because it is in close temporal proximity to a relapse (within the RAW window) and not a post-relapse re-baselining assessment, and the increase at step 60 is undefined worsening. **Legend:** Black dots represent EDSS scores, the pink vertical line indicates a relapse, the pink shaded area indicates the RAW window, pink dots indicate post-relapse re-baselining, and the grey and pink curves represent the relapse-independent (for undefined) and relapse-dependent (for RAW/PIRA) references. Orange stars indicate undefined worsening events, red stars indicate RAW events.

## Undefined worsening

Undefined worsening events are increases a) with respect to the relapse-independent reference and b) that do not qualify as RAW, PIRA, or PIRA with relapse during confirmation (see Event types). By default, only post-relapse re-baselining assessments are checked for undefined worsening. Undefined worsening is usually an issue of follow-up quality, mostly due to too few assessments in proximity of relapses or between relapses. eFigure 98 shows examples of typical undefined worsening events.


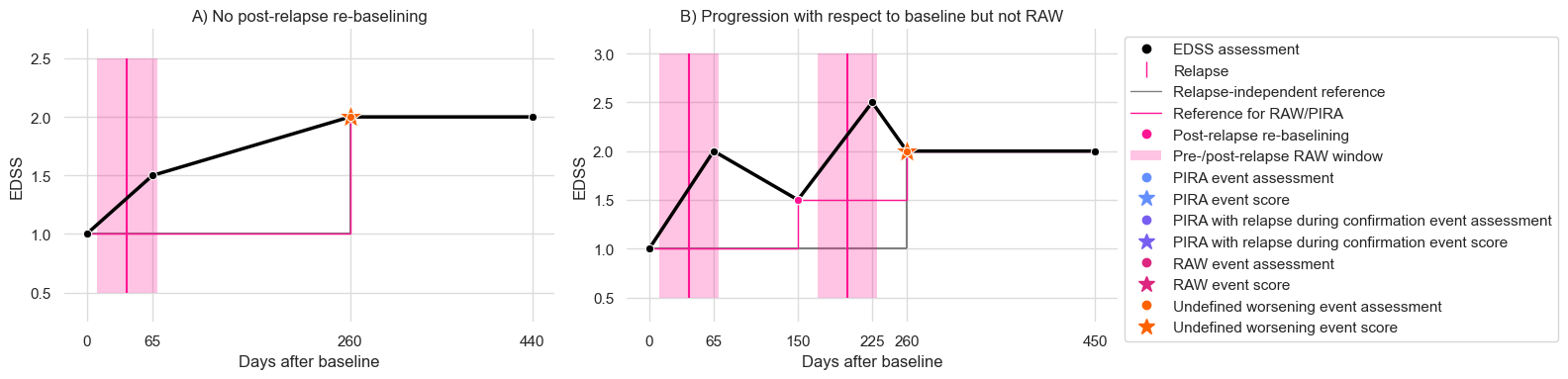


**eFigure 98**: Examples of undefined worsening. In case **A)**, the first assessment after the relapse is still within the RAW-window, and the post-relapse assessment is a disability accrual event. Case **B)** shows a sequence of relapses which by themselves do not result in a sufficient EDSS increase to qualify as RAW. The worsening after the first relapse is not confirmed, thus it is no RAW event. The reference for RAW/PIRA is then reset to 1.5 due to residual disability post-relapse, thus the worsening after the second relapse is not confirmed, either. It is, however, a disability accrual with respect to the original reference, and thus an undefined event.

### Options

The algorithm covers four options to annotate undefined worsening: A) Only post-relapse re-baselining assessments can be undefined worsening (“re-baselining only”, default option, eFigure 99A). All other significant (and optionally confirmed) increases with respect to the relapse-independent reference are ignored. B) Ignore the undefined worsening event category (i.e. re-baselining assessments are not checked for disability accrual, yields the same number of RAW/PIRA events as option A, but no undefined worsening events) (“never”, eFigure 99B). C) All assessments can be undefined worsening (after checking them for RAW/PIRA, so that RAW/PIRA events always take precedence over undefined) (“all”, eFigure 100A). D) Only post-relapse re-baselining assessments and assessments after the last RAW/PIRA event are checked for undefined worsening (“end”, eFigure 100B). For the “end” option, the annotation algorithm is applied twice, first with the “re-baselining only” option on the entire follow-up, and then with the “all” option of the remainder of the follow-up after the last RAW/PIRA event.


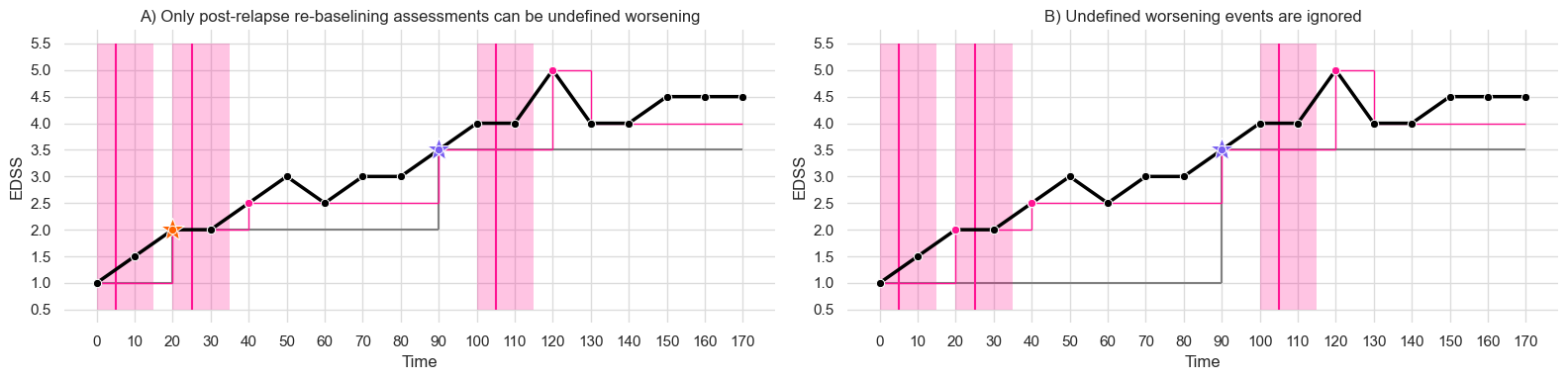


**eFigure 99**: Minimal increase + 1.0, confirmation at the next assessment required, roving reference. **A)** Only increases at post-relapse re-baselining assessments can be undefined worsening (“re-baselining only” option). The increase at step 20 is a post-relapse re-baselining assessment and a confirmed increase with respect to the initial baseline, therefore it is classified as undefined event with event score 2.0. The RAW/PIRA reference is reset at step 40 due to post-relapse residual disability after the second relapse. The increase at step 70 would be a significant confirmed increase with respect to the relapse independent baseline, but since it is not a post-relapse re-baselining assessment, it is not classified as undefined event. The increase at step 90 is a confirmed PIRA with relapse during confirmation event. The increase at step 120 is not confirmed, and the following assessments are neither post-relapse re-baselining assessments nor a significant increase with respect to the RAW/PIRA baseline. **B)** Undefined events are ignored (“never” option). Only the increase at step 90 is classified as event (PIRA with relapse during confirmation). **Legend**: Black dots represent EDSS scores, the pink vertical line indicates a relapse, the pink shaded area indicates the RAW window, purple stars indicate PIRA with relapse during confirmation events, orange stars indicate undefined worsening events, pink dots indicate post-relapse re-baselining assessments, and the grey and pink curves represent the relapse-independent (for undefined) and relapse-dependent (for RAW/PIRA) references.


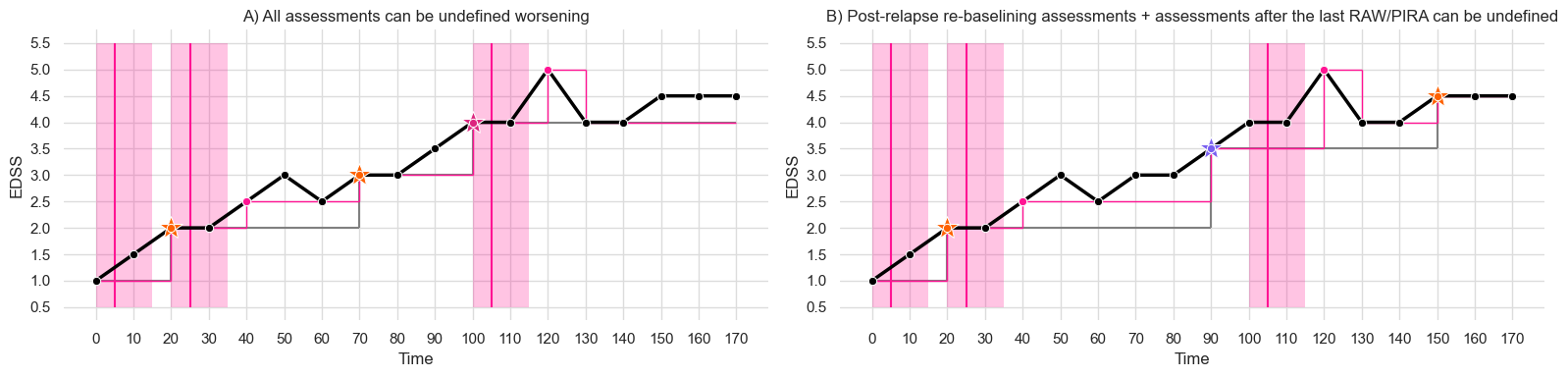


**eFigure 100**: Minimal increase + 1.0, confirmation at the next assessment required, roving reference. **A)** All events can be undefined worsening (“all” option). The increase at step 20 is a post-relapse re-baselining assessment and a confirmed increase with respect to the initial baseline, therefore it is classified as undefined event with event score 2.0. The RAW/PIRA reference is reset at step 40 due to post-relapse residual disability after the second relapse. The increase at step 70 is a significant confirmed increase with respect to the relapse independent baseline and thus classified as undefined event. Both references are set to 3.0, the confirmed event score. Thus, the increase at step 90 that qualified as PIRA with relapse during confirmation in the example in eFigure 99 does not fulfill the minimum increase condition anymore and is not classified as event. On the other hand, the increase at step 100 now fulfills the minimum increase condition with respect to both references, and since RAW/PIRA events take precedence over undefined events and it occurs within the RAW window of the third relapse, it is classified as RAW. **B)** Increases at post-relapse re-baselining assessments and all increases after the last PIRA/RAW event can be undefined worsening events (“end” option). The increase at step 20 is a post-relapse re-baselining assessment and a confirmed increase with respect to the initial baseline, therefore it is classified as undefined event with event score 2.0. The RAW/PIRA reference is reset at step 40 due to post-relapse residual disability after the second relapse. The increase at step 70 does not qualify as undefined worsening because a PIRA with relapse during confirmation event can be found at a later step (90). Since this is the last possible RAW/PIRA event, all further assessments can qualify as undefined worsening. After the third relapse, the RAW/PIRA reference is first set to 5.0 at step 120 due to post-relapse residual disability and then lowered to 4.0 at step 130 (roving reference while the relapse-independent reference stays at the confirmed event score of the last event. The increase at step 150 is a significant confirmed increase with respect to the relapse-independent reference but not with respect to the RAW/PIRA reference, thus it is classified as undefined worsening. **Legend**: Black dots represent EDSS scores, the pink vertical line indicates a relapse, the pink shaded area indicates the RAW window, red stars indicate RAW events, purple stars indicate PIRA with relapse during confirmation events, orange stars indicate undefined worsening events, pink dots indicate post-relapse re-baselining assessments, and the grey and pink curves represent the relapse-independent (for undefined) and relapse-dependent (for RAW/PIRA) references.

### Which option should I use for my endpoint?

Each of these options has advantages and drawbacks. The “re-baselining only” option focuses on the identification of all RAW and PIRA events while at the same time also detecting additional undefined events. While some disability accrual events might still remain undetected with this option (eFigure 101), the handling of undefined worsening events is consistent over the entire follow-up. Even more events that are not classifiable as PIRA or RAW remain undetected with the “never” option, however this is the most efficient option for when the endpoint is “first unambiguous RAW or PIRA event”.

**eFigure 101**: Minimal increase +1, event must be confirmed at the next assessment. Depending on the choice for the RAW window size, the overall number of events (irrespective of type) is different for the exact same follow-up when using “re-baselining” only. **A)** For a post-relapse RAW window of 30 days, the post-relapse re-baselining happens at day 85, and therefore the assessment at day 140 is checked for RAW/PIRA with respect to the new post-relapse baseline. In this case the increase is not large enough for an event, so no event is detected. **B)** The increase at day 140 is a post-relapse re-baselining assessment for a post-relapse RAW window of 90 days, and is thus labelled as undefined worsening.

The “all” option captures events at their earliest onset, but can mask subsequent RAW or PIRA events due to post-event re-baselining (eFigure 102A). This option is suitable for endpoints where the main interest is the detection of disability accrual events irrespective of type and as early as possible. The “end” option captures all events that are captured with the “re-baselining only” option plus potential additional events after the last RAW/PIRA, but the handling of undefined events is not consistent over the entire follow-up (eFigure 102B), and the partial re-running of the annotation algorithm makes this option the computationally heaviest one.

**eFigure 102**: Minimal increase +1, event must be confirmed at the next assessment. **A)** Allowing undefined worsening at each assessment leads to earlier detection of an event, but it can mask subsequent events due to post-event re-baselining. In this case, the undefined event at step 140 prevents the identification of a PIRA event at step 200 and yields a PIRA with relapse during confirmation event at step 230 instead. **B)** Only allowing undefined worsening after the last RAW or PIRA event captures all RAW/PIRA events and the overall disability accrual, but the handling of undefined worsening is inconsistent over the follow-up. Although both the increase at step 140 and the increase at step 380 are significant and confirmed with respect to the relapse-independent reference, only the latter is marked as a disability accrual event. **Legend**: Black dots represent EDSS scores, the pink vertical line indicates a relapse, the pink shaded area indicates the RAW window, blue stars indicate PIRA events, purple stars indicate PIRA with relapse during confirmation events, orange stars indicate undefined events, pink dots indicate post-relapse re-baselining assessments, and the grey and pink curves represent the relapse-independent (for undefined) and relapse-dependent (for RAW/PIRA) references.

### Counterintuitive events with the “all” option

As discussed in the Post-relapse re-baselining and ambiguous events section and shown in eFigure 97, undefined worsening can happen in the RAW window of a relapse in low-quality follow-ups. As discussed in the previous section, the “all” option captures events as early as possible at the cost of losing sensitivity for RAW/PIRA, thus undefined worsening in the RAW window of a relapse can also occur in high-quality follow-ups when using the “all” option for undefined (eFigure 103).

**eFigure 103**: Minimal increase + 1.0. **A)** Re-baselining only (default). The increase at step 10 is a confirmed RAW event, followed by a post-relapse re-baselining at step 20. The increase at step 30 cannot be undefined worsening and does not satisfy the minimum increase criterion with respect to the RAW/PIRA reference, thus it does not qualify as event. The increase at step 40 satisfies the minimum increase condition and is classified as RAW event. **B)** All events can be undefined worsening. The increase at step 10 is a confirmed RAW event, followed by a post-relapse re-baselining at step 20. The increase at step 30 does not satisfy the minimum increase criterion with respect to the RAW/PIRA reference, but with respect to the relapse-independent baseline, thus it is classified as a disability accrual event. The increase at step 40 does not satisfy the minimum increase criterion with respect to the new post-event baseline.

### Thresholds for undefined events

When requiring event confirmation in combination, it is possible that event candidates for undefined worsening have a lower event score than the current RAW/PIRA reference. The algorithm allows constraining undefined worsening events to only events with scores ≥ or > the current RAW/PIRA reference (eFigure 104, eFigure 105, eFigure 106). Note that undefined worsening events with event scores < the current RAW/PIRA baseline do not mask subsequent RAW/PIRA events since a) event scores must be ≤ the score at the event candidate’s assessment and b) undefined worsening events are never merged, thus post-event re-baselining after such an event never leads to a higher RAW/PIRA reference.

**eFigure 104**: Fixed baseline, minimal increase + 0.5, confirmation at the next assessment required. Thresholds for undefined worsening events. The increase at step 10 is not confirmed, but sets a new RAW/PIRA reference due to post-relapse residual disability. **A)** No constraints. The increase at step 40 is at a post-relapse re-baselining assessment and a confirmed increase with respect to the relapse-independent baseline and thus classified as undefined worsening. **B)** Only events with scores ≥ the current RAW/PIRA reference can be undefined worsening, so the increase at step 40 does not qualify. **C)** Only events with scores > the current RAW/PIRA reference can be undefined worsening, so the increase at step 40 does not qualify. **Legend**: Black dots represent EDSS scores, the pink vertical line indicates a relapse, the pink shaded area indicates the RAW window, blue stars indicate PIRA events, orange stars indicate undefined worsening events, pink dots indicate post-relapse re-baselining assessments, and the grey and pink curves represent the relapse-independent (for undefined) and relapse-dependent (for RAW/PIRA) references.

**eFigure 105**: Next-confirmed roving reference, minimal increase + 0.5, confirmation at the next assessment required. Thresholds for undefined worsening events. The increase at step 10 is not confirmed, but sets a new RAW/PIRA reference due to post-relapse residual disability. The decrease at step 30 is confirmed with a score of 6.5 at step 40, thus sets a new roving reference for RAW/PIRA, while the relapse-independent reference remains unchanged. **A)** No constraints. The increase at step 40 is at a post-relapse re-baselining assessment and a confirmed increase with respect to the relapse-independent baseline and thus classified as undefined worsening. **B)** Only events with scores ≥ the current RAW/PIRA reference can be undefined worsening, thus the increase at step 40 is an undefined worsening event. **C)** Only events with scores > the current RAW/PIRA reference can be undefined worsening, so the increase at step 40 does not qualify. **Legend**: Black dots represent EDSS scores, the pink vertical line indicates a relapse, the pink shaded area indicates the RAW window, blue stars indicate PIRA events, orange stars indicate undefined worsening events, pink dots indicate post-relapse re-baselining assessments, and the grey and pink curves represent the relapse-independent (for undefined) and relapse-dependent (for RAW/PIRA) references.

Undefined worsening events with an event score < the current RAW/PIRA reference can also occur when requiring a minimal increase of + 1.0 in case of a series of relapses that each lead to an unconfirmed increase with a partial recovery (eFigure 106).

**eFigure 106**: Fixed baseline, minimal increase + 1.0, confirmation at the next assessment required. The RAW/PIRA reference is adjusted by + 0.5 after each of the first three relapses. After the fourth relapse, the post-relapse re-baselining score stays at + 1.0 with respect to the relapse-independent reference for > 1 assessment, thus it is qualified as undefined worsening. The relapse-independent reference is set to 2.0 (the confirmed event score) while the RAW/PIRA reference stays at 2.5 (residual disability after the third relapse).

# Example of all four event types in one follow-up

**eFigure 107**: Example follow-up with all EDSS disability accrual types. Minimal required EDSS + 1.0, next-confirmed roving reference, events confirmed at the next assessment, RAW window 30 days pre-/post-relapse, default option for undefined worsening (re-baselining only), no minimal distance requirement, no event merging. The increase at day 60 is not confirmed, and the RAW/PIRA reference is reset at day 90 due to post-relapse residual disability. The increase at day 220 is not confirmed with respect to the RAW/PIRA reference, so it is not classified as RAW, and since it is not a post-relapse re-baselining assessment, it is not classified as undefined worsening either. The assessment at day 250 is a post-relapse re-baselining assessment (residual disability from the second relapse), and since it is a confirmed increase of + 1.0 over the relapse-independent reference it is classified as undefined worsening with confirmed event score 2.0, and both references are reset to 2.0 (post-event re-baselining). The improvement at day 430 is confirmed at day 490, thus both references are reset to 1.5 (roving reference). The increase at day 550 occurs in proximity of a relapse (within the relapse’s RAW window) and is confirmed at day 580 at 2.5, thus it is classified as RAW with confirmed event score 2.5, and both references are reset to 2.5 (post-event re-baselining). The increase at day 760 satisfies the minimum increase condition with respect to the RAW/PIRA reference, is confirmed at the next assessment, and neither the increase nor its confirmation are in proximity of a relapse, thus the event is classified as PIRA. The references are set to 3.5 (the confirmed event score). The increase at day 880 satisfies the minimum increase condition with respect to this new reference, but since its confirmation assessment lies in the RAW window of a subsequent relapse, the event is classified as PIRA with relapse during confirmation.

# References

1. Müller J, Cagol A, Lorscheider J, et al. Harmonizing Definitions for Progression Independent of Relapse Activity in Multiple Sclerosis: A Systematic Review. JAMA Neurol. 2023;80.

2. Müller J, Sharmin S, Lorscheider J, et al. Standardized Definition of Progression Independent of Relapse Activity (PIRA) in Relapsing-Remitting Multiple Sclerosis. JAMA Neurol. 2025;82.
